# Design, Synthesis, and Anti-Tyrosinase, Anti-Melanogenic, and Antioxidant Activities of Novel *(Z)*-3-Benzyl-5-Benzylidene-2-Thioxothiazolidin-4-One Analogs

**DOI:** 10.3390/molecules30030517

**Published:** 2025-01-23

**Authors:** Hyeon Seo Park, Hee Jin Jung, Hye Soo Park, Hye Jin Kim, Yujin Park, Pusoon Chun, Hae Young Chung, Hyung Ryong Moon

**Affiliations:** 1 Department of Manufacturing Pharmacy, College of Pharmacy and Research Institute for Drug Development, Pusan National University, Busan 46241, Republic of Korea; gustj6956@pusan.ac.kr (H.S.P.); hjjung2046@pusan.ac.kr (H.J.J.); hyesoo0713@pusan.ac.kr (H.S.P.); khj3358@pusan.ac.kr (H.J.K.); 2 Department of Medicinal Chemistry, New Drug Development Center, Daegu-Gyeongbuk Medical Innovation Foundation, Daegu 41061, Republic of Korea; pyj1016@kmedihub.re.kr; 3 College of Pharmacy and Inje Institute of Pharmaceutical Sciences and Research, Inje University, Gimhae 50834, Republic of Korea; pusoon@inje.ac.kr; 4 Department of Pharmacy, College of Pharmacy and Research Institute for Drug Development, Pusan National University, Busan 46241, Republic of Korea; hyjung@pusan.ac.kr

**Keywords:** melanin, tyrosinase, B16F10 cells, β-phenyl-α,β-unsaturated carbonyl, *(Z)*-BBTT

## Abstract

To discover novel anti-melanogenic compounds with tyrosinase inhibitory activity, *(Z)*-3-benzyl-5-benzylidene-2-thioxothiazolidin-4-one (*(Z)*-BBTT) analogs **1**–**12**, designed based on the hybrid structure of a β-phenyl-α,β-unsaturated carbonyl motif and a 3-benzyl-2-thioxothiazolidin-4-one scaffold, were synthesized as novel tyrosinase inhibitors. Of the 12 analogs, 2 (**6** and **8**) showed mushroom tyrosinase inhibitory activity similar to that of kojic acid, a representative tyrosinase inhibitor, and 3 analogs (**1**–**3**) exhibited mushroom tyrosinase inhibitory activity that was more potent than that of kojic acid. In particular, analog **3** revealed highly potent inhibition with an IC_50_ value of 90 nM, which was 214 times lower than that of kojic acid (IC_50_ value = 19.22 μM). A kinetic study using mushroom tyrosinase and analogs **1**–**3** and **6** demonstrated that these analogs were competitive inhibitors, which was further supported by in silico studies. Analogs **1** and **3** have strong anti-melanogenic potency in B16F10 mammalian cells owing to their anti-tyrosinase activity without perceptible cytotoxicity in melanoma cells (B16F10) and the main epidermal cells (HaCaT). Moreover, analog **3** exhibited strong antioxidant capacity, scavenging reactive oxygen species, 2,2′-azino-bis(3-ethylbenzothiazoline-6-sulfonic acid) cation radical, and 2,2-diphenyl-1-picrylhydrazyl radical, partially contributing to its anti-melanogenic effect. *(Z)*-BBTT analogs, including analog **3**, may be promising candidates for inhibiting melanin production.

## 1. Introduction

Ultraviolet (UV) rays are classified into UV-A (320–400 nm), UV-B (290–320 nm), and UV-C (200–290 nm), depending on wavelength [1]. UV-C, which has the highest energy and shortest wavelength, is blocked by the Earth’s atmosphere, including the ozone layer, and barely reaches the Earth’s surface. By contrast, UV-A and UV-B, which have longer wavelengths, reach the Earth’s surface and exert various effects on living organisms. In humans, UV-B is essential for the photolysis of 7-dehydrocholesterol in the skin for the synthesis of previtamin D3 [2,3]. Although UV rays can have positive effects on living organisms, they can destroy keratinocytes and fibroblasts in the epidermis and dermis, respectively, thereby causing wrinkles. Melanin, a polymer containing indole or thiazine rings, is produced in the skin upon UV radiation exposure. It has a chemical structure that absorbs or scatters UV rays, thus protecting the skin cells [4]. Indeed, melanin production is a defense mechanism of cells for protection against UV rays [5]. However, excessive melanin production in the skin can cause various cosmetic problems such as age spots, freckles, melasma, actinic keratosis, lentigines, skin hyperpigmentation diseases, and even skin cancer [6,7,8]. Therefore, the demand is continuous for the discovery of new and more effective anti-melanin ingredients for skin beauty and treatments related to skin hyperpigmentation.

Melanin is synthesized through various enzymatic and nonenzymatic reactions in melanosomes—organelles within melanocytes in the skin epidermis. Synthesized melanin is then transferred to the surrounding keratinocytes and is the most important factor in determining skin color [9]. In addition to neuromelanin, which exists in nerve cells, two types of melanin are found in humans. Eumelanin has a relatively dark color (black to brown), whereas pheomelanin has a relatively light color (yellow to red) [10,11]. These two melanins are biosynthesized from a common intermediate, dopaquinone. Pheomelanin is synthesized from dopaquinone solely through nonenzymatic chemical reactions, whereas eumelanin is synthesized through both nonenzymatic and enzymatic chemical reactions catalyzed by tyrosinase-related protein-1 (TRP-1), TRP-2, and tyrosinase [12]. Dopaquinone is synthesized from l-tyrosine via two sequential enzymatic oxidative reactions. These processes are rate-limiting steps, and the two oxidation reactions are catalyzed by the same enzyme, tyrosinase. Although several strategies can inhibit melanin production, tyrosinase inhibition is the most studied and is recognized as the most effective method because it participates in the rate-limiting step of melanin biosynthesis. Tyrosinase oxidizes l-tyrosine to l-DOPA through monophenolase activity and subsequently oxidizes l-DOPA to dopaquinone through diphenolase activity [13,14,15]. Dopaquinone is converted to pheomelanin in the presence of thiol compounds such as cysteine or to dopachrome through an intramolecular auto-reaction and then to eumelanin through several consecutive reactions [12,16]. Dopaquinone is very rapidly converted into dopachrome, which can strongly absorb light at 475 nm. Thus, a widely used method to assess the effect of a compound on tyrosinase activity is to measure its absorbance at 475 nm [17].

**Figure 1:**
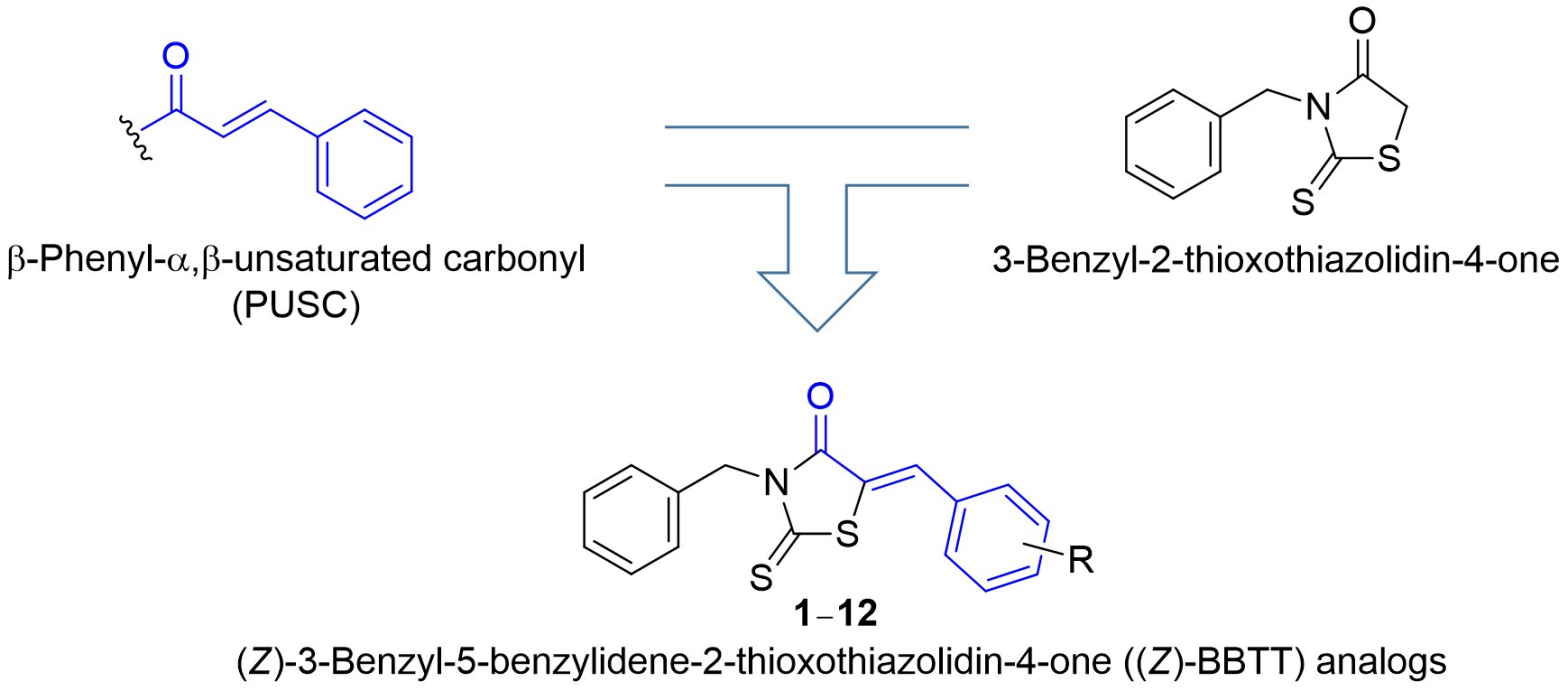
Chemical structures of β-phenyl-α,β-unsaturated carbonyl (PUSC) and lipophilic 3-benzyl-2-thioxothiazolidin-4-one, and the final target compounds, *(Z)*-3-benzyl-5-benzylidene-2-thioxothiazolidin-4-one (*(Z)*-BBTT) analogs created by the structural hybrid of PUSC and 3-benzyl-2-thioxothiazolidin-4-one.

Compounds containing 3-benzyl-2-thioxothiazolidin-4-one have exhibited a variety of biological activities, including antiviral [18], anticancer [19], antibacterial [20], glycogen synthase kinase 3 inhibition [21], urease inhibition [22], anti-inflammatory [23], antituberculosis [24], and antifungal [20] activities. The β-phenyl-α,β-unsaturated carbonyl (PUSC) motif was confirmed to act as an important chemical skeleton of tyrosinase inhibitors (Figure 1). In particular, when the phenyl moiety of the PUSC motif has a hydroxyl substituent at both positions 2 and 4, the compound has good tyrosinase inhibitory activity [25,26,27,28,29]. Topical skin-whitening agents would be advantageous if they are slightly hydrophobic, allowing them to be easily absorbed into the skin. Therefore, to synthesize novel PUSC-type tyrosinase inhibitors with hydrophobic properties, lipophilic 3-benzyl-2-thioxothiazolidin-4-one was designed as an active methylene compound that reacts with various benzaldehydes, and *(Z)*-3-benzyl-5-benzylidene-2-thioxothiazolidin-4-one (*(Z)*-BBTT) analogs **1**–**12** were synthesized as the final target compounds (Figure 1). The ability of *(Z)*-BBTT analogs to inhibit tyrosinases was investigated in mushroom and murine cells, and their capacity to inhibit melanin production was examined in murine cells. Additionally, antioxidant activity of these analogs was evaluated by their potency to remove 2,2′-azino-bis(3-ethylbenzothiazoline-6-sulfonic acid) cation (ABTS^+^) radical, 2,2-diphenyl-1-picrylhydrazyl (DPPH) radical, and reactive oxygen species (ROS). Moreover, the tyrosinase inhibitory mode of action of *(Z)*-BBTT analogs and the chemical interactions between tyrosinase and these analogs were examined.

## 2. Results and Discussion

### 2.1. Synthesis of Target Compounds (Z)-BBTT Analogs ***1**–**12***

**Scheme 1:**
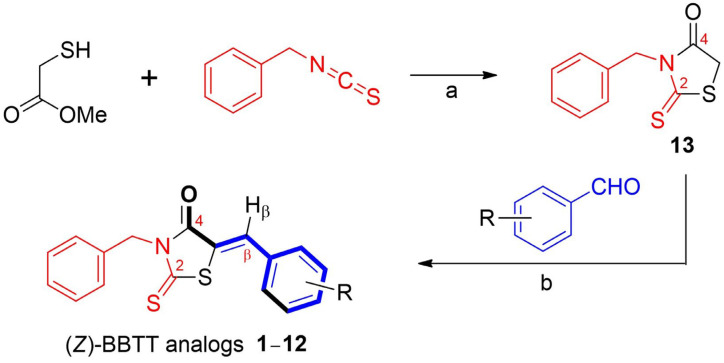
Synthetic scheme of *(Z)*-3-benzyl-5-benzylidene-2-thioxothiazolidin-4-one (*(Z)*-BBTT) analogs **1**–**12**. The reagents and conditions were as follows: (a) Et_3_N, CH_2_Cl_2_, RT, 3.5 h, and 94% and (b) NaOAc, AcOH, reflux, 2–4 h, and 47–92%.

To synthesize the target compounds (*(Z)*-BBTT analogs **1**–**12**), 3-benzyl-2-thioxothiazolidin-4-one (**13**) was used as a key intermediate for condensation with various benzaldehydes (Scheme 1). Compound **13** was prepared via the condensation of benzyl isothiocyanate and methyl 2-mercaptoacetate in the presence of trimethylamine in dichloromethane. Purification of **13** was performed by filtration and then washing with diethyl ether and hexane to remove impurities. In the ^13^C nuclear magnetic resonance (NMR) spectrum, the C2 and C4 peaks of **13** appeared at 201.1 and 174.0 ppm, respectively, confirming the construction of a 2-thioxothiazolidin-4-one ring. The synthesis of final target analogs **1**–**12** was achieved using a Knoevenagel condensation reaction: the reflux of **13** and twelve benzaldehydes (1.0 equiv.) with various substituents in the presence of sodium acetate (6.0 equiv.) in acetic acid. Purification of **1**–**12** was performed by filtration and then washing with dichloromethane and hexane to remove impurities. The synthetic yields ranged from 47% to 92%. The trisubstituted alkenes generated by the Knoevenagel condensation reaction were (*Z*)-isomers. There is a paper that reported obtaining *(Z)*-isomers through a Knoevenagel condensation reaction of **13** and various benzaldehydes [19]. *(Z)*-isomers have less steric hindrance than the corresponding *(E)*-isomers and are thermodynamically more stable than the latter owing to intramolecular H-bonding between the carbonyl oxygen and vinylic H (H_β_). In addition, the ^3^*J*_C4-Hβ_ values of C4 obtained in a proton-coupled ^13^C-mode NMR indicated that the trisubstituted exocyclic methylene geometry is *(Z)*-stereochemistry. Vogeli et al. reported that the ^3^*J*_C4-Hβ_ is 3.5–7.0 Hz when the carbonyl and β-H in trisubstituted alkenes of α,β-unsaturated carbonyl compounds are on the same side and is generally ≥10 Hz when they are on opposite sides [30]. The C4 peak of analog **3** appeared as a doublet (5.8 Hz) of triplets (2.9 Hz) owing to coupling by protons of β–H and N–CH_2_, indicating that the carbonyl and β–H are on the same side.

### 2.2. Inhibitory Activity of (Z)-BBTT Analogs ***1**–**12*** Against Mushroom Tyrosinase

The mushroom tyrosinase inhibitory activity of *(Z)*-BBTT analogs **1**–**12** was evaluated in the presence of two substrates, l-tyrosine and l-DOPA, by measuring the optical density of the generated dopachrome at 475 nm. Kojic acid, a well-known tyrosinase inhibitor, was used as a standard to compare the inhibitory activity.

The tyrosinase inhibitory activities of the *(Z)*-BBTT analogs were assessed in the presence of l-tyrosine. Kojic acid revealed an IC_50_ value of 19.22 μM, and two *(Z)*-BBTT analogs, **6** with 4-methoxyphenyl and **8** with 2,4-dimethoxyphenyl, showed potent mushroom tyrosinase inhibitory activity similar to that of kojic acid (IC_50_ values of **6** and **8**: 19.11 and 18.57 μM, respectively) (Table 1). In contrast, analog **7** (IC_50_ value: 56.19 μM) containing 3,4-dimethoxyphenyl inhibited tyrosinase activity to a lesser extent than kojic acid. *(Z)*-BBTT analogs **1**–**3**, which have only hydroxyl substituents on the β-phenyl ring of *(Z)*-BBTT analogs, exhibited a stronger tyrosinase inhibitory effect compared with kojic acid. Analog **1** with 4-hydroxyphenyl had an IC_50_ value of 4.69 μM, which was four-fold lower than that of kojic acid. Insertion of an additional hydroxyl substituent into position 3 of the β-phenyl ring in **1** did not affect the tyrosinase inhibitory effect (IC_50_ value of **2** with 3,4-dihydroxyphenyl: 5.75 μM). However, insertion of an additional hydroxyl substituent into position 2 of the β-phenyl ring in **1** greatly increased the tyrosinase inhibitory activity (IC_50_ value of **3** with 2,4-dihydroxyphenyl: 0.09 μM). The inhibitory potency of analog **3** was 52 and 214 times stronger than that of analog **1** and kojic acid, respectively. Meanwhile, three analogs (**4**, **5**, and **11**) with one hydroxyl substituent and one methoxyl or bromo substituent exhibited slightly lower tyrosinase inhibitory activity than kojic acid (IC_50_ values of **4** with 4-hydroxy-3-methoxyphenyl, **5** with 3-hydroxy-4-methoxyphenyl, and **11** with 3-bromo-4-hydroxyphenyl: 45.52, 39.01, and 66.26 μM, respectively). Analog **9** containing trimethoxyphenyl and analogs **10** and **12** containing hydroxyl surrounded by two methoxyl or bromo groups on the β-phenyl ring had IC_50_ values > 200 μM.

In addition, the tyrosinase inhibitory activity of the *(Z)*-BBTT analogs was assessed in the presence of l-DOPA. Most analogs showed lower mushroom tyrosinase inhibitory activity when using the l-DOPA substrate than when using the l-tyrosine substrate (Table 1). An exception was analog **9,** containing 3,4,5-trimethoxyphenyl, which exhibited stronger tyrosinase inhibitory activity in the presence of l-DOPA than in the presence of l-tyrosine (IC_50_ values of **9**: 97.23 μM in the presence of l-DOPA vs. >200 μM in the presence of l-tyrosine). Kojic acid showed tyrosinase inhibitory potency with an IC_50_ value of 26.54 μM. Analogs **1**, **2**, and **6** showed potent tyrosinase inhibitory activity either similar to or slightly weaker than that of kojic acid (IC_50_ values of **1** with 4-hydroxyphenyl, **2** with 3,4-dihydroxyphenyl, and **6** with 4-methoxyphenyl: 38.10, 27.47, and 33.54 μM, respectively). Similar to that in the presence of l-tyrosine, analogs **2** and **3**, which contain two hydroxyl substituents on the β-phenyl ring, showed differences in tyrosinase inhibition depending on the relative position of the two hydroxyls. Analog **2** with 3,4-dihydroxyphenyl demonstrated tyrosinase inhibitory activity similar to that of kojic acid. Meanwhile, analog **3** with 2,4-dihydroxyphenyl showed notably more potent tyrosinase inhibitory activity compared with kojic acid, with approximately 100 times greater potency (IC_50_ value: 0.27 μM) than kojic acid. Analogs **7** and **8**, obtained by replacing the hydroxyls in analogs **2** and **3** with methoxyls, decreased the tyrosinase inhibitory activity (IC_50_ values of **7** with 3,4-dimethoxyphenyl and **8** with 2,4-dimethoxyphenyl were 70.64 and 51.14 μM, respectively). Analogs **4**, **5**, and **11**, which have one hydroxyl substituent and one methoxyl or bromo substituent, showed moderate tyrosinase inhibitory effects, with IC_50_ values ranging from 90.01 to 121.24 μM. By contrast, analogs **10** and **12**, which have one hydroxyl substituent and two methoxyl or bromo substituents, exhibited tyrosinase inhibitory activity with IC_50_ values > 200 μM, as observed in the presence of l-tyrosine.

Table 2 shows that when a hydroxyl substituent was positioned at R^4^ in the *(Z)*-BBTT structure, the addition of an additional hydroxyl substituent at R^2^ and R^3^ significantly and slightly increased the tyrosinase inhibitory activity, respectively. However, when a methoxyl substituent was positioned at R^4^, the insertion of an additional hydroxyl substituent at R^3^ decreased the tyrosinase inhibitory activity. In the presence of l-tyrosine, the introduction of a hydroxyl substituent at R^4^ resulted in a greater tyrosinase inhibitory effect than the introduction of a methoxyl substituent. However, in the presence of l-DOPA, these two substituents showed similar tyrosinase inhibitory activities.

### 2.3. Kinetic Analysis of Mushroom Tyrosinase Using Lineweaver–Burk Plots

As analogs **1**–**3** and **6** had potent inhibitory activity against mushroom tyrosinase, their inhibitory mechanisms were investigated by measuring the initial formation rate of dopachrome in the presence of l-DOPA. Analogs were used at 0, 20, 40, and 80 μM for **1**; 0, 15, 30, and 60 μM for **2**; 0, 0.1, 0.2, and 0.4 μM for **3**; and 0, 12.5, 25, and 50 μM for **6**. l-DOPA was used at concentrations of 1, 2, 4, 8, and 16 mM.

**Figure 2:**
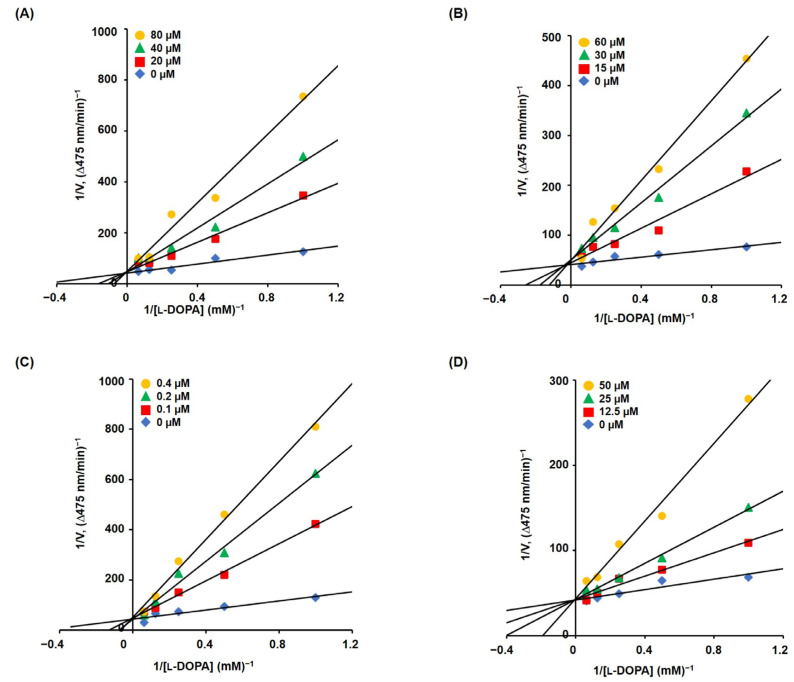
Lineweaver–Burk plots for **1**–**3** (**A**–**C**) and **6** (**D**) obtained by measuring the initial dopachrome production rate in the presence of various l-DOPA concentrations (1, 2, 4, 8, and 16 mM) using mushroom tyrosinase. The concentrations of the test analogs were 0, 20, 40, and 80 μM for **1**; 0, 15, 30, and 60 μM for **2**; 0, 0.1, 0.2, and 0.4 μM for **3**; and 0, 12.5, 25, and 50 μM for **6**.

The four Lineweaver–Burk plots for **1**–**3** and **6** are shown in Figure 2. All Lineweaver–Burk plots had four lines with different slopes and merged at one point on the y-axis, indicating that all analogs were competitive inhibitors that bound to the tyrosinase active site and competed with the substrates l-DOPA and l-tyrosine.

**Figure 3:**
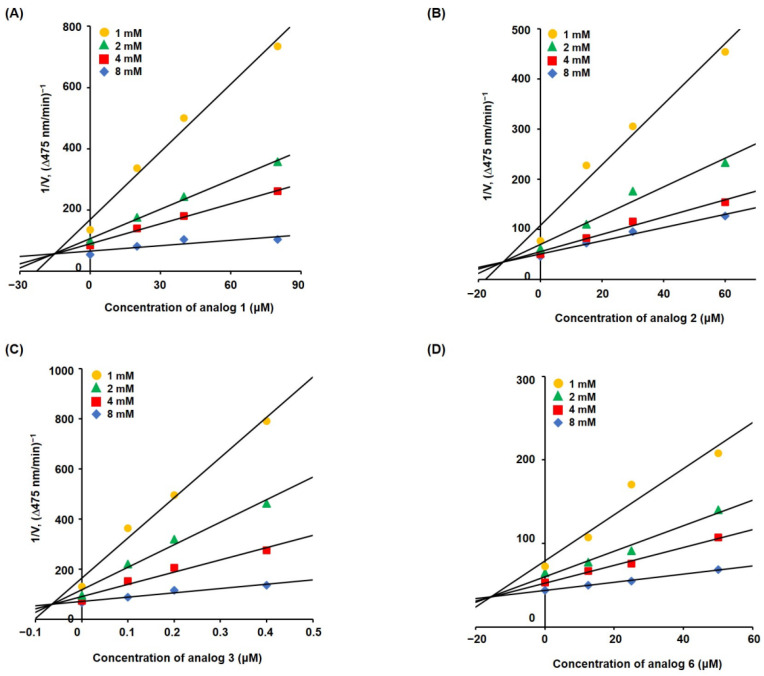
Dixon plots for analogs **1**–**3** (**A**–**C**) and **6** (**D**) obtained by converting Lineweaver–Burk plots. The concentrations of the test analogs were 0, 20, 40, and 80 μM for **1**; 0, 15, 30, and 60 μM for **2**; 0, 0.1, 0.2, and 0.4 μM for **3**; and 0, 12.5, 25, and 50 μM for **6**. l-DOPA was used at concentrations of 1, 2, 4, and 8 mM.

To determine the inhibition constant (K_i_), Lineweaver–Burk plots for analogs **1**–**3** and **6** were converted to the corresponding Dixon plots by plotting the analog concentrations against the inverse of the initial dopachrome production rate. Each Dixon plot provided a picture of four straight lines that merged at one point in the second quadrant (Figure 3). The absolute value of the x-coordinate of the merged point is the K_i_ value. The K_i_ values for analogs **1**–**3** and **6** were 15.5, 12.2, 0.064, and 15.5 μM, respectively, indicating that analog **3** had the greatest binding affinity with tyrosinase among the analogs.

### 2.4. In Silico Docking Simulation of (Z)-BBTT Analogs and Mushroom Tyrosinase

Docking simulations with mushroom tyrosinase were performed for analogs **1**–**3** and **6,** which showed excellent inhibitory activities against mushroom tyrosinase. Kojic acid and AutoDock Vina 1.2.0 were used as the positive control and docking simulation software, respectively. To obtain information on the chemical interactions between tyrosinase amino acid residues and ligands (analogs **1**–**3** and **6** and kojic acid), LigandScout 4.4.8 (Inte: Ligand GmbH, Vienna, Austria) was used.

**Figure 4:**
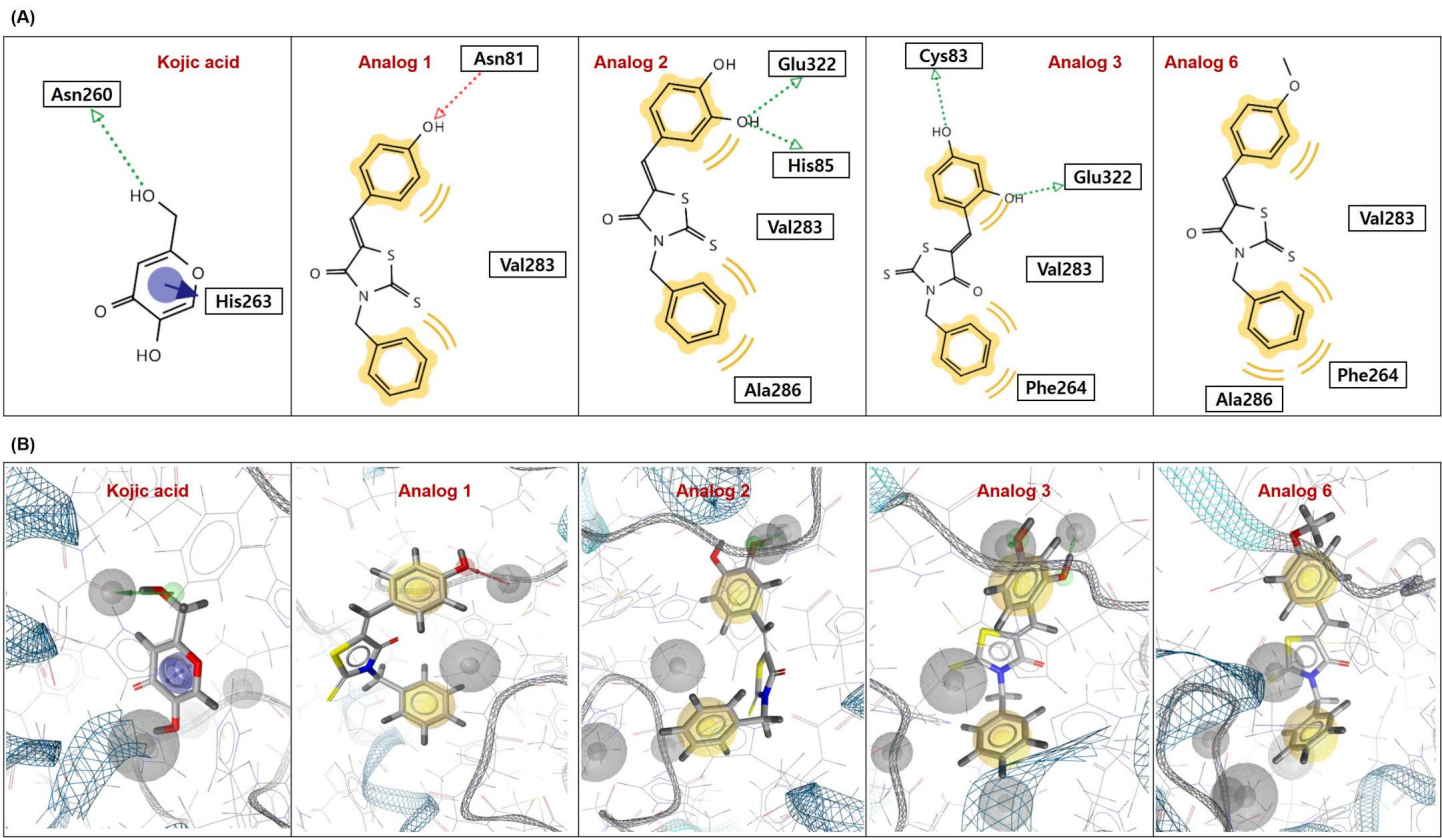
Chemical interactions between tyrosinase and ligands (kojic acid [positive control] and analogs **1**–**3** and **6**) obtained from LigandScout after docking simulation using AutoDock Vina. (**A**) Two- and (**B**) three-dimensional pictures. Blue arrow, pi–pi stacking; yellow wave, hydrophobic interaction; green arrow, hydrogen bonding (H–B donor from the ligand’s perspective); and red arrow, hydrogen bonding (H–B acceptor from the ligand’s perspective). (**B**) In molecules, red, blue, and yellow represent oxygen, nitrogen, and sulfur, respectively.

Figure 4 shows the docking simulation results. All docked compounds bound to the tyrosinase active site. Kojic acid participated in hydrogen bonding (H–B) of the 2-hydroxymethyl with Asn260 as an H–B donor and in pi–pi stacking of the pyran-4-one with His263, providing a binding energy of −5.4 kcal/mol. Analog **1** participated in one H–B of the 4-hydroxy group with Asn81 as an H–B acceptor and in hydrophobic interactions of the β-phenyl and benzyl rings with Val283, providing a binding energy of −7.5 kcal/mol. Analog **2** formed two hydrogen bonds between the 3-hydroxy moiety and two amino acids (Glu322 and His85) and participated in hydrophobic interactions between the β-phenyl and benzyl rings and amino acids (Val283 and Ala286). These chemical interactions contributed to a binding energy of −7.8 kcal/mol. Each hydroxyl substituent of analog **3** created an H–B: the 2-hydroxyl substituent that interacted with Glu322 and the 4-hydroxyl substituent that interacted with Cys83. Additionally, analog **3** generated hydrophobic interactions between the β-phenyl ring and Val283 and between the benzyl ring and two amino acids (Val283 and Phe264). These chemical interactions provided a binding energy of −7.8 kcal/mol. Analog **6** only formed hydrophobic interactions between the β-phenyl and benzyl rings and amino acids (Val283, Phe264, and Ala286), providing −7.6 kcal/mol binding energy. All four *(Z)*-BBTT analogs showed stronger binding affinities than kojic acid. Val283 participated in hydrophobic interactions with all *(Z)*-BBTT analogs, implying that Val283 acts as an important amino acid in the inhibition of tyrosinase activity by *(Z)*-BBTT analogs. Glu322 participated in the formation of H–Bs with analogs **2** and **3**, suggesting that Glu322 is also a key amino acid that exerts a tyrosinase inhibitory effect.

### 2.5. Cell Viability of (Z)-BBTT Analogs on B16F10 Cells

Because *(Z)*-BBTT analogs **1**–**3** exhibited significant mushroom tyrosinase inhibitory activity, the cell viability for these analogs was assessed prior to investigating their cellular tyrosinase activity and ability to inhibit melanin production in B16F10 cells. Cell viability for analogs **1**–**3** was examined on B16F10 cells at 1, 2, 5, and 10 μM concentrations for 72 h.

**Figure 5:**
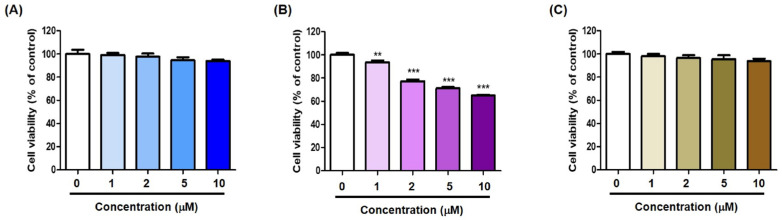
B16F10 cell viability in the presence of *(Z)*-3-benzyl-5-benzylidene-2-thioxothiazolidin-4-one analogs **1** (**A**), **2** (**B**), and **3** (**C**). *** *p* < 0.001 and ** *p* < 0.01 vs. the nontreated group.

The cytotoxicity results are shown in Figure 5. Analog **2** was cytotoxic at all tested concentrations, whereas analogs **1** and **3** were not cytotoxic to B16F10 cells up to 10 μM, which is the highest tested concentration. Thus, analogs **1** and **3** were used at concentrations ≤10 μM in B16F10-cell-based experiments measuring melanin contents and cellular tyrosinase activity.

### 2.6. Effect of (Z)-BBTT Analogs ***1*** and ***3*** on Melanin Production in B16F10 Cells

The effects of analogs **1** and **3** on melanin production were examined in B16F10 cells using kojic acid as a positive control. The analogs were administered at concentrations of 2, 5, and 10 μM, and kojic acid was used at 10 μM. Stimulators comprising 1 μM α-melanocyte-stimulating hormone (α-MSH) and 200 μM 3-isobutyl-1-methylxanthine (IBMX) were administered 1 h after test sample treatment. The effects of analogs **1** and **3** on melanin production were determined by measuring the optical density at 405 nm after 72 h of treatment.

**Figure 6:**
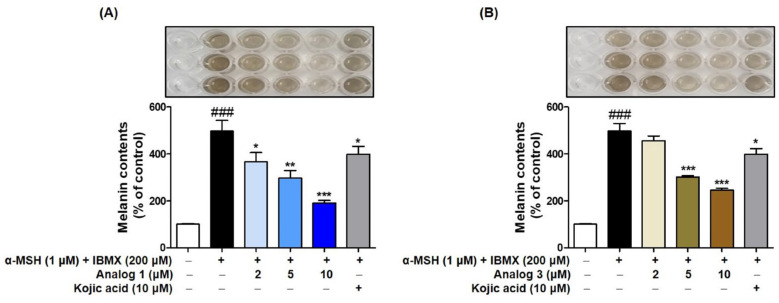
Effect of analogs **1** (**A**) and **3** (**B**) on melanin production in B16F10 cells. Stimulators α-MSH and IBMX were treated at 1 and 200 μM, respectively, 1 h after treatment of analogs (2, 5, and 10 μM). After 72 h, melanin contents were determined by measuring the optical density at 405 nm. Kojic acid was used as a positive control. α-MSH, α-melanocyte-stimulating hormone; IBMX, 3-isobutyl-1-methylxanthine. *** *p* < 0.001, ** *p* < 0.01, and * *p* < 0.05 vs. stimulator-treated group; ^###^ *p* < 0.001 vs. untreated control.

Treatment with stimulators largely increased melanin contents (5.0-fold increase), and exposure to kojic acid significantly reduced the stimulator-induced increased melanin content by 4.0-fold (Figure 6). Analogs **1** and **3** significantly decreased the stimulator-induced increase in melanin contents in a concentration-dependent manner. Analog **1** showed a slightly more potent anti-melanogenic effect than analog **3**. Analog **1** at 2 μM reduced melanin content to levels similar to that of 10 μM kojic acid, and at 10 μM, it decreased the stimulator-induced melanin contents by 1.9-fold. Analog **3** at 2 μM inhibited melanin production more weakly than 10 μM kojic acid, although it inhibited melanin production significantly more potently than kojic acid at the same concentration (10 μM).

### 2.7. Effect of (Z)-BBTT Analogs ***1*** and ***3*** on Tyrosinase Activity in B16F10 Cells

To examine whether the anti-melanogenic effects of analogs **1** and **3** observed in Figure 6 were attributable to their ability to inhibit B16F10 cellular tyrosinase activity, the effects of analogs **1** and **3** on cellular tyrosinase activity were assessed in B16F10 cells. Similar to that in the melanin content experiments, kojic acid (10 μM) was used as a positive control. B16F10 cells were pretreated with an analog (**1** and **3**) at 2, 5, and 10 μM or kojic acid and then exposed to stimulators (1 μM α-MSH and 200 μM IBMX) 1 h later. The cellular tyrosinase inhibitory activity of the test samples was determined by measuring their optical density at 475 nm 72 h after test sample treatment.

**Figure 7:**
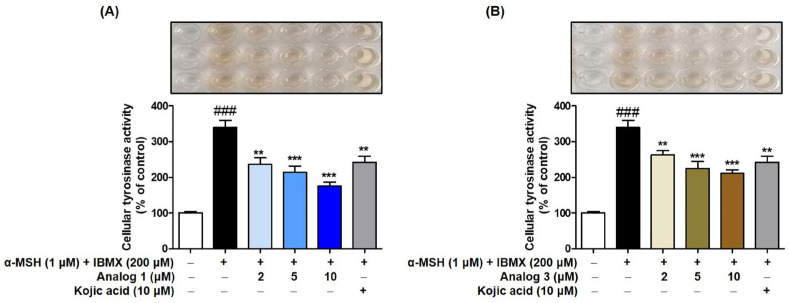
Effects of analogs **1** (**A**) and **3** (**B**) on B16F10 cellular tyrosinase activity. Stimulators α-MSH and IBMX were treated at 1 and 200 μM, respectively, 1 h after treatment of analogs (2, 5, and 10 μM). After 72 h, tyrosinase activity was determined by measuring optical density at 475 nm. α-MSH, α-melanocyte-stimulating hormone; IBMX, 3-isobutyl-1-methylxanthine. *** *p* < 0.001 and ** *p* < 0.01 vs. stimulator-treated group; ^###^ *p* < 0.001 vs. untreated control.

Stimulator treatment enhanced cellular tyrosinase activity by 3.4-fold compared with that in the control group (100%) (Figure 7). However, kojic acid exposure greatly reduced the stimulator-enhanced cellular tyrosinase activity by 2.4-fold. Treatment with analogs **1** and **3** also significantly decreased the stimulator-enhanced cellular tyrosinase activity in a concentration-dependent manner. At 10 μM, both analogs **1** and **3** inhibited cellular tyrosinase activity more strongly than kojic acid. The pattern of cellular tyrosinase inhibition was similar to that of melanogenesis inhibition, indicating that the anti-melanogenic effect of *(Z)*-BBTT analogs **1** and **3** was primarily attributable to their ability to inhibit tyrosinase activity.

### 2.8. In Situ B16F10 Cell Tyrosinase Inhibitory Activity of (Z)-BBTT Analogs ***1*** and ***3***

To identify the in situ cellular tyrosinase inhibitory activity of analogs **1** and **3**, B16F10 cells and l-DOPA were used as the experimental target cells and substrate for melanin synthesis, respectively. Kojic acid (10 μM) was used for comparing the inhibitory activity. B16F10 cells were treated with test samples (**1**, **3**, and kojic acid) for 1 h, and then α-MSH (1 μM) and IBMX (200 μM) were administered for 72 h to increase cellular tyrosinase activity. B16F10 cells were photographed after 2 h of treatment with excess l-DOPA, and the darkness of the cells was measured using the CS analyzer program (ATTO, Tokyo, Japan). Analogs **1** and **3** were used at 2, 5, and 10 μM.

**Figure 8:**
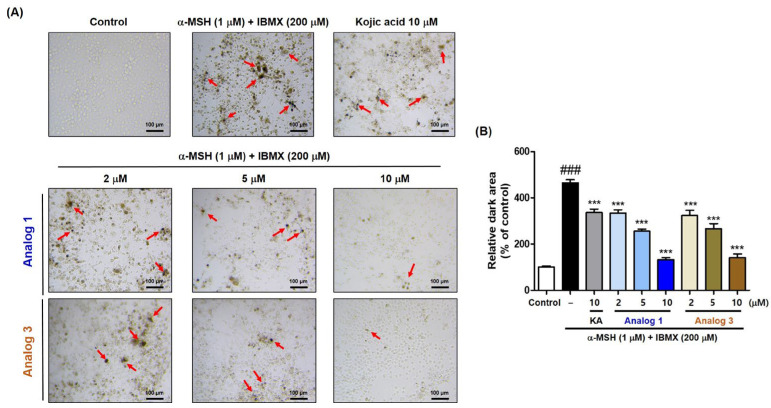
(**A**) In situ B16F10 cell tyrosinase activity determined using an l-DOPA staining method. Kojic acid (10 μM) was used as a positive reference standard. Test samples (kojic acid and analogs **1** and **3**) were administered 1 h before treatment with α-MSH (1 μM) and IBMX (200 μM). The analogs were treated at 2, 5, and 10 μM. The results of in situ cellular tyrosinase activity were obtained 72 h after treatment with the analogs or kojic acid. Arrows indicate stained cells. Scale bar = 100 μm. (**B**) Relative amounts of stained areas were measured using an ATTO CS analyzer 3.2. α-MSH, α-melanocyte-stimulating hormone; IBMX, 3-isobutyl-1-methylxanthine; KA, kojic acid. ^###^ *p* < 0.001 vs. control; *** *p* < 0.001 vs. α-MSH plus IBMX-treated group.

Treatment with α-MSH and IBMX produced many cells with excessive melanin, whereas exposure to kojic acid significantly reduced the number of cells strongly stained with melanin (Figure 8). Analogs **1** and **3** reduced the number of strongly stained cells in a concentration-dependent manner. According to the results measuring relative dark areas of cells, the dark areas of **1** and **3** at 2 μM were similar to that of kojic acid at 10 μM, and when compared at the same concentration (10 μM), the dark area of each analog (**1** and **3**) was significantly less than that of kojic acid. Thus, analogs **1** and **3** effectively inhibited tyrosinase activity in mammalian B16F10 cells.

### 2.9. Antioxidant Capacity of (Z)-BBTT Analogs ***1**–**12***

As the antioxidant capacity of a compound is closely related to its anti-melanogenic effect [31], the ability of *(Z)*-BBTT analogs **1**–**12** to remove representative oxidant species such as ABTS^+^ and DPPH radicals and ROS was investigated.

#### 2.9.1. ABTS^+^ Radical-Scavenging Capacity

To measure the scavenging effects of analogs **1**–**12** on ABTS^+^ radicals, ABTS was initially treated with potassium persulfate to yield ABTS^•+^. The formed ABTS^+^ radical solution was mixed with test samples (analogs **1**–**12** and Trolox [positive reference standard]; final concentration: 100 μM), and absorbance was measured at 732 nm after storing for 2 min in the dark to determine %ABTS^•+^-scavenging ability.

**Figure 9:**
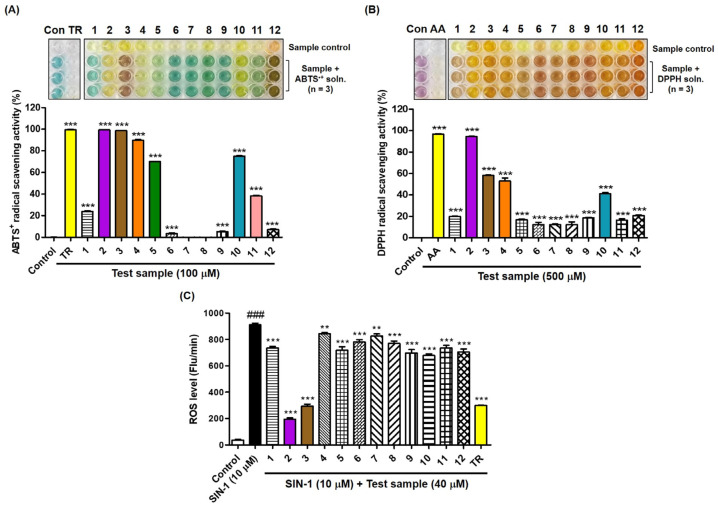
Antioxidant effects of *(Z)*-BBTT analogs **1**–**12** on radicals of ABTS^+^ (**A**) and DPPH (**B**) and ROS (**C**). (**A**) Analogs **1**–**12** and Trolox (TR; positive reference standard) were used at 100 μM. (**B**) Analogs **1**–**12** and ascorbic acid (AA; positive reference standard) were used at 500 μM. (**C**) Analogs **1**–**12**, Trolox (TR; positive reference standard), and SIN-1, a ROS generator, were used at 40, 40, and 10 μM, respectively. In (**A**,**B**), *** *p* < 0.001 vs. the control group and in (**C**), ** *p* < 0.01 and *** *p* < 0.001 vs. the SIN-1-treated group; ^###^ *p* < 0.001 vs. the control group. *(Z)*-BBTT, *(Z)*-3-benzyl-5-benzylidene-2-thioxothiazolidin-4-one; ABTS^+^; 2,2′-azino-bis(3-ethylbenzothiazoline-6-sulfonic acid) cation; DPPH, 2,2-diphenyl-1-picrylhydrazyl; SIN-1, 3-morpholinosydnonimine; ROS, reactive oxygen species.

Trolox exhibited strong ABTS^•+^scavenging activity (99% scavenging), and five analogs (**2**–**5** and **10**) of **1**–**12** also showed strong ABTS^•+^ inhibitory activity (>70%) (Figure 9A). In particular, analogs **2**–**4** showed very potent ABTS^•+^ inhibitory efficacy with % scavenging activity ≥ 90%. Analogs **2** and **3** with catechol and resorcinol groups, respectively, had high potency with 99% and 98% ABTS^•+^-scavenging abilities, respectively, which were comparable to that of Trolox. Generally, analogs bearing a hydroxyl substituent on the β-phenyl ring showed potent ABTS^•+^-scavenging activity, whereas analogs without a hydroxyl substituent on the β-phenyl ring showed little ABTS^•+^-scavenging activity (**2**–**5**, **10**, and **11** vs. **6**–**9**).

#### 2.9.2. DPPH Radical-Scavenging Capacity

For measuring the scavenging effect of analogs **1**–**12** on DPPH radicals, test samples (analogs **1**–**12** and ascorbic acid [positive reference standard]; final concentration: 500 μM) were mixed with a DPPH solution, and absorbance was measured at 517 nm after storing for 30 min in the dark to determine % DPPH radical-scavenging ability.

Ascorbic acid and analog **2** bearing a catechol group showed strong radical-scavenging potential with 97 and 95% DPPH radical-scavenging abilities, respectively (Figure 9B). Analogs **3**, **4**, and **10**, which exerted potent ABTS^•+^-scavenging activity, also scavenged DPPH radicals by 41–58%. DPPH and ABTS^+^ radical-scavenging experiments showed similar trends; however, analog **5** showed a notable difference. Analog **5** demonstrated strong ABTS^•+^ inhibitory activity with 71% ABTS^•+^-scavenging; however, it showed only 17% DPPH radical-scavenging activity, which was almost similar to that of analogs **6**–**9** without a hydroxyl substituent on the β-phenyl ring.

#### 2.9.3. ROS-Scavenging Capacity

UV-A radiation induces ROS generation substantially, and oxidative stress accelerates melanin formation in organisms [32,33]. Thus, the ROS-scavenging ability of *(Z)*-BBTT analogs was examined to determine whether these analogs have an auxiliary ability to scavenge ROS. 3-Morpholinosydnonimine (SIN-1; 10 μM) was used as a ROS generating substance, and Trolox (40 μM) was used as a positive reference standard.

SIN-1 exposure significantly enhanced ROS levels, and treatment with Trolox significantly reduced the SIN-1-induced ROS levels (Figure 9C). All *(Z)*-BBTT analogs significantly reduced the SIN-1-induced ROS levels; however, most analogs weakly scavenged ROS except analogs **2** and **3** with 3,4-dihydroxy and 2,4-dihydroxy on the β-phenyl ring, respectively. Analog **3** showed ROS-scavenging activity equipotent to that of Trolox, whereas analog **2** exhibited greater ROS-scavenging potency than Trolox. The ROS-scavenging ability of these analogs may partially contribute to the inhibition of tyrosinase activity and melanogenesis.

### 2.10. Effects of Analogs ***1***–***3*** on HaCaT Cell Viability

As keratinocytes are one of the major cells that constitute the epidermis, the effects of analogs **1**–**3** on HaCaT (immortalized human keratinocytes) cell viability were investigated. Analogs **1**–**3** were tested at concentrations of 0, 2, 5, 10, and 20 μM, and cell viability was determined 72 h after incubation.

**Figure 10:**
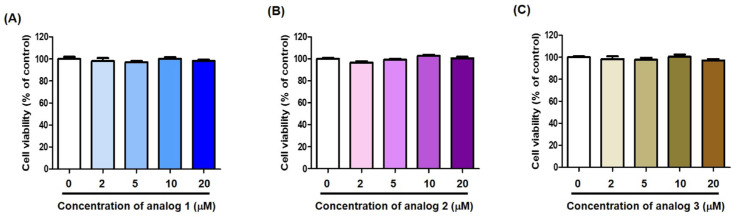
HaCaT cell viability in the presence of analogs **1**–**3** (**A**–**C**). All analogs were administered at concentrations of 0, 2, 5, 10, and 20 μM for 72 h.

None of the analogs showed significant cytotoxicity at any concentration tested (Figure 10). These results suggest that analogs **1** and **3** are promising candidates for skin-lightening agents, as they exhibit potent melanin production inhibition effects without perceptible cytotoxicity in the primary cells that constitute the epidermis.

## 3. Materials and Methods

### 3.1. Synthesis

#### 3.1.1. General Methods

Thin-layer chromatography (TLC; Merck TLC Silica gel 60 F_254,_ Darmstadt, Germany) was used to monitor the progress of the reactions. Chemical reagents were obtained from Daejung Chemicals (Siheung-si, Republic of Korea) and SEJIN CI Co., Ltd. (Seoul, Republic of Korea). All solvents were prepared in an anhydrous manner by distillation over Na/benzophenone or CaH_2_. Melting point of compounds were measured using a Stuart Scientific SMP3 Melting Point apparatus (Scientific Laboratory Supplies Ltd., Nottingham, UK). An expression compact mass spectrometer (Advion, Itahca, NY, USA) was utilized to obtain low-resolution mass (LR-MS) data, which were measured in the electrospray ionization negative or positive mode. High-resolution mass (HR-MS) data were acquired on a ZenoTOF 7600 mass spectrometer (SCIEX, Framingham, MA, USA). NMR data were obtained using a Varian Unity AS500 spectrometer (Agilent Technologies, Santa Clara, CA, USA) for 500 MHz ^1^H-NMR and 125 MHz ^13^C-NMR or a JEOL ECZ400S instrument (JEOL Ltd., Tokyo, Japan) for 400 MHz ^1^H-NMR and 100 MHz ^13^C-NMR. The chemical shifts (δ) and coupling constants (J) were recorded as ppm and Hz units, respectively. Peak splitting patterns were recorded as s (singlet), d (doublet), m (multiplet), brs (broad singlet), brd (broad doublet), or dd (doublet of doublets).

#### 3.1.2. Synthesis of 3-Benzyl-2-Thioxothiazolidin-4-One (**13**) [34,35]

Methyl thioglycolate (1.4 mL, 15.10 mmol) and triethylamine (2.1 mL, 15.07 mmol) were slowly added to a solution of benzyl isothiocyanate (2.0 mL, 15.08 mmol) in dichloromethane (DCM; 40 mL) at 0 °C. The reaction mixture was stirred at room temperature for 3.5 h. After the volatiles were evaporated in vacuo, a small amount of ether and appropriate amounts of hexane were added to the resulting residue to form solids. The generated solids were filtered and washed with a mixture of ether and hexane (1:5) to afford pure 3-benzyl-2-thioxothiazolidin-4-one (**13**; 3.17 g, 94%).

^1^H NMR (CDCl_3_, 400 MHz) δ 7.44–7.42 (dd, 2H, J = 7.6, 1.6 Hz, 2-H, 6-H), 7.33–7.28 (m, 3H, 3-H, 4-H, 5-H), 5.18 (s, 2H, NCH_2_), 3.97 (s, 2H, SCH_2_); ^13^C NMR (CDCl_3_, 100 MHz) δ 201.1, 174.0, 134.8, 129.1, 128.7, 128.3, 47.7, 35.5; molecular formula, C_10_H_9_NOS_2_; melting point, 80–81 °C; black-gray solid; Rf (hexane:ethyl acetate = 4:1 on silica gel TLC) = 0.42.

#### 3.1.3. General Preparation of *(Z)*-BBTT Analogs **1**–**12**

A solution of compound **13** (80 mg, 0.36 mmol) and an appropriate benzaldehyde (1.0 equiv.) in acetic acid (0.8 mL) was refluxed in sodium acetate (176 mg, 2.15 mmol) for 2–4 h. After cooling, cold water was added to the reaction mixtures. The generated solid was filtered and washed with water and, if necessary, additionally washed with DCM and hexane to furnish **1**–**12** in yields of 47–92%. All products were sufficiently pure for biological experiments.

- *(Z)*-3-Benzyl-5-(4-hydroxybenzylidene)-2-thioxothiazolidin-4-one (**1**).
  ^1^H NMR (dimethyl sulfoxide [DMSO]-d_6_, 500 MHz) δ 10.51 (s, 1H, OH), 7.76 (s, 1H, vinyl H), 7.52 (d, 2H, J = 8.5 Hz, 2′-H, 6′-H), 7.35–7.26 (m, 5H, Ph), 6.94 (d, 2H, J = 8.5 Hz, 3′-H, 5′-H), 5.23 (s, 2H, benzyl H_2_); ^13^C NMR (DMSO-d_6_, 125 MHz) δ 193.7, 167.5, 161.2, 135.5, 134.7, 133.9, 129.0, 128.1 (2 × C), 124.4, 117.9, 117.1, 47.5; LR-MS (ESI−) *m*/*z* 326 (M − H)^−^; yield, 76%; molecular formula, C_17_H_13_NO_2_S_2_; HR-MS (EDA) *m*/*z* C_17_H_14_NO_2_S_2_ (M + H)^+^ calcd. 328.0466, obsd. 328.0460; melting point, 199–201 °C; brown solid; Rf (hexane:ethyl acetate = 3:1 on silica gel TLC) = 0.38.
- *(Z)*-3-Benzyl-5-(3,4-dihydroxybenzylidene)-2-thioxothiazolidin-4-one (**2**).
  ^1^H NMR (DMSO-d_6_, 500 MHz) δ 10.06 (s, 1H, OH), 9.59 (s, 1H, OH), 7.68 (s, 1H, vinyl H), 7.35–7.25 (m, 5H, Ph), 7.08–7.04 (m, 2H, 2′-H, 6′-H), 6.90 (d, 1H, J = 8.5 Hz, 5′-H), 5.23 (s, 2H, benzyl H_2_); ^13^C NMR (DMSO-d_6_, 125 MHz) δ 193.7, 167.5, 150.1, 146.6, 135.5, 135.1 (2 × C), 129.0, 128.1, 125.9, 124.8, 117.7, 117.2, 117.0, 47.5; LR-MS (ESI−) *m*/*z* 342 (M − H)^−^; yield, 84%; molecular formula, C_17_H_13_NO_3_S_2_; HR-MS (EDA) *m*/*z* C_17_H_14_NO_3_S_2_ (M + H)^+^ calcd. 344.0415, obsd. 344.0410; melting point, 140–142 °C; brown solid; Rf (hexane:ethyl acetate = 3:1 on silica gel TLC) = 0.10.
- *(Z)*-3-Benzyl-5-(2,4-dihydroxybenzylidene)-2-thioxothiazolidin-4-one (**3**).
  ^1^H NMR (DMSO-d_6_, 500 MHz) δ 10.76 (s, 1H, OH), 10.42 (s, 1H, OH), 7.99 (s, 1H, vinyl H), 7.35–7.25 (m, 5H, Ph), 7.22 (d, 1H, J = 8.5 Hz, 6′-H), 6.45–6.43 (m, 2H, 3′-H, 5′-H), 5.22 (s, 2H, benzyl H_2_); ^13^C NMR (DMSO-d_6_, 125 MHz) δ 193.9, 167.7, 163.3, 160.5, 135.6, 132.1, 130.4, 128.9, 128.0 (2 × C), 115.6, 112.4, 109.5, 102.9, 47.3; LR-MS (ESI−) *m*/*z* 342 (M − H)^−^; yield, 74%; molecular formula, C_17_H_13_NO_3_S_2_; HR-MS (EDA) *m*/*z* C_17_H_14_NO_3_S_2_ (M + H)^+^ calcd. 344.0415, obsd. 344.0412; melting point, 191–193 °C; copper-colored solid; Rf (hexane:ethyl acetate = 3:1 on silica gel TLC) = 0.10.
- *(Z)*-3-Benzyl-5-(4-hydroxy-3-methoxybenzylidene)-2-thioxothiazolidin-4-one (**4**).
  ^1^H NMR (CDCl_3_, 500 MHz) δ 7.67 (s, 1H, vinyl H), 7.47 (d, 2H, J = 7.0 Hz, 2-H, 6-H), 7.33–7.28 (m, 3H, 3-H, 4-H, 5-H), 7.08 (brd, 1H, J = 8.0 Hz, 6′-H), 7.00 (d, 1H, J = 8.0 Hz, 5′-H), 6.95 (s, 1H, 2′-H), 6.04 (s, 1H, OH), 5.32 (s, 2H, benzyl H_2_), 3.95 (s, 3H, OCH_3_); ^13^C NMR (CDCl_3_, 125 MHz) δ 193.0, 167.9, 148.6, 147.1, 134.9, 133.9, 129.0, 128.6, 128.1, 126.4, 126.0, 119.8, 115.4, 112.0, 56.1, 47.5; LR-MS (ESI−) *m*/*z* 356 (M − H)^−^; yield, 68%; molecular formula, C_18_H_15_NO_3_S_2_; melting point, 157–159 °C (lit. [19] 151–154 °C); brown solid; Rf (hexane:ethyl acetate = 3:1 on silica gel TLC) = 0.29.
- *(Z)*-3-Benzyl-5-(3-hydroxy-4-methoxybenzylidene)-2-thioxothiazolidin-4-one (**5**).
  ^1^H NMR (CDCl_3_, 500 MHz) δ 7.64 (s, 1H, vinyl H), 7.47 (d, 2H, J = 7.0 Hz, 2-H, 6-H), 7.33–7.27 (m, 3H, 3-H, 4-H, 5-H), 7.07–7.04 (m, 2H, 2′-H, 6′-H), 6.92 (d, 1H, J = 8.0 Hz, 5′-H), 5.72 (s, 1H, OH), 5.32 (s, 2H, benzyl H_2_), 3.95 (s, 3H, OCH_3_); ^13^C NMR (CDCl_3_, 125 MHz) δ 193.3, 168.0, 148.9, 146.2, 134.9, 133.6, 129.0, 128.6, 128.1, 126.9, 124.6, 120.7, 116.1, 111.0, 56.2, 47.5; LR-MS (ESI−) *m*/*z* 356 (M − H)^−^; yield, 54%; molecular formula, C_18_H_15_NO_3_S_2_; melting point, 173–175 °C; yellow solid; Rf (hexane:ethyl acetate = 3:1 on silica gel TLC) = 0.28.
- *(Z)*-3-Benzyl-5-(4-methoxybenzylidene)-2-thioxothiazolidin-4-one (**6**).
  ^1^H NMR (CDCl_3_, 500 MHz) δ 7.70 (s, 1H, vinyl H), 7.47 (d, 2H, J = 7.0 Hz, 2-H, 6-H), 7.45 (d, 2H, J = 9.0 Hz, 2′-H, 6′-H), 7.33–7.27 (m, 3H, 3-H, 4-H, 5-H), 6.99 (d, 2H, J = 9.0 Hz, 3′-H, 5′-H), 5.32 (s, 2H, benzyl H_2_), 3.87 (s, 3H, OCH_3_); ^13^C NMR (CDCl_3_, 125 MHz) δ 193.2, 168.0, 161.8, 135.0, 133.5, 132.8, 129.0, 128.6, 128.1, 126.0, 119.9, 115.0, 55.6, 47.5; yield, 69%; molecular formula, C_18_H_15_NO_2_S_2_; HR-MS (EDA) *m*/*z* C_18_H_16_NO_2_S_2_ (M + H)^+^ calcd. 342.0622, obsd. 342.0619; melting point, 146–148 °C (lit. [19] 142–145 °C); copper-colored solid; Rf (hexane:ethyl acetate = 3:1 on silica gel TLC) = 0.63.
- *(Z)*-3-Benzyl-5-(3,4-dimethoxybenzylidene)-2-thioxothiazolidin-4-one (**7**).
  ^1^H NMR (CDCl_3_, 500 MHz) δ 7.69 (s, 1H, vinyl H), 7.47 (d, 2H, J = 7.0 Hz, 2-H, 6-H), 7.33–7.27 (m, 3H, 3-H, 4-H, 5-H), 7.13 (d, 1H, J = 8.5 Hz, 6′-H), 6.97 (s, 1H, 2′-H), 6.95 (d, 1H, J = 8.5 Hz, 5′-H), 5.32 (s, 2H, benzyl H_2_), 3.94 (s, 6H, 2 × OCH_3_); ^13^C NMR (CDCl_3_, 125 MHz) δ 193.0, 167.9, 151.6, 149.5, 134.9, 133.7, 129.0, 128.6, 128.1, 126.3, 125.7, 120.1, 112.4, 111.5, 56.1, 56.0, 47.5; LR-MS (ESI+) *m*/*z* 372 (M + H)^+^, 394 (M + Na)^+^; yield, 52%; molecular formula, C_19_H_17_NO_3_S_2_; melting point, 166–168 °C; yellow solid; Rf (hexane:ethyl acetate = 3:1 on silica gel TLC) = 0.45.
- *(Z)*-3-Benzyl-5-(2,4-dimethoxybenzylidene)-2-thioxothiazolidin-4-one (**8**).
  ^1^H NMR (CDCl_3_, 500 MHz) δ 8.07 (s, 1H, vinyl H), 7.48 (d, 2H, J = 7.5 Hz, 2-H, 6-H), 7.33–7.25 (m, 4H, 3-H, 4-H, 5-H, 6′-H), 6.57 (dd, 1H, J = 9.0, 2.0 Hz, 5′-H), 6.45 (d, 1H, J = 2.0 Hz, 3′-H), 5.32 (s, 2H, benzyl H_2_), 3.88 (s, 3H, OCH_3_), 3.86 (s, 3H, OCH_3_); ^13^C NMR (CDCl_3_, 125 MHz) δ 194.0, 168.1, 163.9, 160.4, 135.1, 132.1, 129.6, 129.0, 128.5, 128.0, 119.4, 115.7, 105.9, 98.5, 55.7, 55.6, 47.5; LR-MS (ESI+) *m*/*z* 372 (M + H)^+^, 394 (M + Na)^+^; yield, 81%; molecular formula, C_19_H_17_NO_3_S_2_; melting point, 156–158 °C; brown solid; Rf (hexane:ethyl acetate = 3:1 on silica gel TLC) = 0.76.
- *(Z)*-3-Benzyl-2-thioxo-5-(3,4,5-trimethoxybenzylidene)thiazolidin-4-one (**9**).
  ^1^H NMR (CDCl_3_, 500 MHz) δ 7.65 (s, 1H, vinyl H), 7.46 (d, 2H, J = 7.0 Hz, 2-H, 6-H), 7.33–7.25 (m, 3H, 3-H, 4-H, 5-H), 6.70 (s, 2H, 2′-H, 6′-H), 5.32 (s, 2H, benzyl H_2_), 3.92 (s, 3H, OCH_3_), 3.91 (s, 6H, 2 × OCH_3_); ^13^C NMR (CDCl_3_, 125 MHz) δ 192.9, 167.7, 153.7, 140.6, 134.8, 133.6, 129.0, 128.7, 128.6, 128.2, 121.8, 107.9, 61.1, 56.3, 47.6; LR-MS (ESI+) *m*/*z* 402 (M + H)^+^, 424 (M + Na)^+^; yield, 78% molecular formula, C_20_H_19_NO_4_S_2_; melting point, 108–110 °C; copper-colored solid; Rf (hexane:ethyl acetate = 3:1 on silica gel TLC) = 0.78.
- *(Z)*-3-Benzyl-5-(4-hydroxy-3,5-dimethoxybenzylidene)-2-thioxothiazolidin-4-one (**10**).
  ^1^H NMR (CDCl_3_, 500 MHz) δ 7.63 (s, 1H, vinyl H), 7.46 (d, 2H, J = 7.0 Hz, 2-H, 6-H), 7.33–7.25 (m, 3H, 3-H, 4-H, 5-H), 6.71 (s, 2H, 2′-H, 6′-H), 5.96 (s, 1H, OH), 5.31 (s, 2H, benzyl H_2_), 3.94 (s, 6H, 2 × OCH_3_); ^13^C NMR (CDCl_3_, 125 MHz) δ 192.9, 167.8, 147.5, 137.9, 134.9, 134.0, 128.9, 128.6, 128.1, 124.9, 120.1, 107.8, 56.5, 47.5; LR-MS (ESI+) *m*/*z* 388 (M + H)^+^, 410 (M + Na)^+^; LR-MS (ESI−) *m*/*z* 386 (M − H)^−^; yield, 92%; molecular formula, C_19_H_17_NO_4_S_2_; melting point, 151–153 °C; copper-colored solid; Rf (hexane:ethyl acetate = 3:1 on silica gel TLC) = 0.68.
- *(Z)*-3-Benzyl-5-(3-bromo-4-hydroxybenzylidene)-2-thioxothiazolidin-4-one (**11**).
  ^1^H NMR (CDCl_3_, 500 MHz) δ 7.62 (s, 1H, 2′-H), 7.60 (s, 1H, vinyl H), 7.46 (d, 2H, J = 7.0 Hz, 2-H, 6-H), 7.37 (d, 1H, J = 8.5 Hz, 6′-H), 7.33–7.27 (m, 3H, 3-H, 4-H, 5-H), 7.11 (d, 1H, J = 8.5 Hz, 5′-H), 5.93 (brs, 1H, OH), 5.32 (s, 2H, benzyl H_2_); ^13^C NMR (CDCl_3_, 125 MHz) δ 192.6, 167.8, 154.4, 134.8, 134.5, 131.9, 131.4, 129.0, 128.6, 128.2, 127.6, 121.8, 117.0, 111.4, 47.6; LR-MS (ESI−) *m*/*z* 404 (M − H)^−^, 406 (M − H + 2)^−^; yield, 47%; molecular formula, C_17_H_12_BrNO_2_S_2_; melting point, 154–156 °C; yellow solid; Rf (hexane:ethyl acetate = 3:1 on silica gel TLC) = 0.34.
- *(Z)*-3-Benzyl-5-(3,5-dibromo-4-hydroxybenzylidene)-2-thioxothiazolidin-4-one (**12**).
  ^1^H NMR (DMSO-d_6_, 500 MHz) δ 10.95 (brs, 1H, OH), 7.81 (s, 2H, 2′-H, 6′-H), 7.74 (s, 1H, vinyl H), 7.36–7.26 (m, 5H, Ph), 5.23 (s, 2H, benzyl H_2_); ^13^C NMR (DMSO-d_6_, 125 MHz) δ 193.1, 167.2, 153.7, 135.3, 134.8, 131.3 (2 × C), 129.0, 128.1, 127.8, 121.8, 112.9, 47.6; LR-MS (ESI−) *m*/*z* 482 (M − H)^−^, 484 (M − H + 2)^−^, 486 (M − H + 4)^−^; yield, 80%; molecular formula, C_17_H_11_Br_2_NO_2_S_2_; melting point, 179–181 °C; copper-colored solid; Rf (hexane:ethyl acetate = 3:1 on silica gel TLC) = 0.26.

### 3.2. Mushroom Tyrosinase Inhibition Assay [36,37]

The inhibitory effects of the *(Z)*-BBTT analogs **1**–**12** on mushroom tyrosinase were examined in the presence of l-DOPA and l-tyrosine as substrates. Kojic acid and *(Z)*-BBTT analogs **4**–**12** were used at 2, 10, and 50 μM regardless of substrates used; *(Z)*-BBTT analogs **1** and **2** were used at 0.4, 2, and 10 μM in the presence of l-tyrosine and 2, 10, and 50 μM in the presence of l-DOPA; and *(Z)*-BBTT analog **3** was used at 0.016, 0.08, and 0.4 μM in the presence of l-tyrosine and 0.08, 0.4, and 2 μM in the presence of l-DOPA to determine their IC_50_ values. An aliquot (10 μL) of kojic acid or *(Z)*-BBTT analogs was mixed with mushroom tyrosinase aqueous solution (20 μL; 800 units/mL) and substrate mixture (170 μL) consisting of 17.2 mM sodium phosphate buffer (pH 6.5) and 345 μM l-tyrosine or l-DOPA in each well of a 96-well plate. The plate was placed at 37 °C for 30 min, and well absorbance was measured at 475 nm to determine the generated dopachrome amounts in each well using a VersaMax^®^ Elisa (VE) microplate reader (Molecular Devices, San Jose, CA, USA).

### 3.3. Kinetic Experiment in the Presence of (Z)-BBTT Analogs ***1**–**3*** and ***6*** Using Mushroom Tyrosinase [38,39,40]

Kinetic experiments were performed by measuring the initial dopachrome generation rate of mushroom tyrosinase. An aliquot (10 μL) of *(Z)*-BBTT analogs **1**–**3** and **6** was mixed with mushroom tyrosinase aqueous solution (20 μL; 150 units/mL) and substrate mixture (170 μL) consisting of 17.2 mM sodium phosphate buffer (pH 6.5) and l-DOPA aqueous solution of various concentrations (1, 2, 4, 8, and 16 mM) in each well of a 96-well plate. During the 30 min incubation, well absorbance was recorded at 475 nm every 5 min using a VE microplate reader. Analogs **1**–**3** and **6** were used at the following concentrations: 0, 20, 40, and 80 μM for **1**; 0, 15, 30, and 60 μM for **2**; 0, 0.1, 0.2, and 0.4 μM for **3**; and 0, 12.5, 25, and 50 μM for **6**. The Lineweaver–Burk plot for each analog was obtained by plotting the reciprocal of the initial dopachrome formation rate versus the reciprocal of the substrate concentration.

### 3.4. Docking Simulation of Mushroom Tyrosinase and (Z)-BBTT Analogs ***1**–**3*** and ***6*** [41,42]

For the in silico docking simulation between mushroom tyrosinase and the *(Z)*-BBTT analogs **1**–**3** and **6**, the three-dimensional (3D) X-ray structure of the mushroom tyrosinase protein was acquired from the Protein Data Bank: 2Y9X (*Agaricus bisporus*) (http://www.rcsb.org, accessed on 1 September 2024). The cocrystal structure of 2Y9X contains tropolone as the ligand at the active site. Kojic acid was used as a positive reference standard. The 3D structures of the ligands (kojic acid and analogs **1**–**3** and **6**) were acquired through energy minimization using Chem3D Pro 12.0. After removing tropolone bound to the tyrosinase active site, the 3D structure of tyrosinase was docked with the ligand 3D structures using AutoDock Vina (ver. 1.2.0). The binding energies between the ligands and tyrosinase were determined using AutoDock Vina and Chimera 1.17.3 (https://www.cgl.ucsf.edu/chimera/download.html, accessed on 15 October 2024). LigandScout 4.4.8 (http://www.inteligand.com/ligandscout/download.shtml, accessed on 20 October 2024) provided the plausible chemical interactions between the ligands and tyrosinase.

### 3.5. Cell Culture [42,43]

Murine B16F10 and human keratinocyte HaCaT cells were cultivated in a solution consisting of Dulbecco’s modified Eagle’s medium (Welgene, Gyeongsan-si, Republic of Korea), 10% heat-inactivated fetal bovine serum (Gibco, Grand Island, NY, USA), and 1% penicillin–streptomycin solution (100×) at 37 °C in a humidified environment with 5% CO_2_.

### 3.6. Cytotoxicity Assay on B16F10 Murine Melanoma Cells [44]

The influence of *(Z)*-BBTT analogs **1**–**3** on B16F10 cell viability was studied using an EZ-Cytox solution (EZ-1000^®^, DoGenBio, Seoul, Republic of Korea). B16F10 cells were seeded at a density of 1 × 10^3^ in each well of a 96-well plate, and the plate was cultivated under normal cell culture conditions for 24 h. Analogs were added to each well at concentrations of 0, 1, 2, 5, and 10 μM, and then cells were cultivated for 72 h under normal cell culture conditions. An aliquot (10 μL) of the EZ-Cytox solution was added to each well and cultivated for 2 h. Well absorbance was recorded at 450 nm using a VE microplate reader to calculate cell viability.

### 3.7. Cellular Melanin Level Measurement in the Presence of (Z)-BBTT Analogs ***1*** and ***3*** in B16F10 Cells [44]

B16F10 cells were seeded at a density of 5000 per well of a 6-well plate, and the plate was cultivated under normal cell culture conditions for 24 h. Kojic acid was utilized for comparing activities. Analog (**1** and **3**) or kojic acid was added to each well at concentrations of 0, 2, 5, and 10 μM and 10 μM, respectively, and then cultivated for 1 h under normal cell culture conditions. In addition, 200 μM IBMX and 1 μM α-MSH (Alomone Labs™, Jerusalem, Israel) were added to each well and cultivated for 72 h under the normal cell culture conditions. To determine the cellular melanin levels, 1N–NaOH solution (100 μL) was added to each well and then kept at 60 °C for 1 h to lyse the cells. The cell lysates were washed twice with phosphate-buffered saline (PBS) and transferred to a 96-well plate, and the absorbance was recorded at 405 nm using a VE microplate reader to determine the cellular melanin content.

### 3.8. Cellular Tyrosinase Activity Measurement in the Presence of (Z)-BBTT Analogs ***1*** and ***3*** in B16F10 Cells [44]

B16F10 cells were seeded at a density of 5000 per well of a 6-well plate, and the plate was cultivated under normal cell culture conditions for 24 h. Kojic acid was utilized for comparing activities. Analog (**1** and **3**) or kojic acid was added to each well at concentrations of 0, 2, 5, and 10 μM and 10 μM, respectively, and then cultivated for 1 h under normal cell culture conditions. Stimulators (200 μM IBMX and 1 μM α-MSH) were added to each well and cultivated for 72 h under normal cell culture conditions. To determine cellular tyrosinase activity, the cells were washed twice with PBS, treated with lysis buffer solution (100 μL) comprising 50 mM phosphate buffer (pH 6.5) (90 μL), 2 mM phenylmethanesulfonyl fluoride (5 μL), and 20% Triton X-100 (5 μL), and incubated at −80 °C for 30 min. After centrifugation (10,000× *g*, 4 °C, and 10 min), the supernatants (80 μL each) were transferred to each well of a 96-well plate, then 10 mM l-DOPA (20 μL) was added to each well. The absorbance was measured at 475 nm at 1 min intervals for 10 min using a VE microplate reader to determine cellular tyrosinase activity.

### 3.9. Measurement of In Situ Cellular Tyrosinase Activity in B16F10 Cells [44,45]

To evaluate in situ cellular tyrosinase activity, test samples (kojic acid [positive reference standard] and analogs **1** and **3**) were added to each well of a 24-well plate containing B16F10 cells incubated for 24 h at a density of 1 × 10^3^ cells/well. Kojic acid and the two analogs were used at final concentrations of 10 and 2, 5, and 10 μM, respectively. After 1 h, the cells were exposed to 1 μM α-MSH and 200 μM IBMX and cultivated under normal cell culture conditions for 72 h. Cultured cells were fixed using a 4% paraformaldehyde solution for 40 min, washed twice with PBS, and permeabilized using 0.1% Triton X-100 for 2 min. l-DOPA solution (2 mM; 500 μL) was added to each well after washing twice with PBS. After the plate was placed for 2 h at 37 °C, the images for cell staining were acquired using a camera attached to a microscope (Motic, Hong Kong).

### 3.10. ABTS^•+^ Scavenging Activity [46,47]

ABTS^•+^ was prepared by mixing equal amounts of ABTS (14 mM in H_2_O) and K_2_S_2_O_8_ (4.9 mM in H_2_O) and storing in the dark at 20 °C for 16 h. Prior to use, the absorbance of the prepared ABTS^•+^ solution was adjusted to 0.7 ± 0.02 at 732 nm by dilution with methanol. A cosolvent (EtOH:DMSO = 10:1 [*v*/*v*]) was used to prepare solutions of the test samples (analogs **1**–**12** and Trolox [positive reference standard]). All test samples were used at a final concentration of 100 μM. The test sample solution (10 μL) was mixed with the adjusted ABTS^•+^ solution (90 μL) in each well of a 96-well plate, and the mixture was kept in the dark at 20 °C for 2 min. The absorbance was recorded at 732 nm at 1 min intervals for 10 min using a VE microplate reader. The percentage of radical-scavenging activity was calculated using the formula % radical-scavenging activity = [(Abs_control_ − Abs_sample_)/Abs_control_] × 100, where Abs_sample_ and Abs_control_ are absorbances of the sample and control, respectively.

### 3.11. DPPH Radical-Scavenging Activity [48,49]

l-Ascorbic acid was used as a positive control. Test samples (analogs **1**–**12** and l-ascorbic acid; 5 mM, 20 μL) dissolved in DMSO were added to each well of a 96-well plate containing the DPPH methanol solution (0.2 mM, 180 μL), and the plate was placed in the dark at 20 °C for 30 min. The absorbance was recorded at 517 nm using a VE microplate reader to calculate DPPH radical-scavenging activity.

### 3.12. ROS-Scavenging Activity [50,51]

The test samples (*(Z)*-BBTT analogs **1**–**12** and Trolox [positive reference standard]) and 3-morpholinosydnonimine (SIN-1) were dissolved in DMSO and PBS, respectively. To prepare 2′,7′-dichlorodihydrofluorescein (DCFH) solution, PBS (50 mM; 4.9 mL, pH 7.4) and DCFH diacetate (2.5 mM; 0.05 mL) were mixed with esterase (15 units/10 mL; 0.05 mL). After storage for 30 min at 20 °C, the mixture was placed in the dark until further use. SIN-1 (10 µL) was added to each well of a 96-well black plate containing PBS (180 µL) and test sample (10 µL), and the plate was placed in the dark for 5 min. The prepared DCFH solution (50 µL) was added to each well of the black plate. The fluorescence of each well was recorded at 535 nm using a microplate reader (Berthold Advances GmbH & Co., Bad Wildbad, Germany) at an excitation wavelength of 485 nm. The test samples and SIN-1 were tested at final concentrations of 40 and 10 μM, respectively.

### 3.13. Cytotoxicity Assay on HaCaT Cells [42]

The effect of *(Z)*-BBTT analogs **1**–**3** on HaCaT cell viability was examined using EZ-Cytox solution. HaCaT cells were seeded at a density of 1 × 10^3^ in each well of a 96-well plate, and the plate was cultivated under normal cell culture conditions for 24 h. Analogs **1**–**3** were added to each well at concentrations of 0, 2, 5, 10, and 20 μM and then cultivated for 72 h under normal cell culture conditions. An aliquot (10 μL) of the EZ-Cytox solution was added to each well and cultivated for 2 h. Well absorbance was recorded at 450 nm using a VE microplate reader to calculate HaCaT cell viability.

### 3.14. Statistical Analysis

All assays were performed independently at least three times to determine statistical significance. The results are described as means ± standard errors of the mean (SEM). Significance was determined using GraphPad Prism 5 (La Jolla, CA, USA) via a one-way analysis of variance (ANOVA) followed by the Newman–Keuls test. Significance was set at *p* < 0.05.

## 4. Conclusions

*(Z)*-BBTT analogs **1**–**12** were designed and synthesized as novel tyrosinase inhibitors based on the structures of a β-phenyl-α,β-unsaturated carbonyl motif and a 3-benzyl-2-thioxothiazolidin-4-one scaffold. Analogs **1**–**3** exhibited strong inhibitory activity against mushroom tyrosinase. In particular, the IC_50_ value of analog **3** was found to be in the range of tens of nanomoles. Kinetic studies demonstrated analogs **1**–**3** and **6** to be competitive inhibitors, and in silico studies supported the results. Experiments using B16F10 mammalian cells demonstrated that analogs **1** and **3** strongly inhibited melanin production through their anti-tyrosinase activities. Furthermore, analogs **1** and **3** showed no perceptible cytotoxicity in HaCaT and B16F10 cells, and analog **3** exhibited strong antioxidant efficacy in various antioxidant experiments.

## Figures and Tables

**Table 1:** Mushroom tyrosinase inhibitory activity of *(Z)*-3-benzyl-5-benzylidene-2-thioxothiazolidin-4-one (*(Z)*-BBTT) analogs **1**–**12** in the presence of its substrates l-tyrosine and l-DOPA.

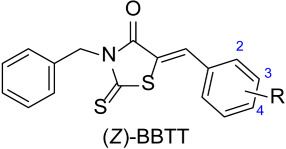			
Compound	R	IC_50_ (μM)	
		l-Tyrosine	l-Dopa
^a^ Kojic acid		19.22 ± 1.29	26.54 ± 0.17
**1**	4-Hydroxy	4.69 ± 0.58	38.10 ± 2.26
**2**	3,4-Dihydroxy	5.75 ± 0.81	27.47 ± 1.84
**3**	2,4-Dihydroxy	0.09 ± 0.01	0.27 ± 0.04
**4**	4-Hydroxy-3-methoxy	45.52 ± 2.49	121.24 ± 4.90
**5**	3-Hydroxy-4-methoxy	39.01 ± 2.15	91.01 ± 8.55
**6**	4-Methoxy	19.11 ± 2.03	33.54 ± 0.30
**7**	3,4-Dimethoxy	56.19 ± 1.98	70.64 ± 1.25
**8**	2,4-Dimethoxy	18.57 ± 1.23	51.14 ± 2.05
**9**	3,4,5-Trimethoxy	>200	97.23 ± 7.73
**10**	4-Hydroxy-3,5-dimethoxy	>200	>200
**11**	3-Bromo-4-hydroxy	66.26 ± 2.11	114.88 ± 2.28
**12**	3,5-Dibromo-4-hydroxy	>200	>200

^a^ Kojic acid was used as a standard for comparing inhibitory activity.

**Table 2:** Structure–activity relationship between substitutions on the β-phenyl ring of *(Z)*-BBTT analogs and tyrosinase inhibition activity.

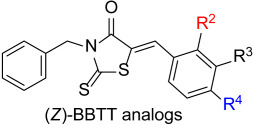	
Substitution	^a^ Tyrosinase Inhibition
R^2^ = OH	**↑↑↑↑** when R^4^ = OH
R^3^ = OH	↓↓ when R^4^ = OMe
	**↑** when R^4^ = OH
R^4^	OH > MeO in the presence of l-tyrosine
	OH ≈ MeO in the presence of l-dopa

^a^ Red and blue arrows represent increases and decreases in tyrosinase inhibitory activity, respectively. More arrows indicate stronger effects.

## Data Availability

The data presented in this study are available in the article and Supplementary Materials.

## Supplementary Materials

The following supporting information can be downloaded at: https://www.mdpi.com/article/10.3390/molecules30030517/s1.
