# Supplementary material for: Design, Synthesis, and Anti-Tyrosinase, Anti-Melanogenic, and Antioxidant Activities of Novel (Z)-3-Benzyl-5-Benzylidene-2-Thioxothiazolidin-4-One Analogs
Source: Molecules. 2025 Jan 23;30(3):517. doi: 10.3390/molecules30030517 (PMC11820736; doi:10.3390/molecules30030517)
Supplement: Supplementary file 1 [file molecules-30-00517-s001.zip › molecules-3421087-supplementary.pdf]

Supporting Information

For

Design, synthesis, and anti-tyrosinase, anti-melanogenic, and antioxidant activities of novel (*Z*)-3-benzyl-5-benzylidene-2-thioxothiazolidin-4-one analogs

Hyeon Seo Park [a,1], Hee Jin Jung [a,1], Hye Soo Park [a], Hye Jin Kim [a], Yujin Park [b], Pusoon Chun [c], Hae Young Chung [d], Hyung Ryong Moon [a,*]

[a]*Department of Manufacturing Pharmacy, College of Pharmacy and Research Institute for Drug Development, Pusan National University, Busan 46241, Republic of Korea*

[b]*Department of Medicinal Chemistry, New Drug Development Center, Daegu-Gyeongbuk Medical Innovation Foundation, Daegu 41061, South Korea*

[c]*College of Pharmacy and Inje Institute of Pharmaceutical Sciences and Research, Inje University, Gimhae 50834, Republic of Korea*

[d]*Department of Pharmacy, College of Pharmacy and Research Institute for Drug Development, Pusan National University, Busan 46241, Republic of Korea*

Contents

S1. ^{1}H NMR spectrum of analog **1** 5
S2. ^{13}C NMR spectrum of analog **1** 6
S3. ^{1}H-coupled ^{13}C NMR spectrum of analog **1** 7
S4. COSY NMR spectrum of analog **1** 8
S5. HSQC NMR spectrum of analog **1** 9
S6. HMBC NMR spectrum of analog **1** 10
S7. LRMS (ESI–) spectrum of analog **1** 11
S8-1. HRMS (ESI+) spectrum of analog **1** 12
S8-2. HRMS (ESI+) spectrum of analog **1** 13
S9. ^{1}H NMR spectrum of analog **2** 14
S10. ^{13}C NMR spectrum of analog **2** 15
S11. ^{1}H-coupled ^{13}C NMR spectrum of analog **2** 16
S12. COSY NMR spectrum of analog **2** 17
S13. HSQC NMR spectrum of analog **2** 18
S14. HMBC NMR spectrum of analog **2** 19
S15. LRMS (ESI–) spectrum of analog **2** 20
S16-1. HRMS (ESI+) spectrum of analog **2** 21
S16-2. HRMS (ESI+) spectrum of analog **2** 22
S17. ^{1}H NMR spectrum of analog **3** 23
S18. ^{13}C NMR spectrum of analog **3** 24
S19. ^{1}H-coupled ^{13}C NMR spectrum of analog **2** 25
S20. LRMS (ESI–) spectrum of analog **3** 26
S21-1. HRMS (ESI+) spectrum of analog **3** 27

S21-2. HRMS (ESI+) spectrum of analog **3** 28

S22. ^{1}H NMR spectrum of analog **4** 29

S23. ^{13}C NMR spectrum of analog **4** 30

S24. LRMS (ESI–) spectrum of analog **4** 31

S25. ^{1}H NMR spectrum of analog **5** 32

S26. ^{13}C NMR spectrum of analog **5** 33

S27. LRMS (ESI–) spectrum of analog **5** 34

S28. ^{1}H NMR spectrum of analog **6** 35

S29. ^{13}C NMR spectrum of analog **6** 36

S30-1. HRMS (ESI+) spectrum of analog **6** 37

S30-2. HRMS (ESI+) spectrum of analog **6** 38

S31. ^{1}H NMR spectrum of analog **7** 39

S32. ^{13}C NMR spectrum of analog **7** 40

S33. LRMS (ESI+) spectrum of analog **7** 41

S34. ^{1}H NMR spectrum of analog **8** 42

S35. ^{13}C NMR spectrum of analog **8** 43

S36. LRMS (ESI+) spectrum of analog **8** 44

S37. ^{1}H NMR spectrum of analog **9** 45

S38. ^{13}C NMR spectrum of analog **9** 46

S39. LRMS (ESI+) spectrum of analog **9** 47

S40. ^{1}H NMR spectrum of analog **10** 48

S41. ^{13}C NMR spectrum of analog **10** 49

S42. LRMS (ESI+) spectrum of analog **10** 50

S43. LRMS (ESI–) spectrum of analog **10** 51

S44. ^{1}H NMR spectrum of analog **11** 52

S45. ^{13}C NMR spectrum of analog **11** 53

S46. LRMS (ESI–) spectrum of analog **11** 54

S47. ^{1}H NMR spectrum of analog **12** 55

S48. ^{13}C NMR spectrum of analog **12** 56

S49. LRMS (ESI–) spectrum of analog **12** 57

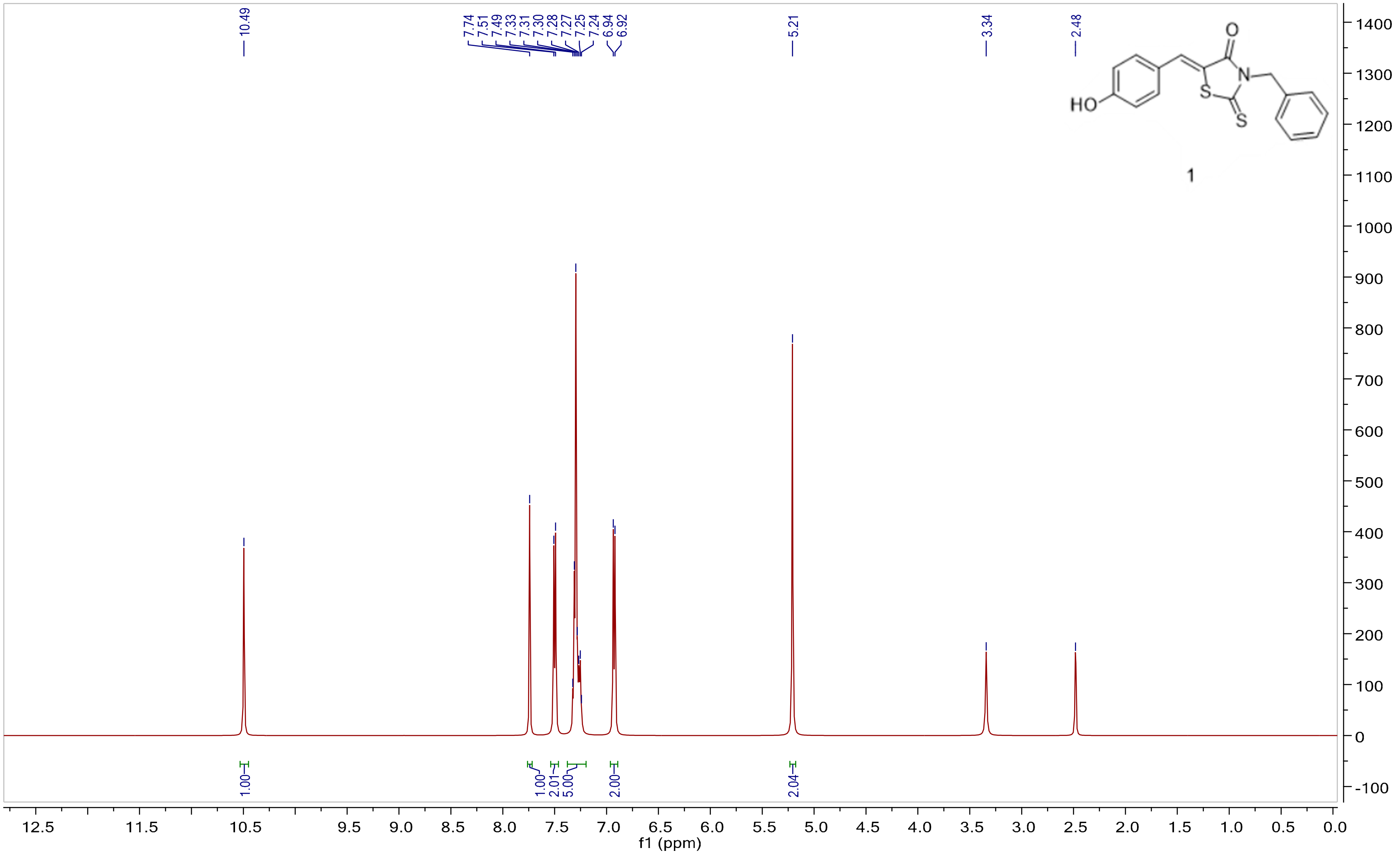

S1. ^{1}H NMR spectrum of analog **1**

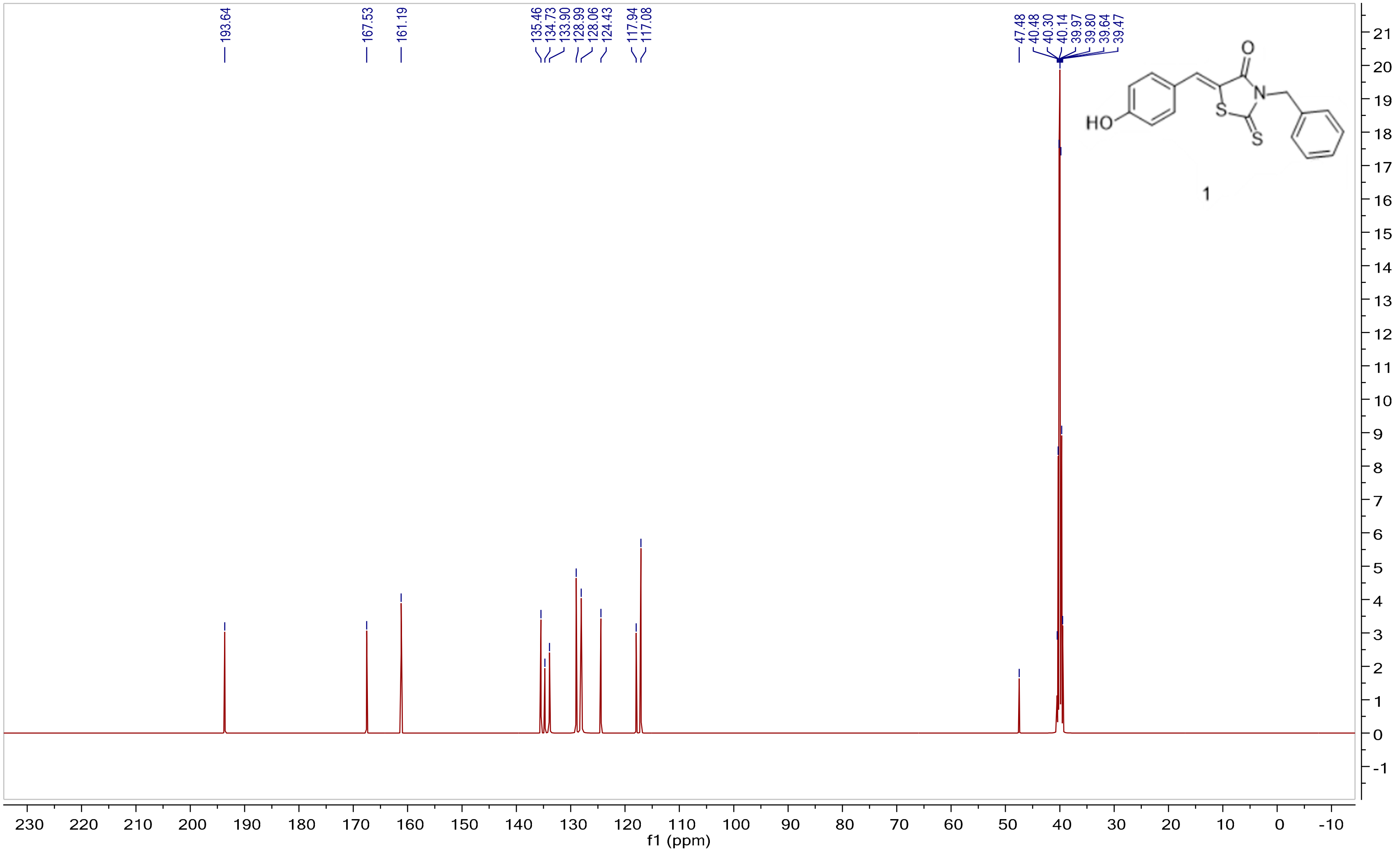

S2. ^{13}C NMR spectrum of analog **1**

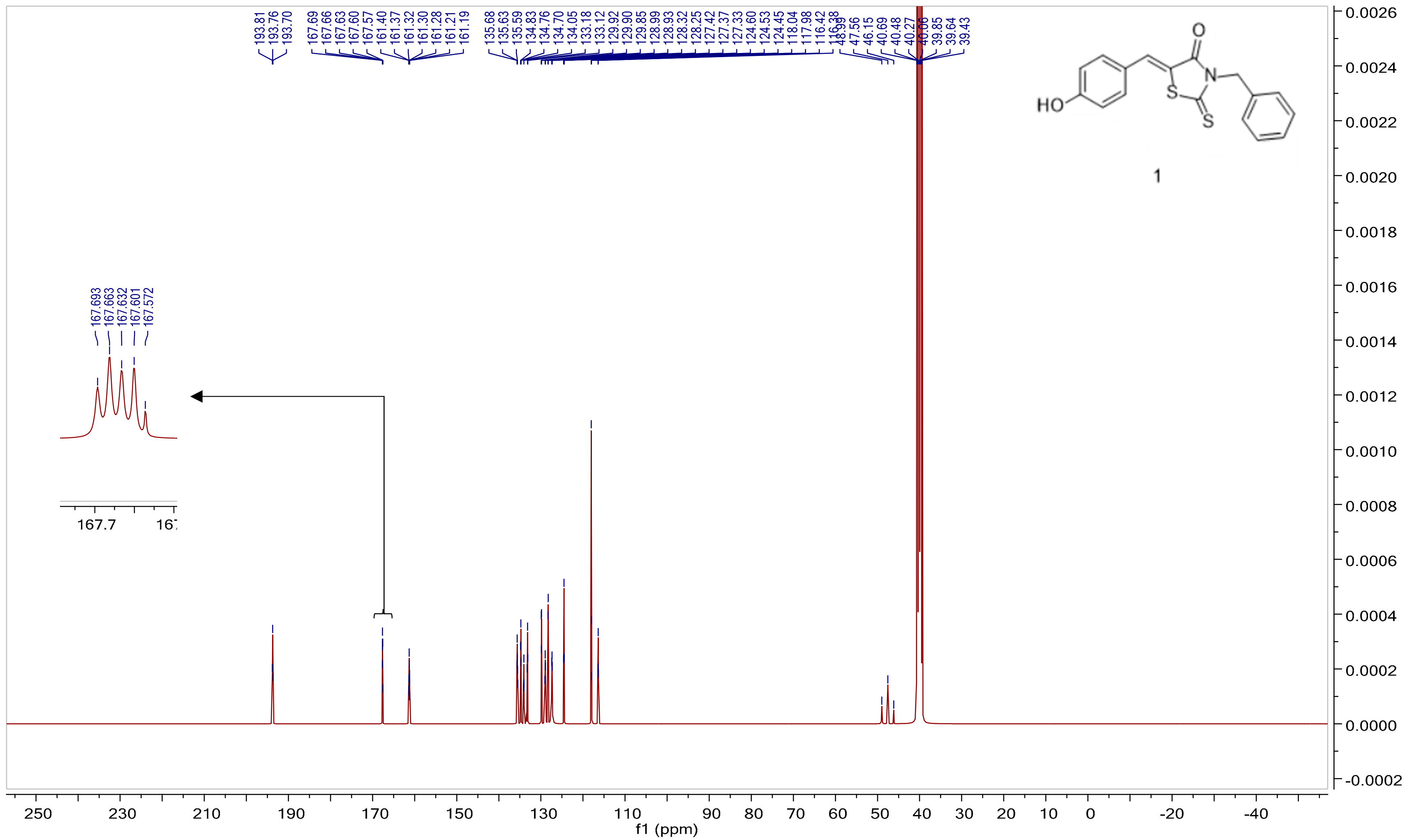

S3. ^{1}H-coupled ^{13}C NMR spectrum of analog **1**

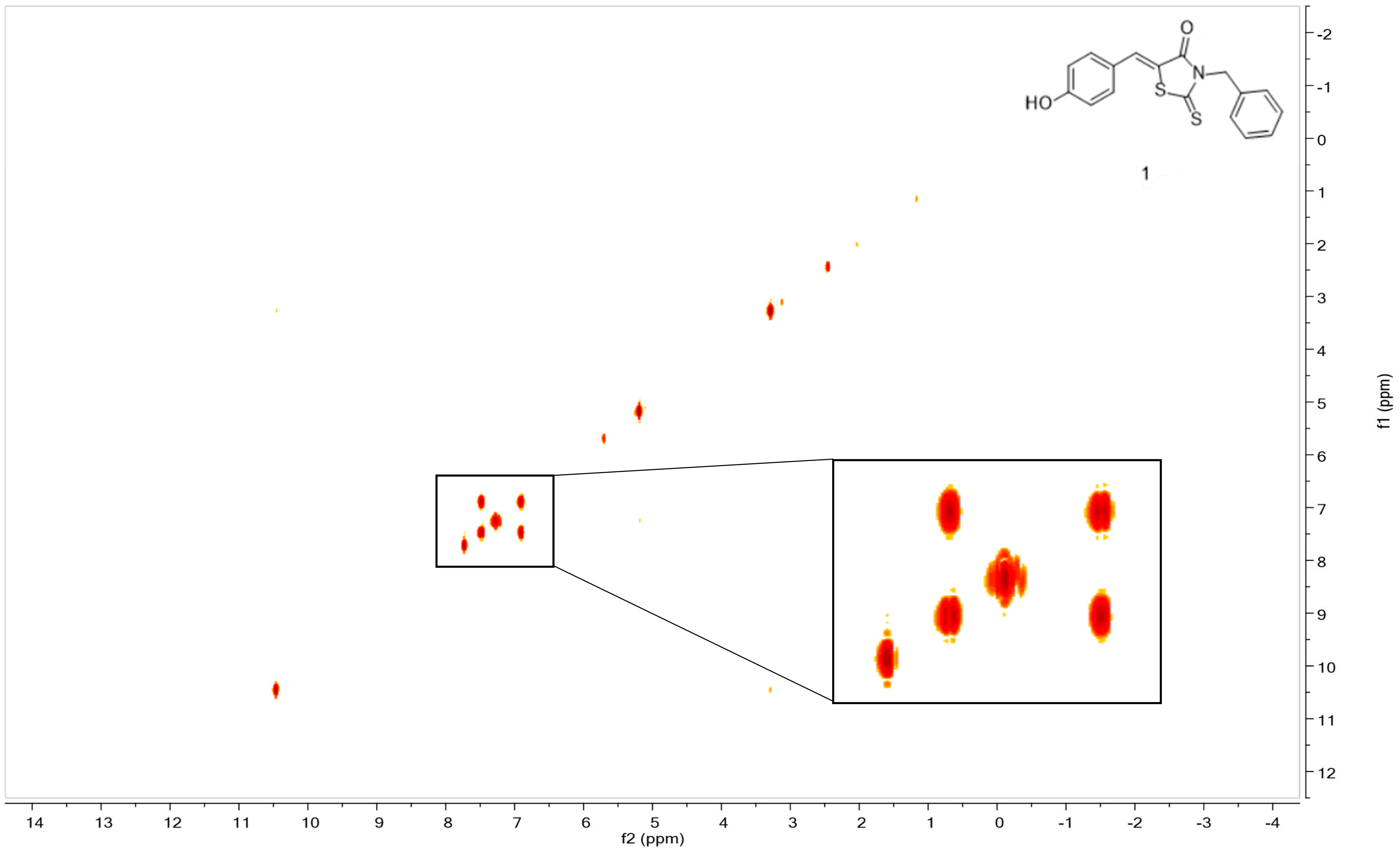

S4. COSY NMR spectrum of analog **1**

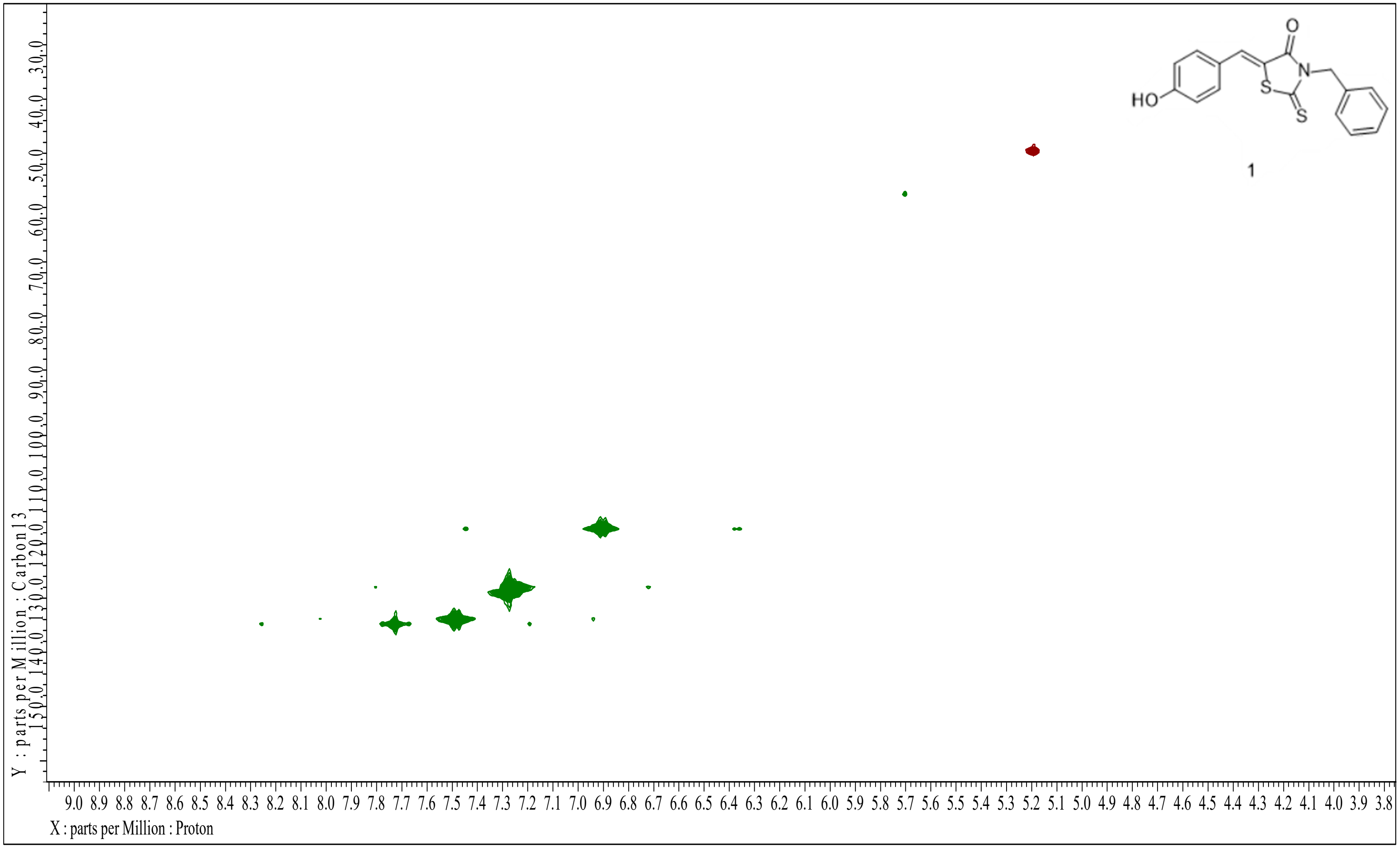

S5. HSQC NMR spectrum of analog **1**

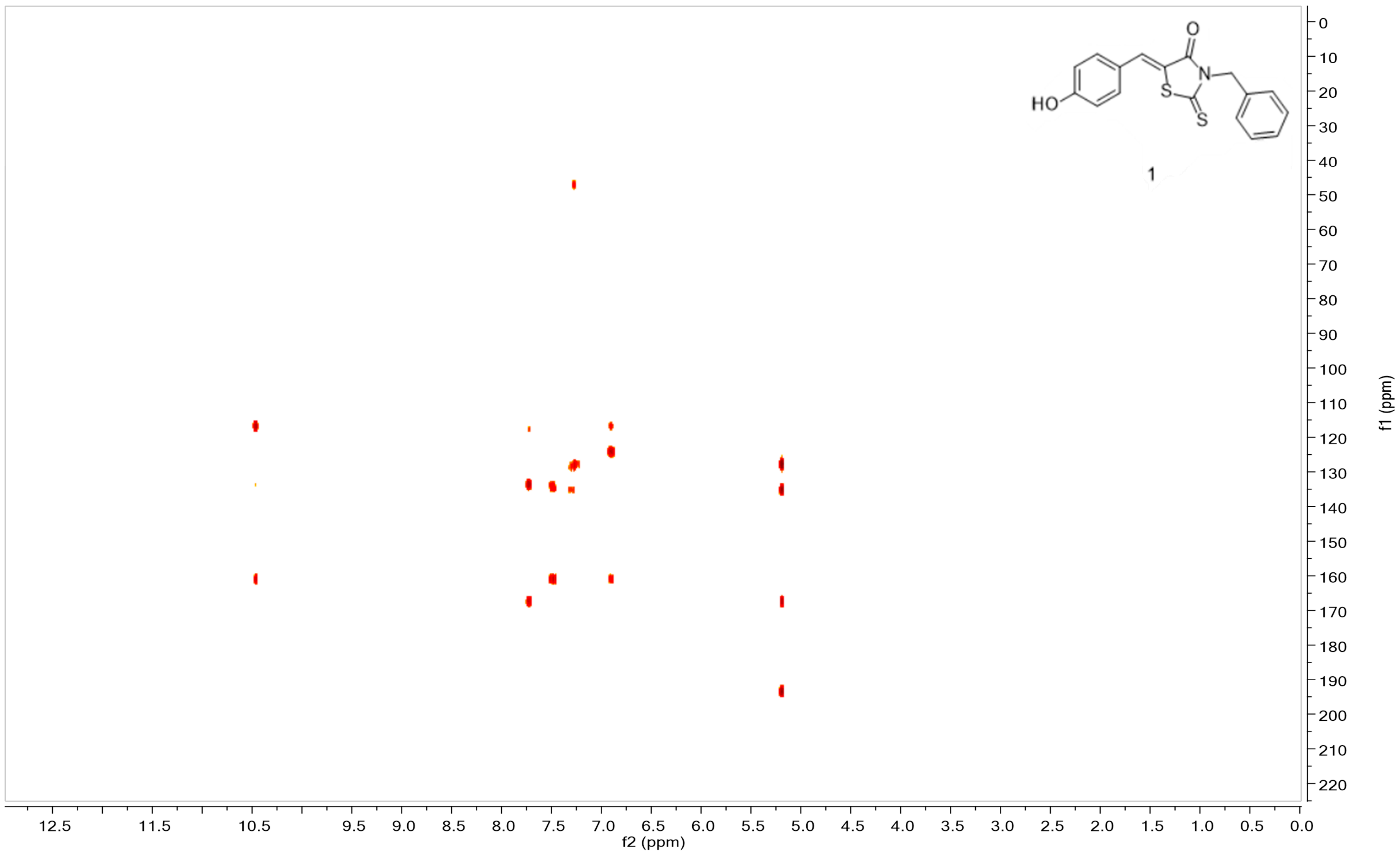

S6. HMBC NMR spectrum of analog **1**

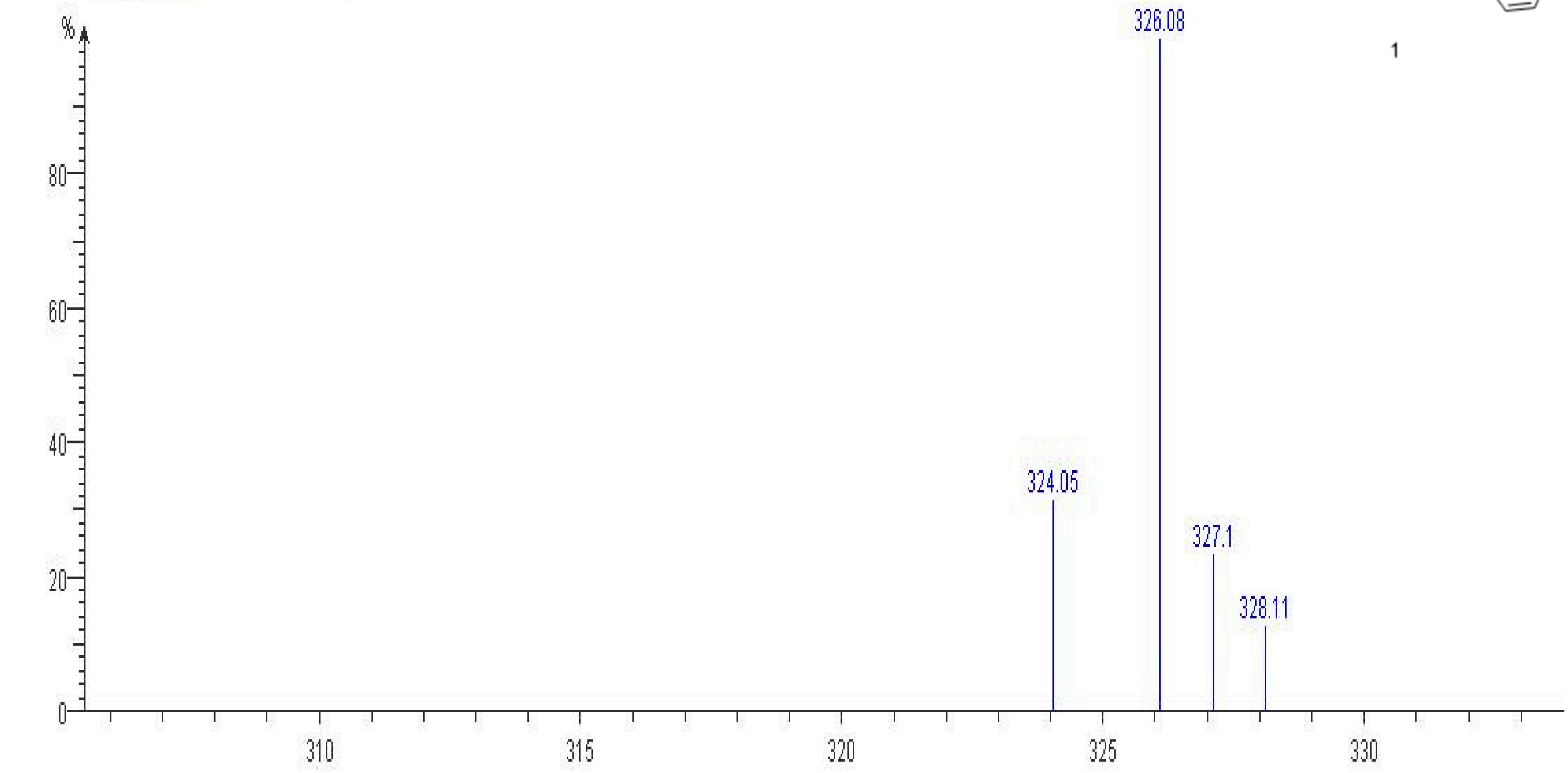

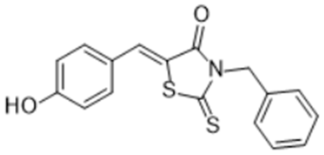

1

S7. LRMS (ESI–) spectrum of analog **1**

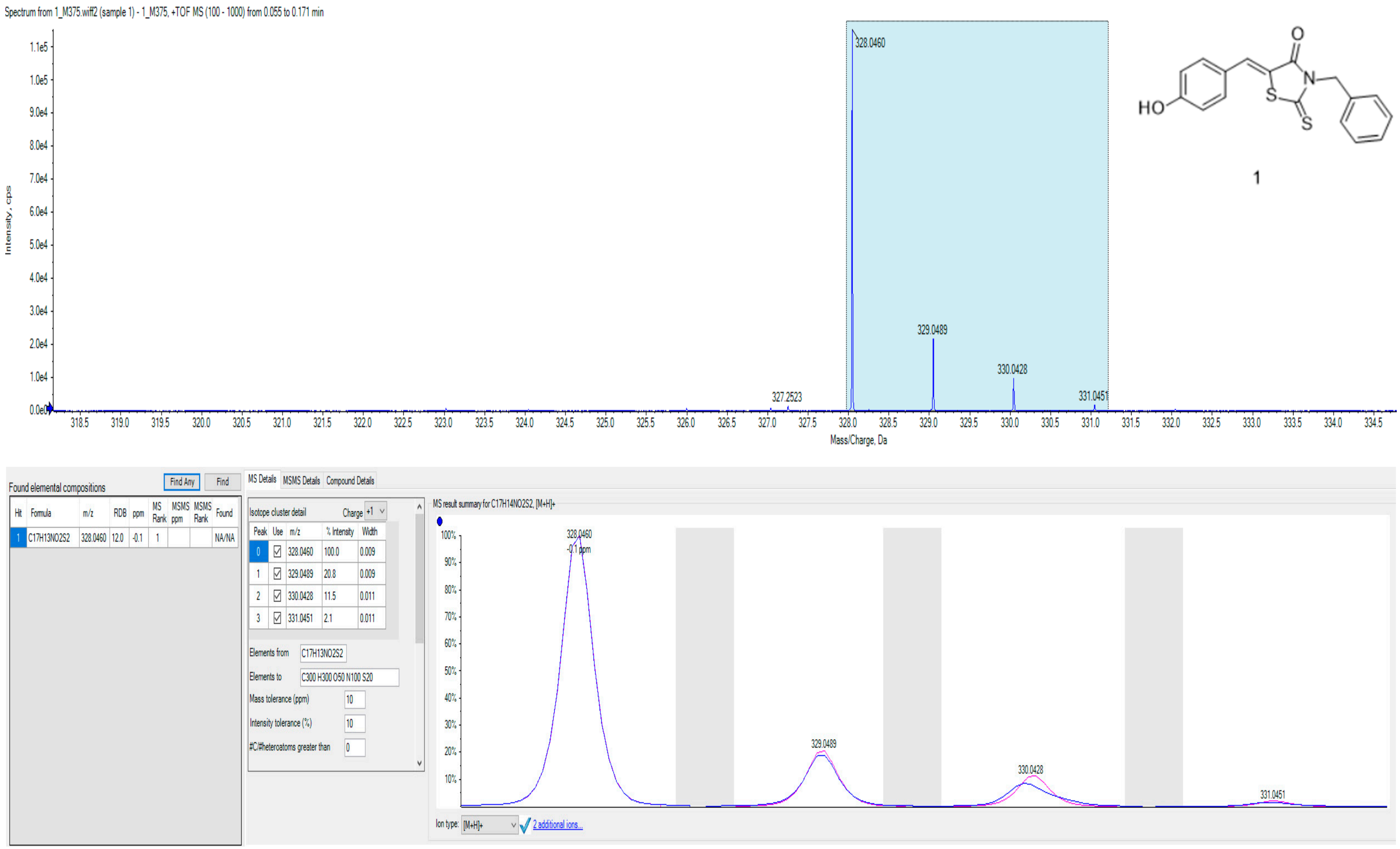

S8-1. HRMS (ESI+) spectrum of analog **1**

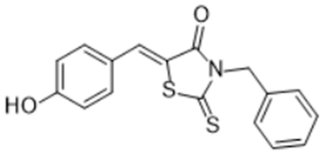

1

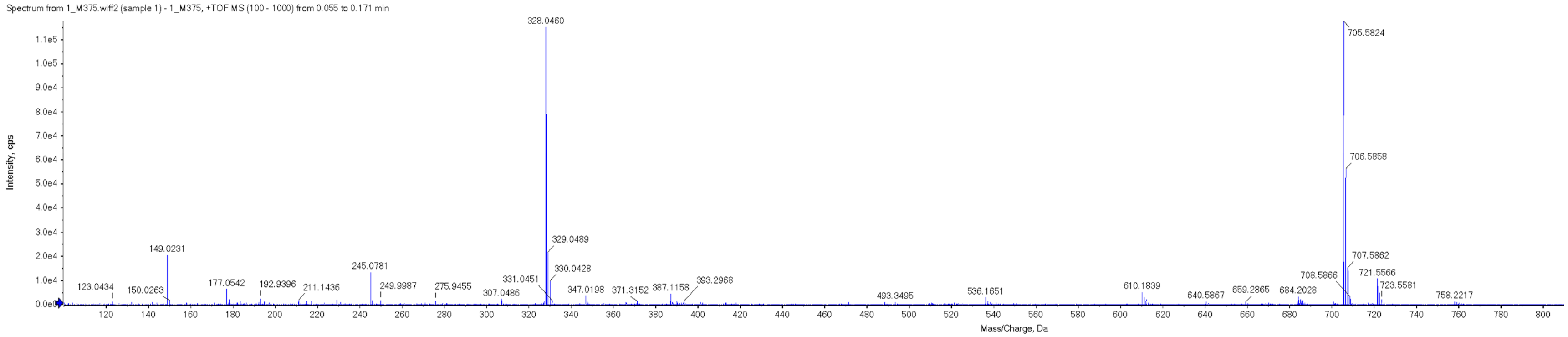

S8-2. HRMS (ESI+) spectrum of analog **1**

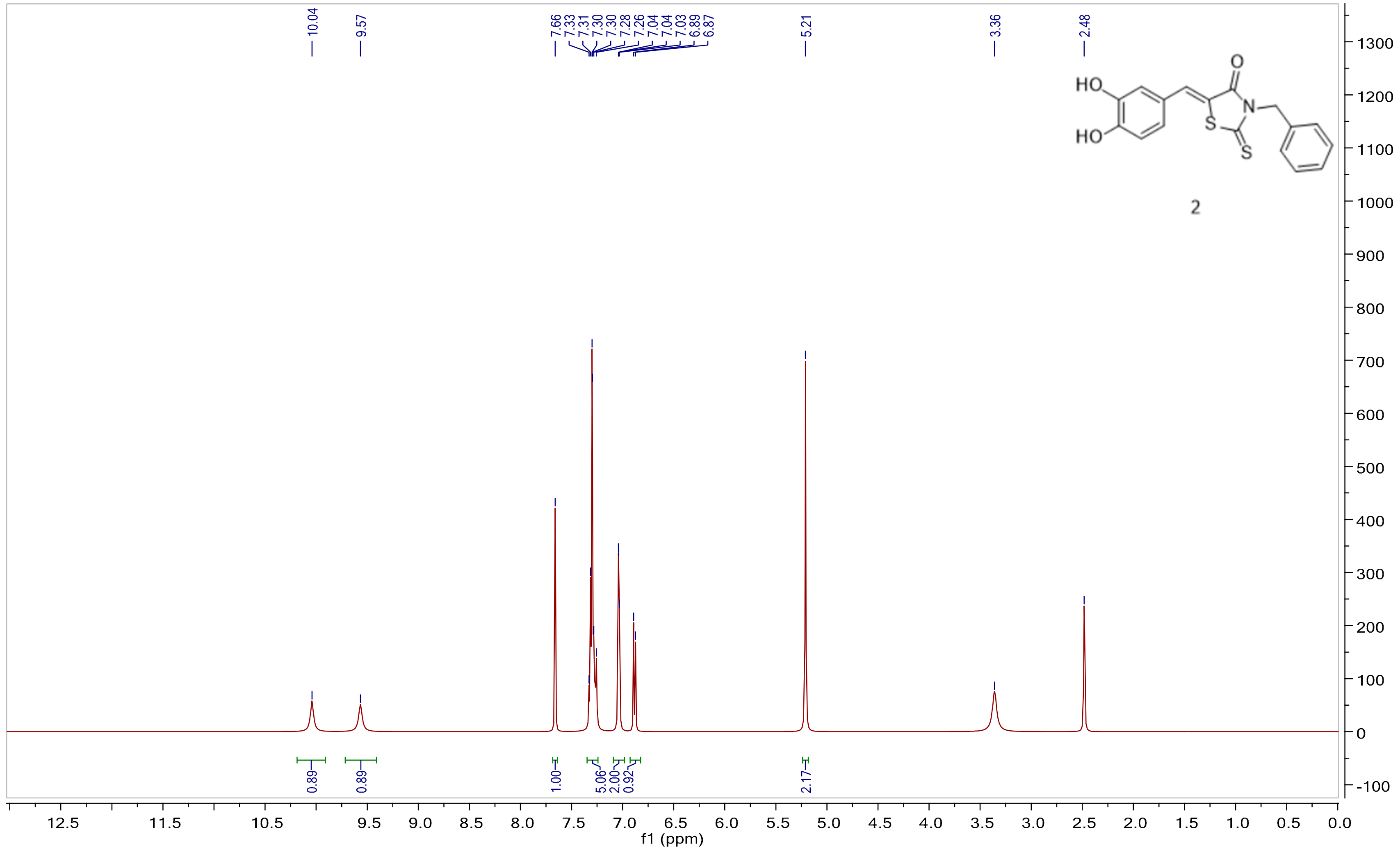

S9. ^{1}H NMR spectrum of analog **2**

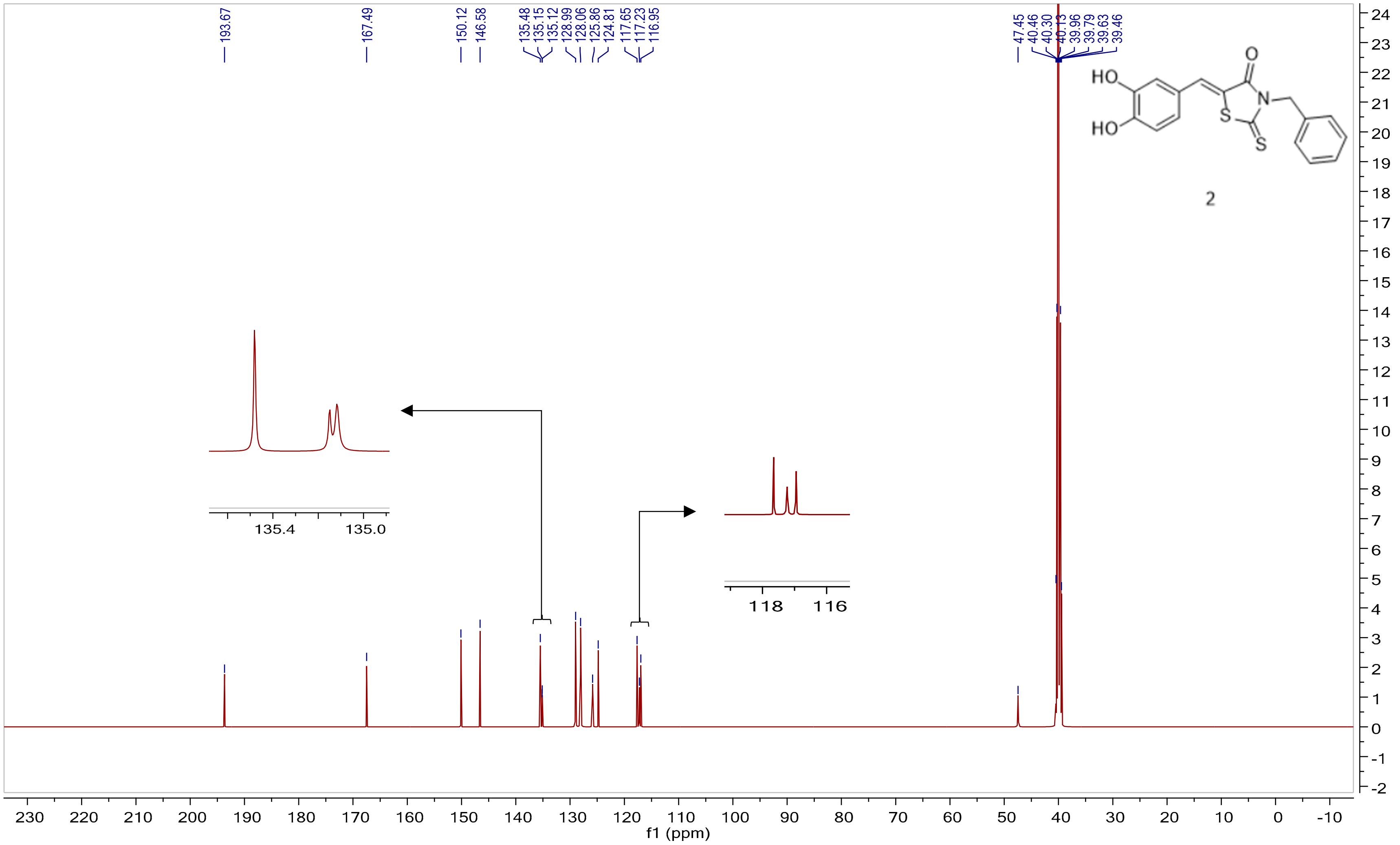

S10. ^{13}C NMR spectrum of analog **2**

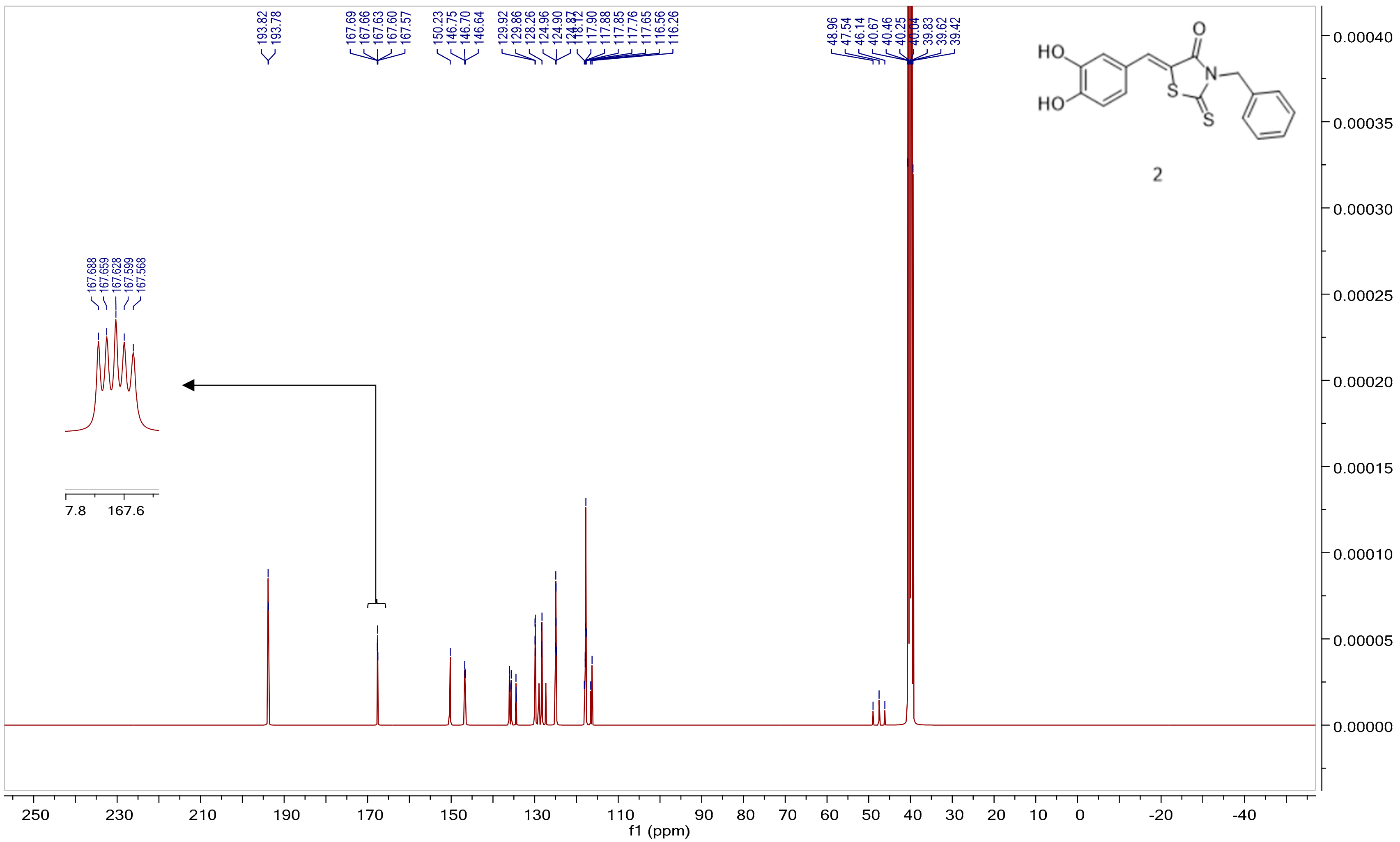

S11. ^{1}H-coupled ^{13}C NMR spectrum of analog **2**

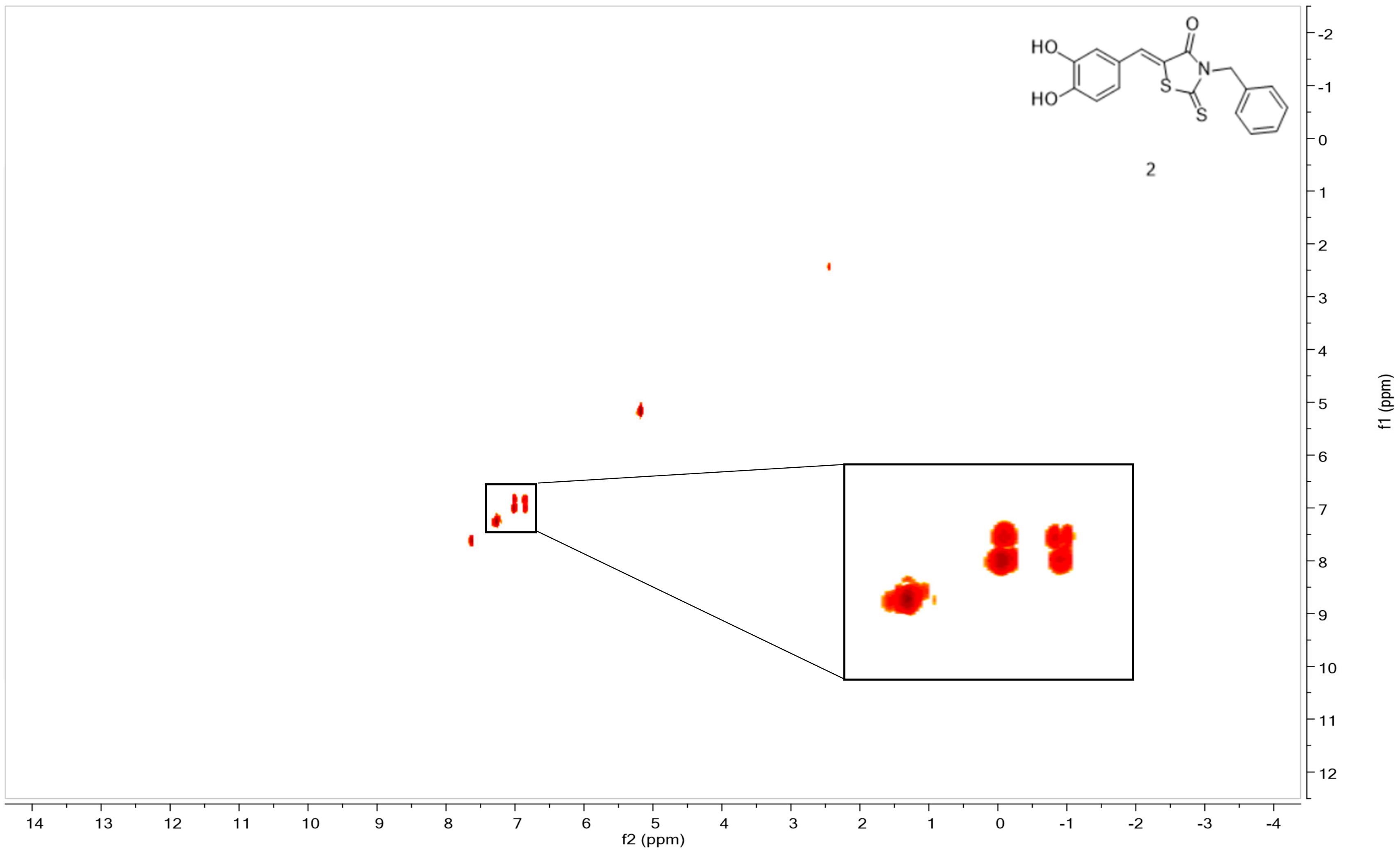

S12. COSY NMR spectrum of analog **2**

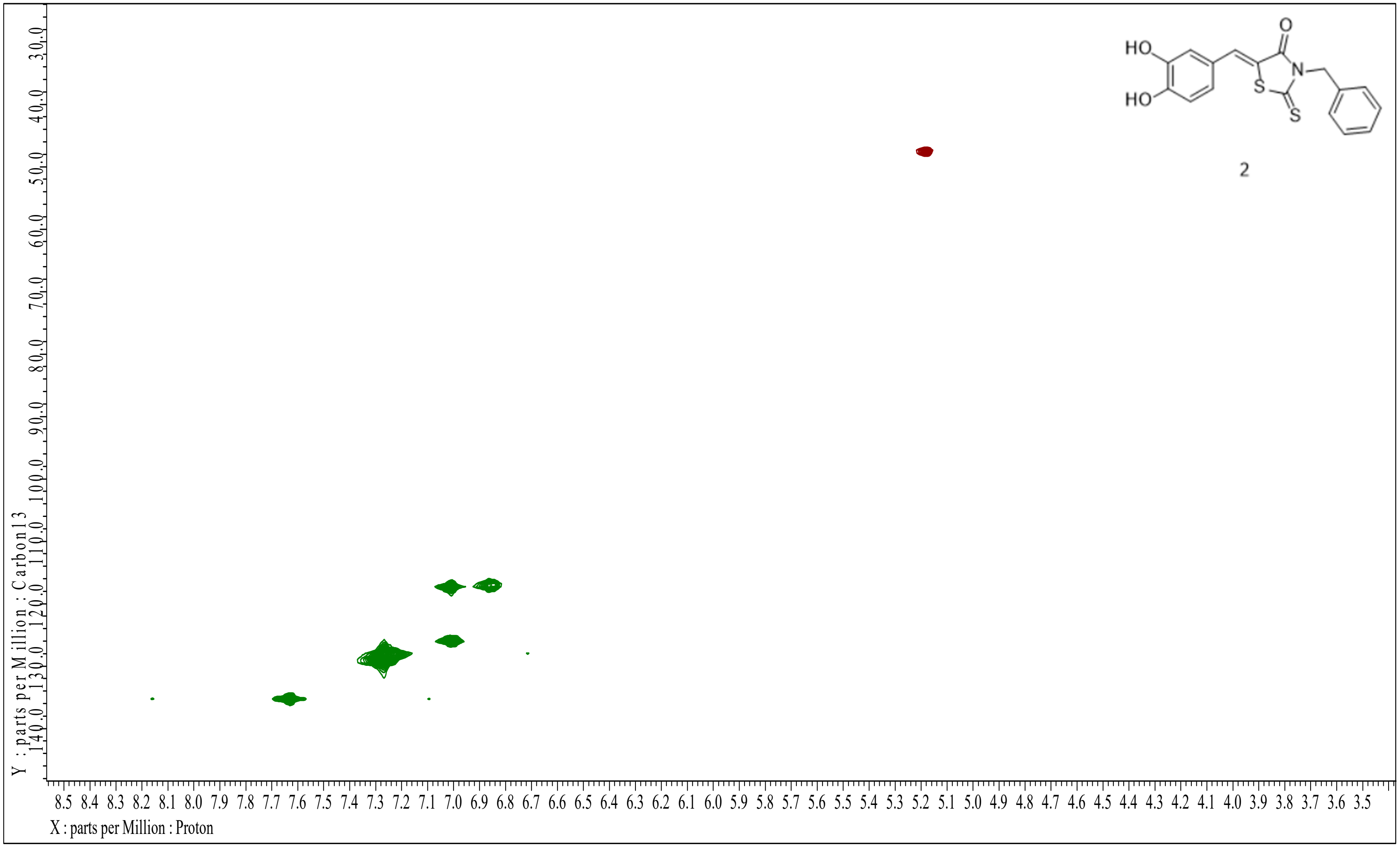

S13. HSQC NMR spectrum of analog **2**

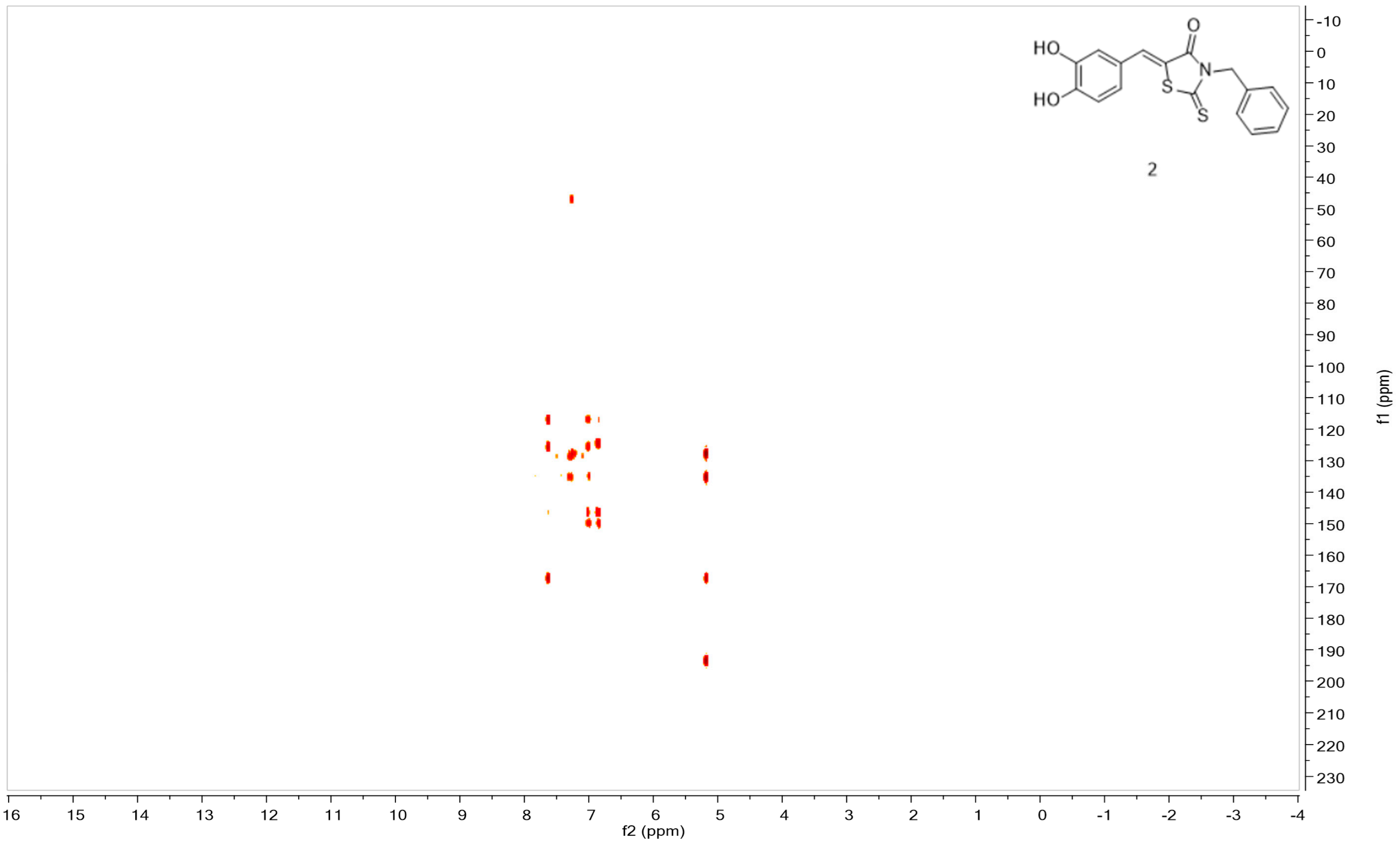

S14. HMBC NMR spectrum of analog **2**

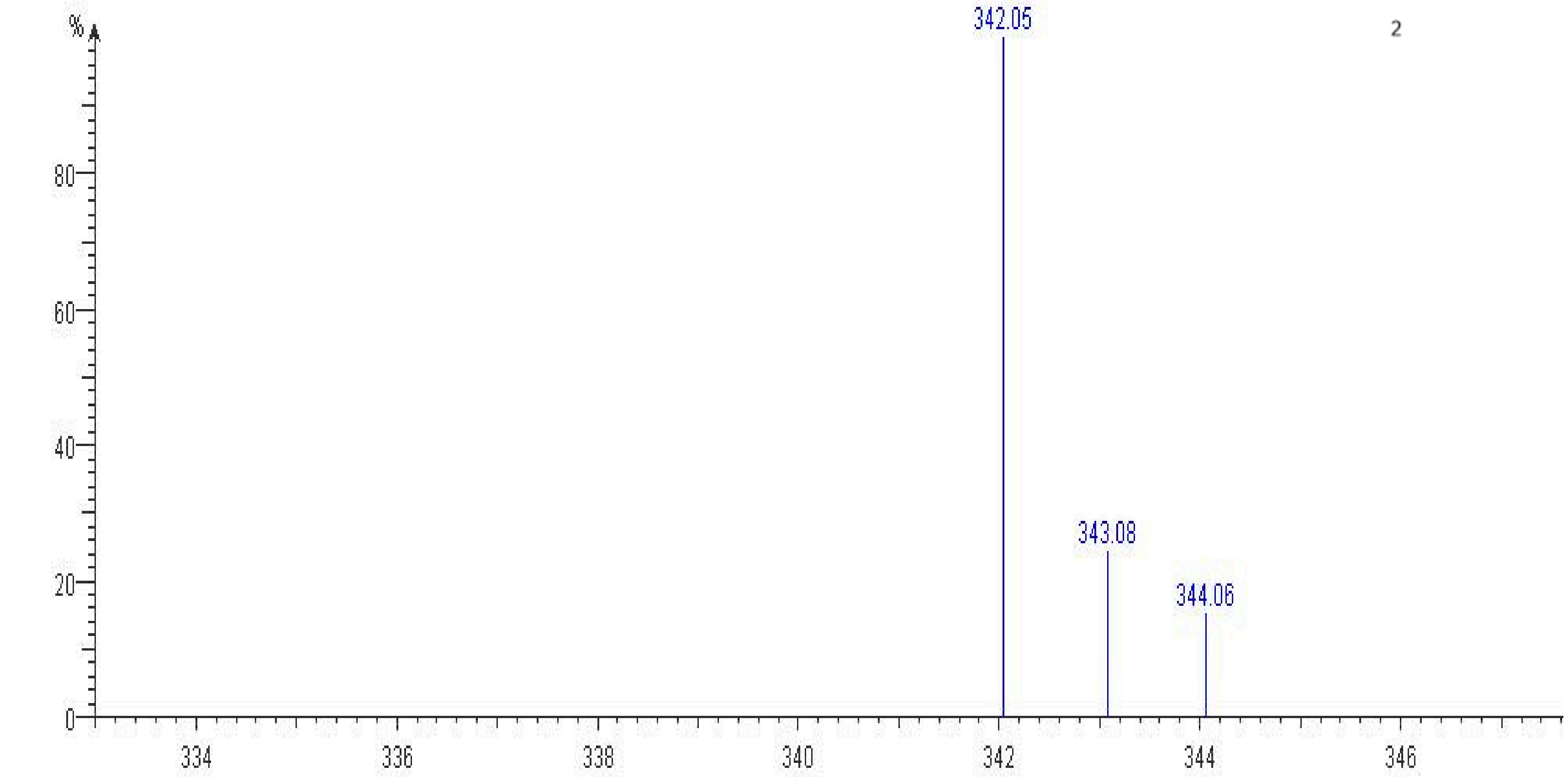

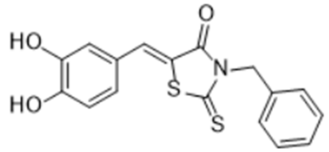

2

S15. LRMS (ESI–) spectrum of analog **2**

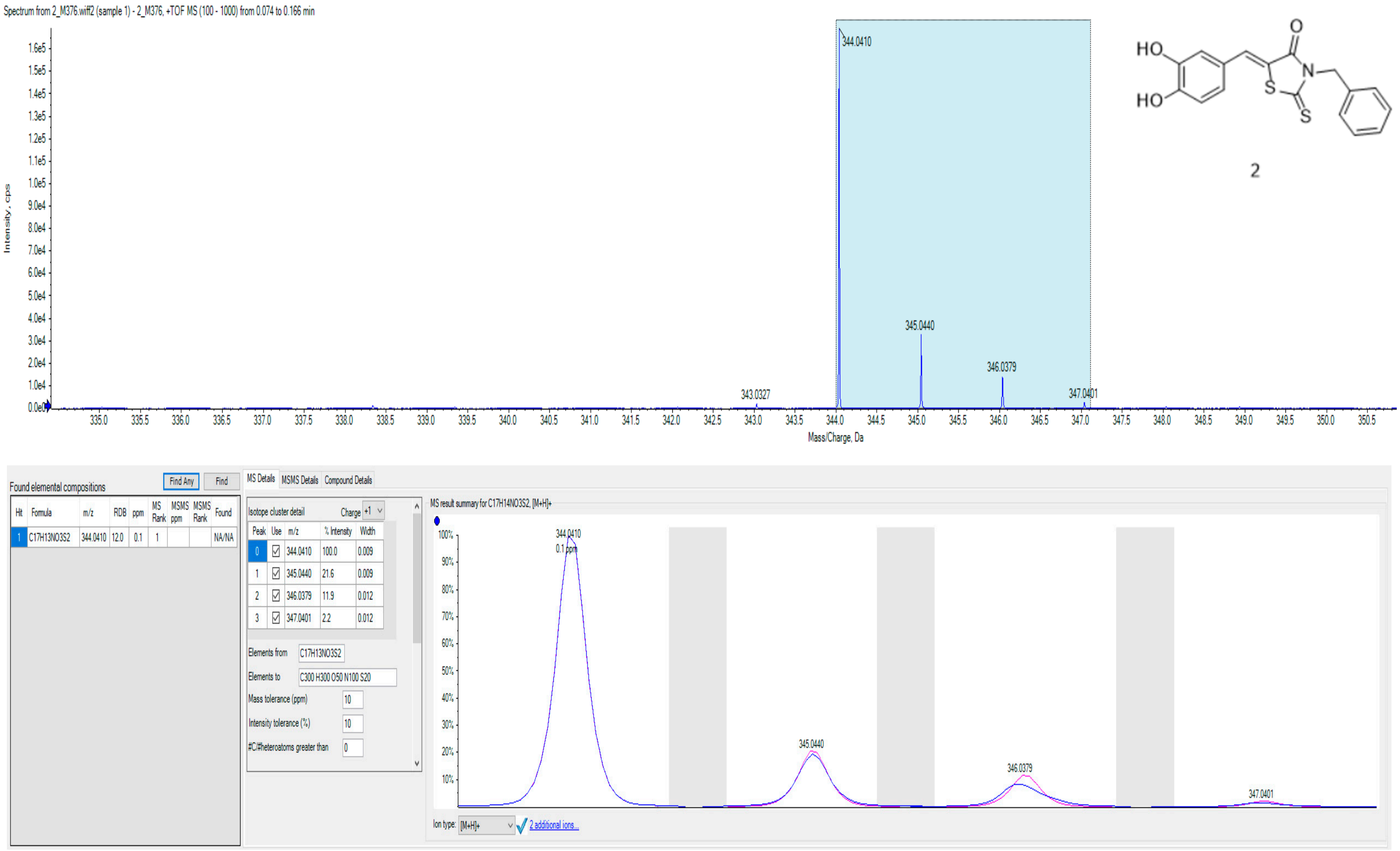

S16-1. HRMS (ESI+) spectrum of analog **2**

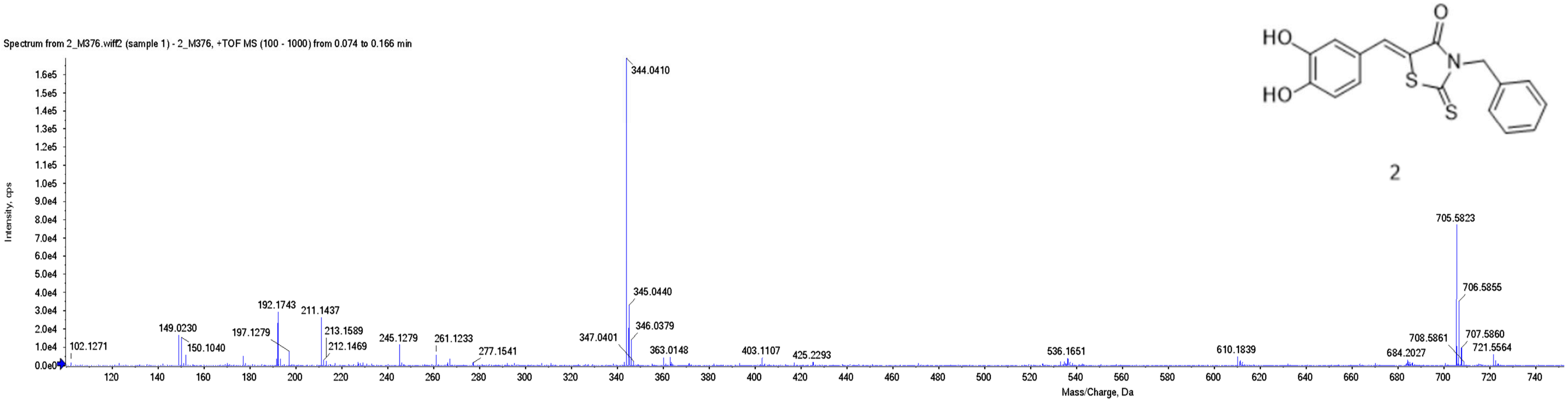

S16-2. HRMS (ESI+) spectrum of analog **2**

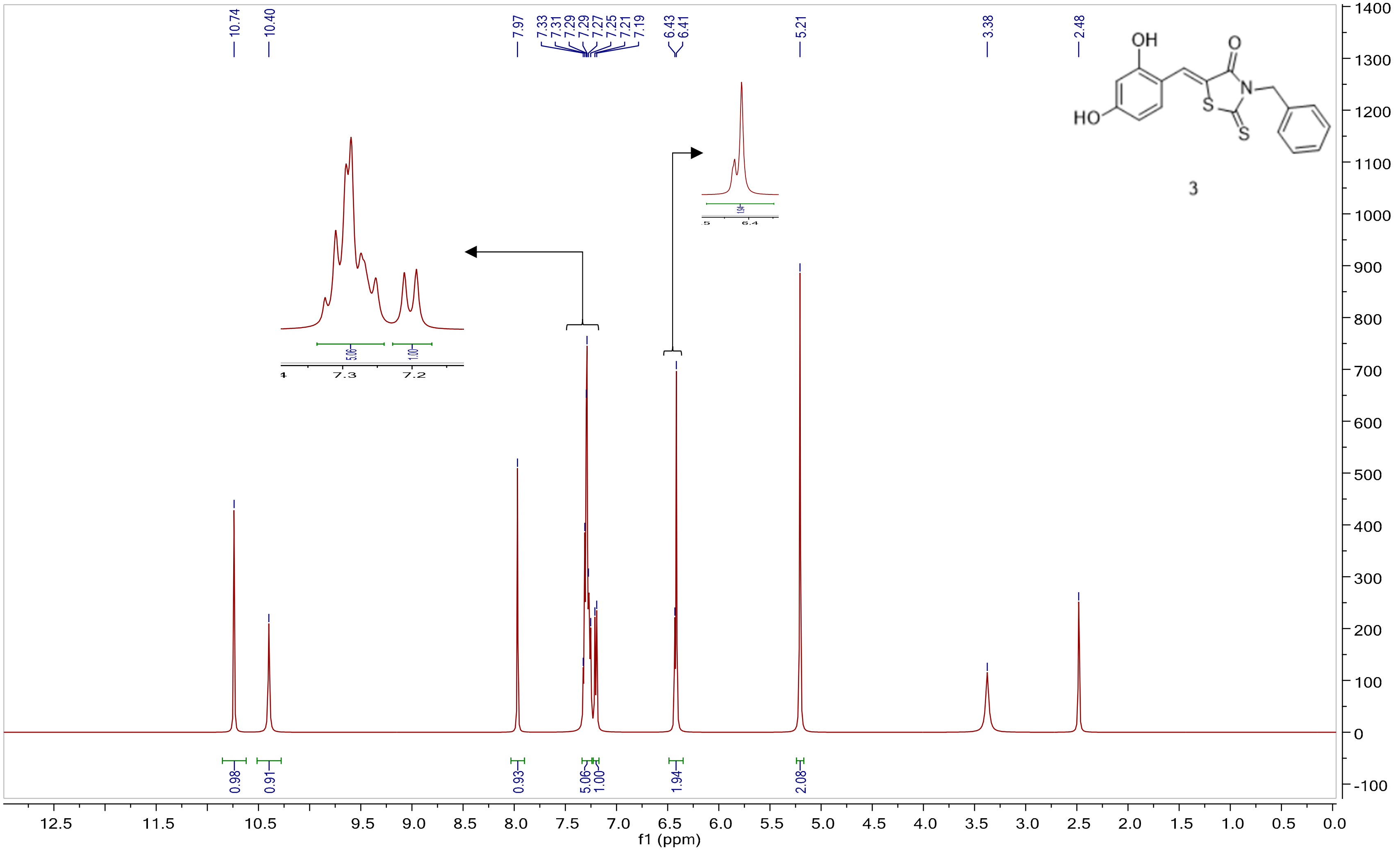

S17. ^{1}H NMR spectrum of analog **3**

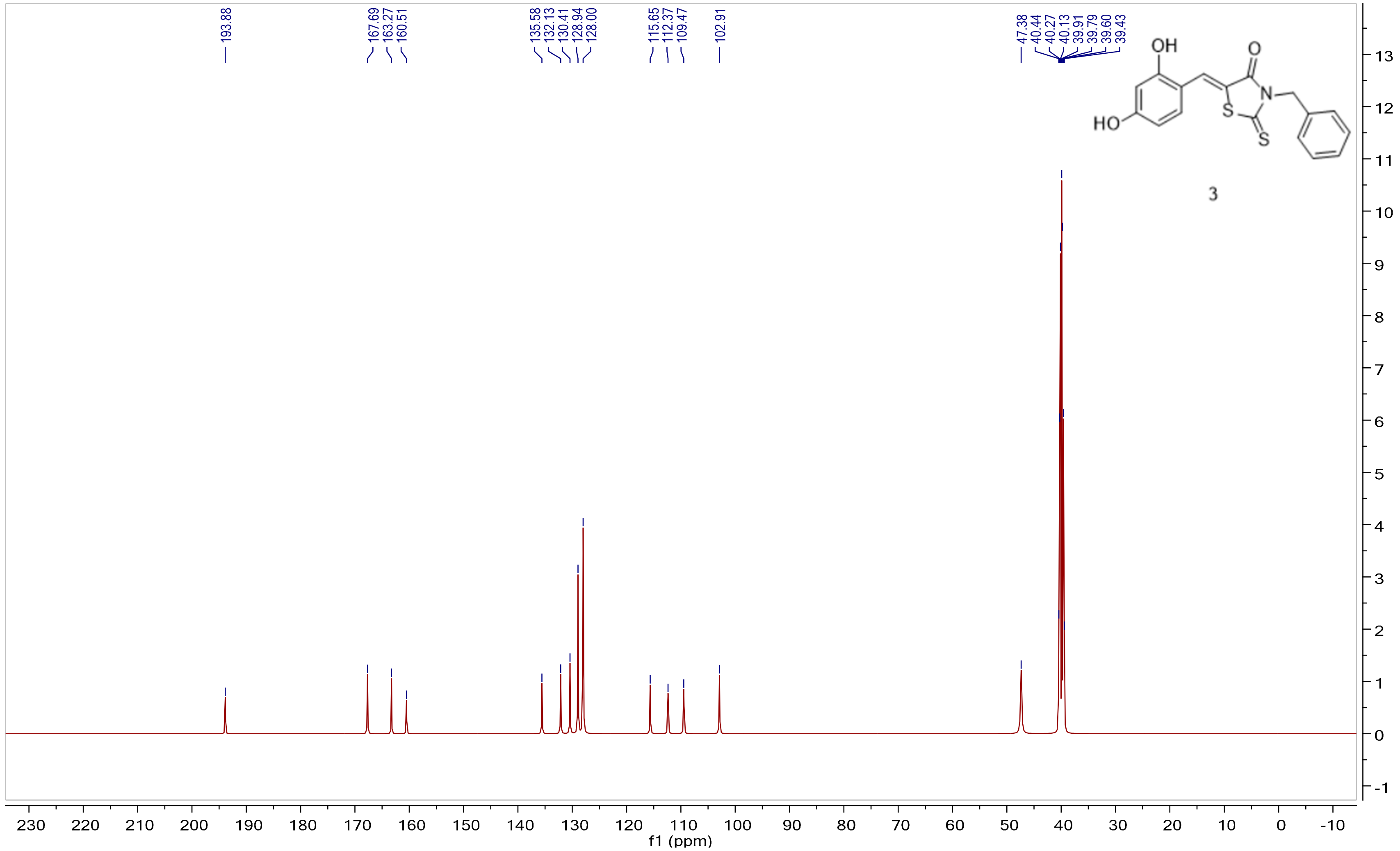

S18. ^{13}C NMR spectrum of analog **3**

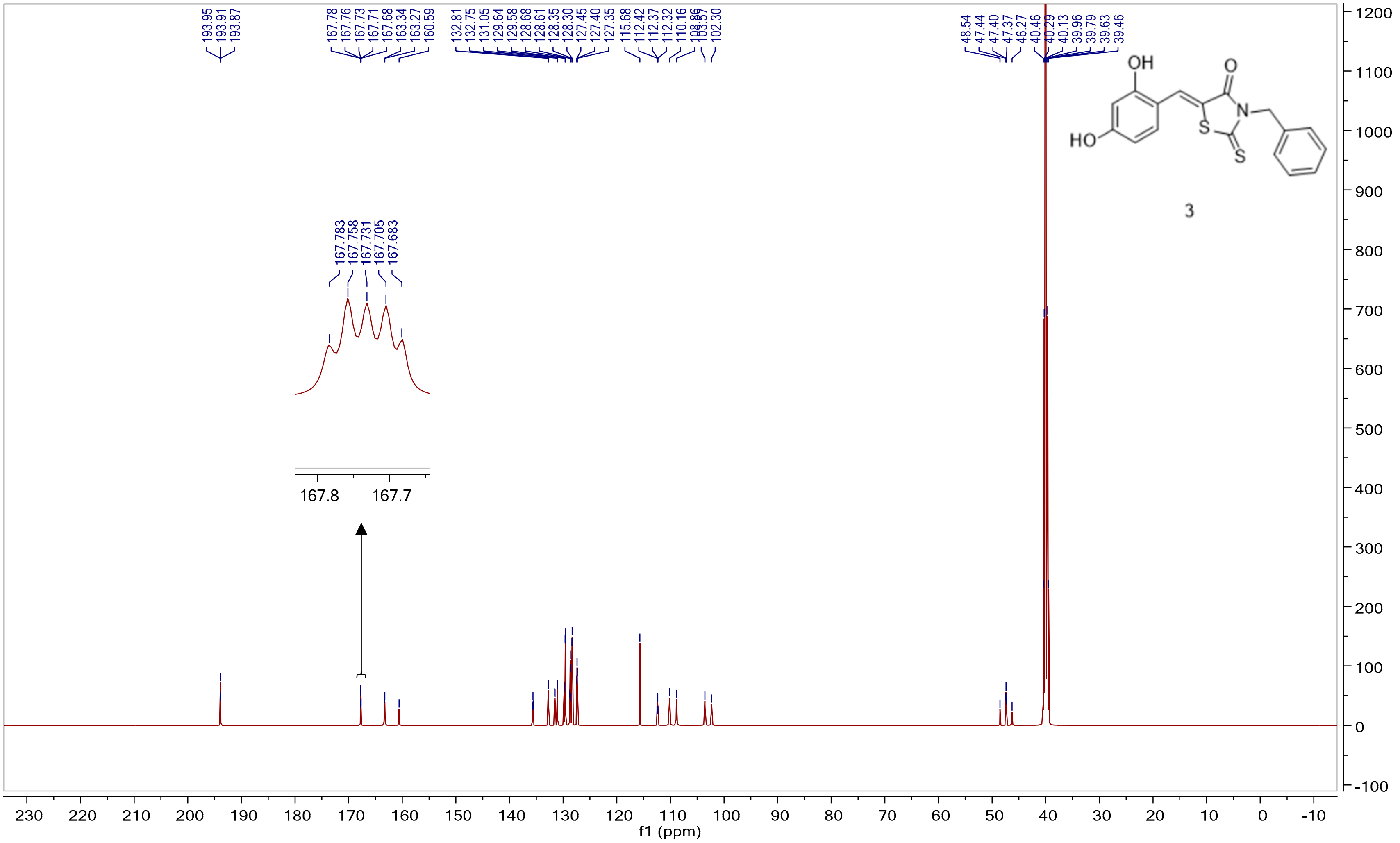

S19. ^{1}H-coupled ^{13}C NMR spectrum of analog **3**

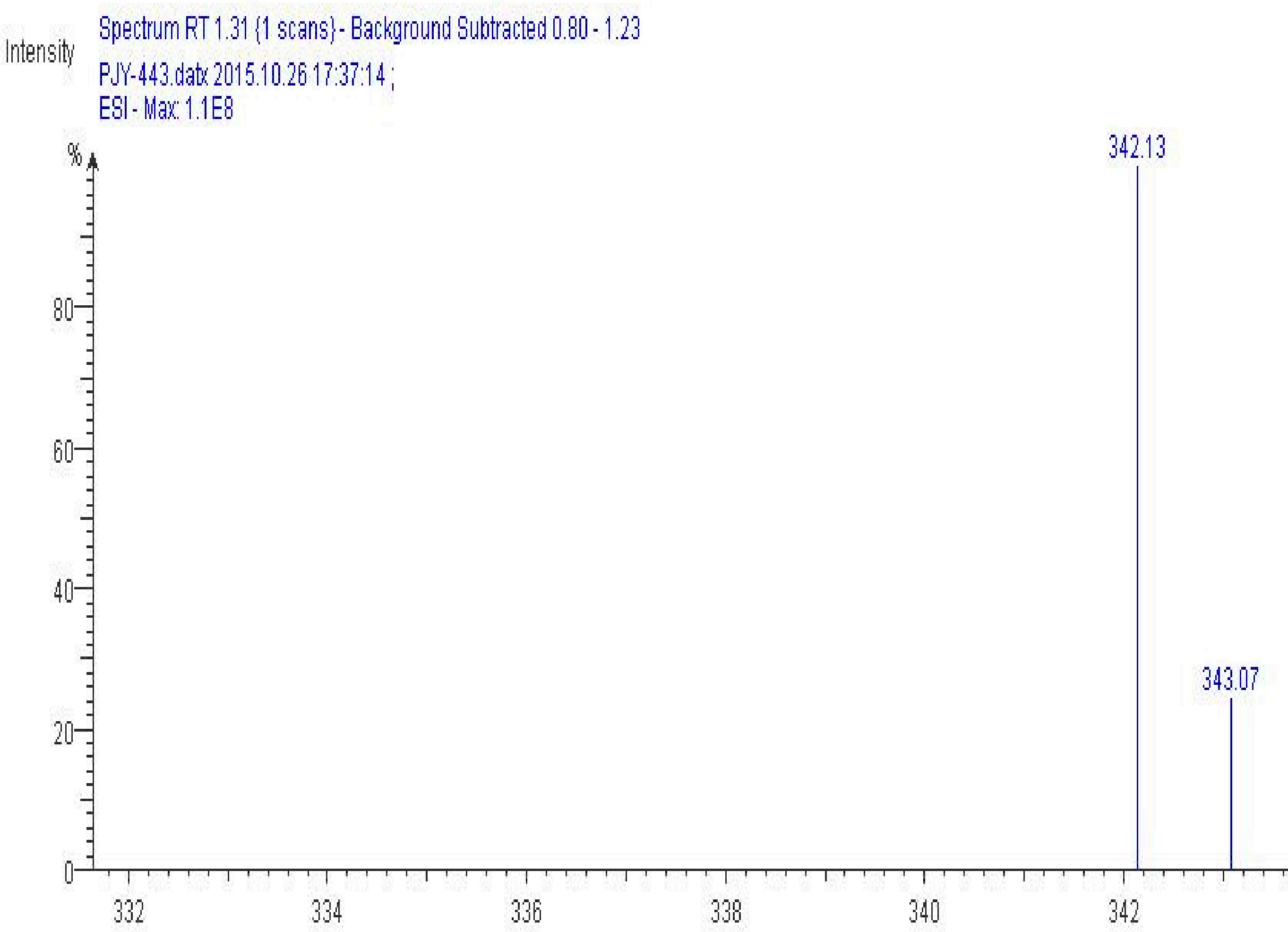

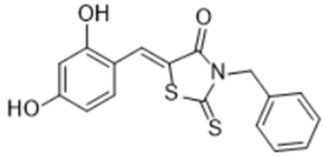

3

S20. LRMS (ESI–) spectrum of analog **3**

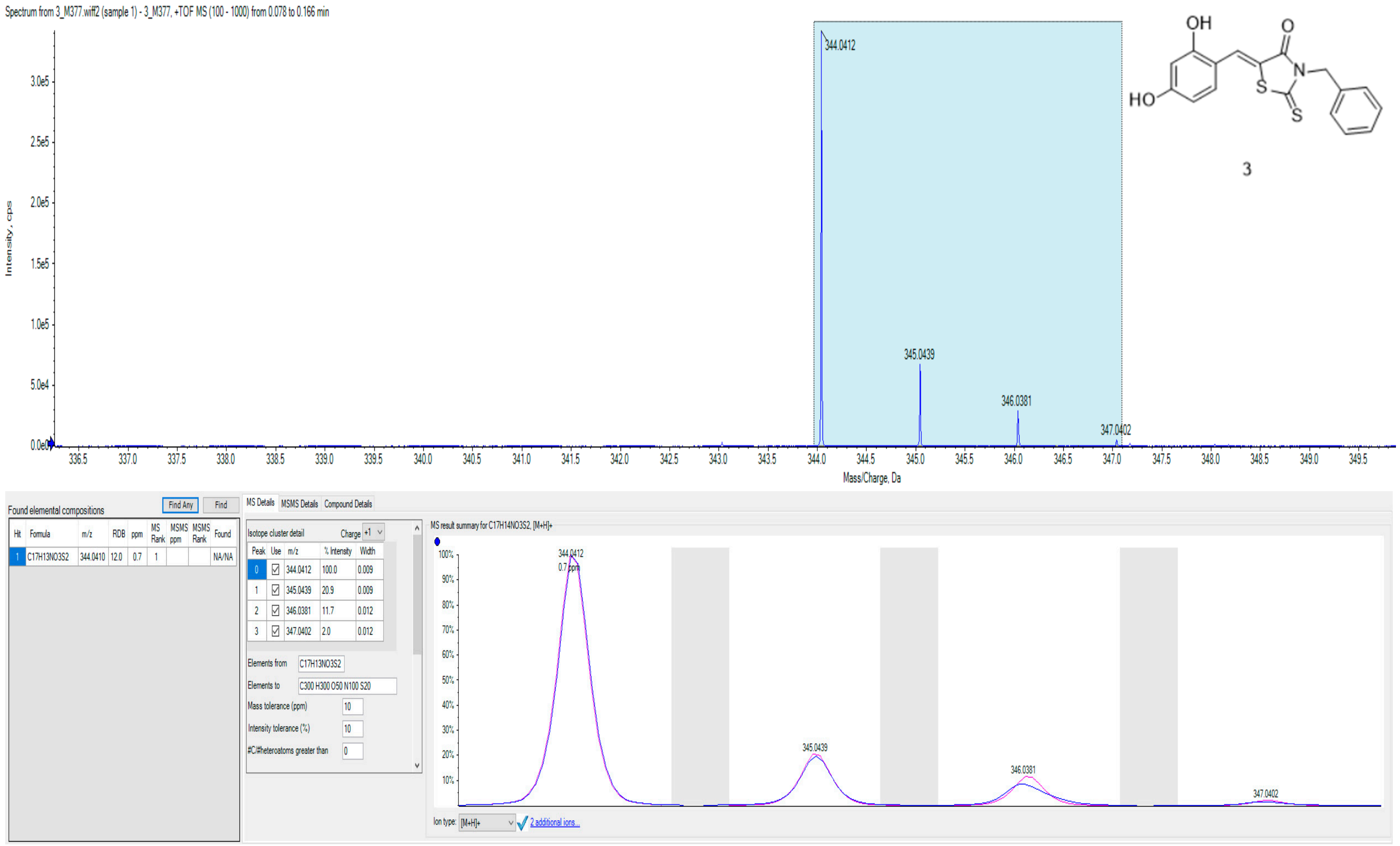

S21-1. HRMS (ESI+) spectrum of analog **3**

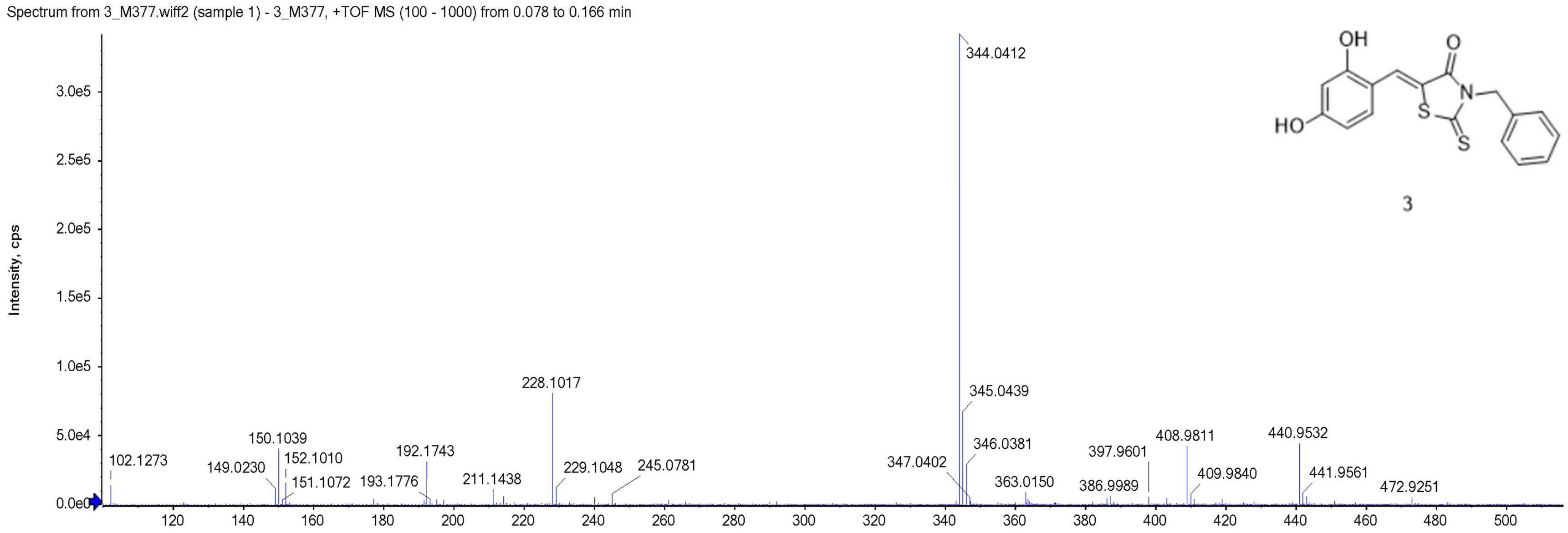

S21-2. HRMS (ESI+) spectrum of analog **3**

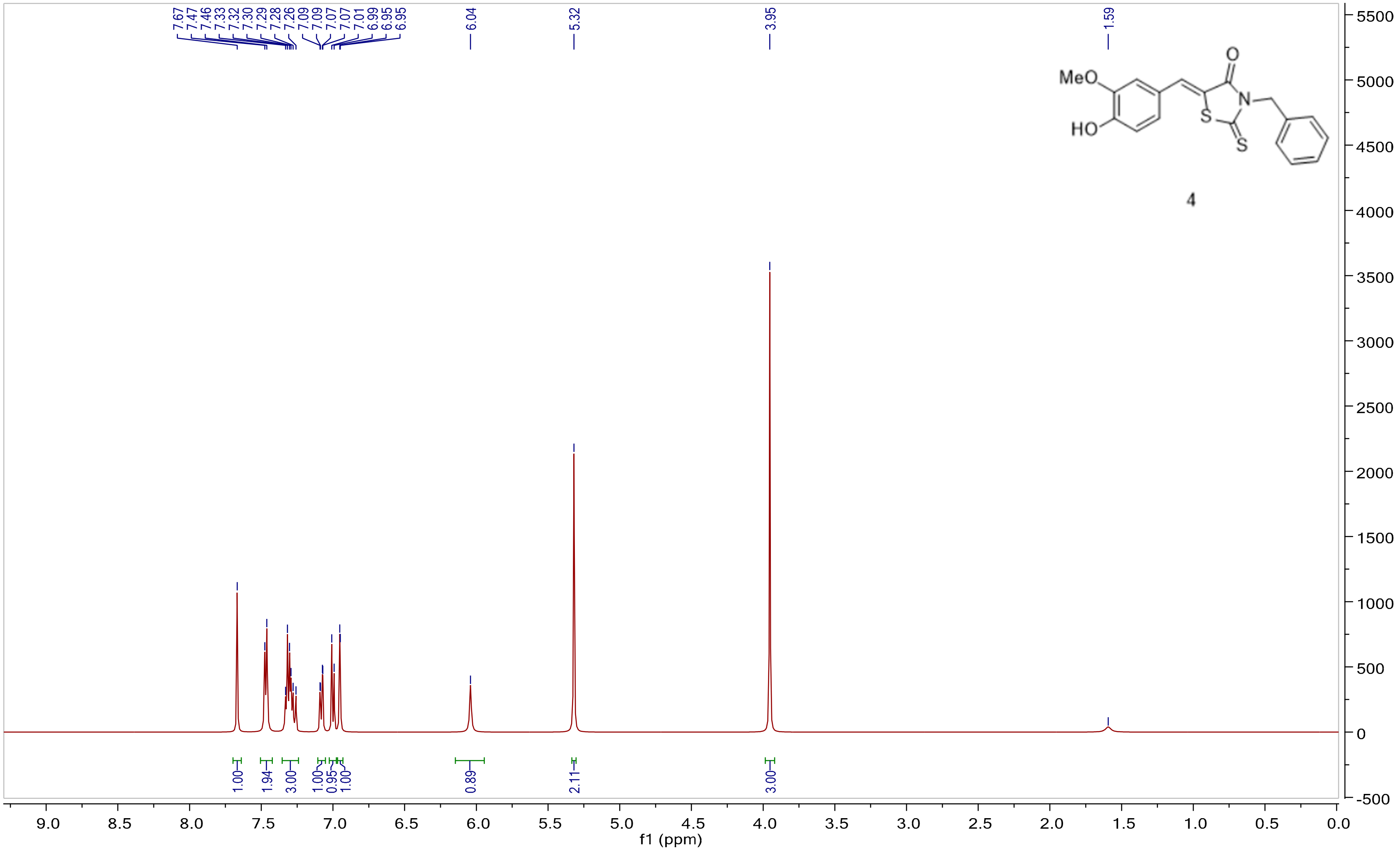

S22. ^{1}H NMR spectrum of analog **4**

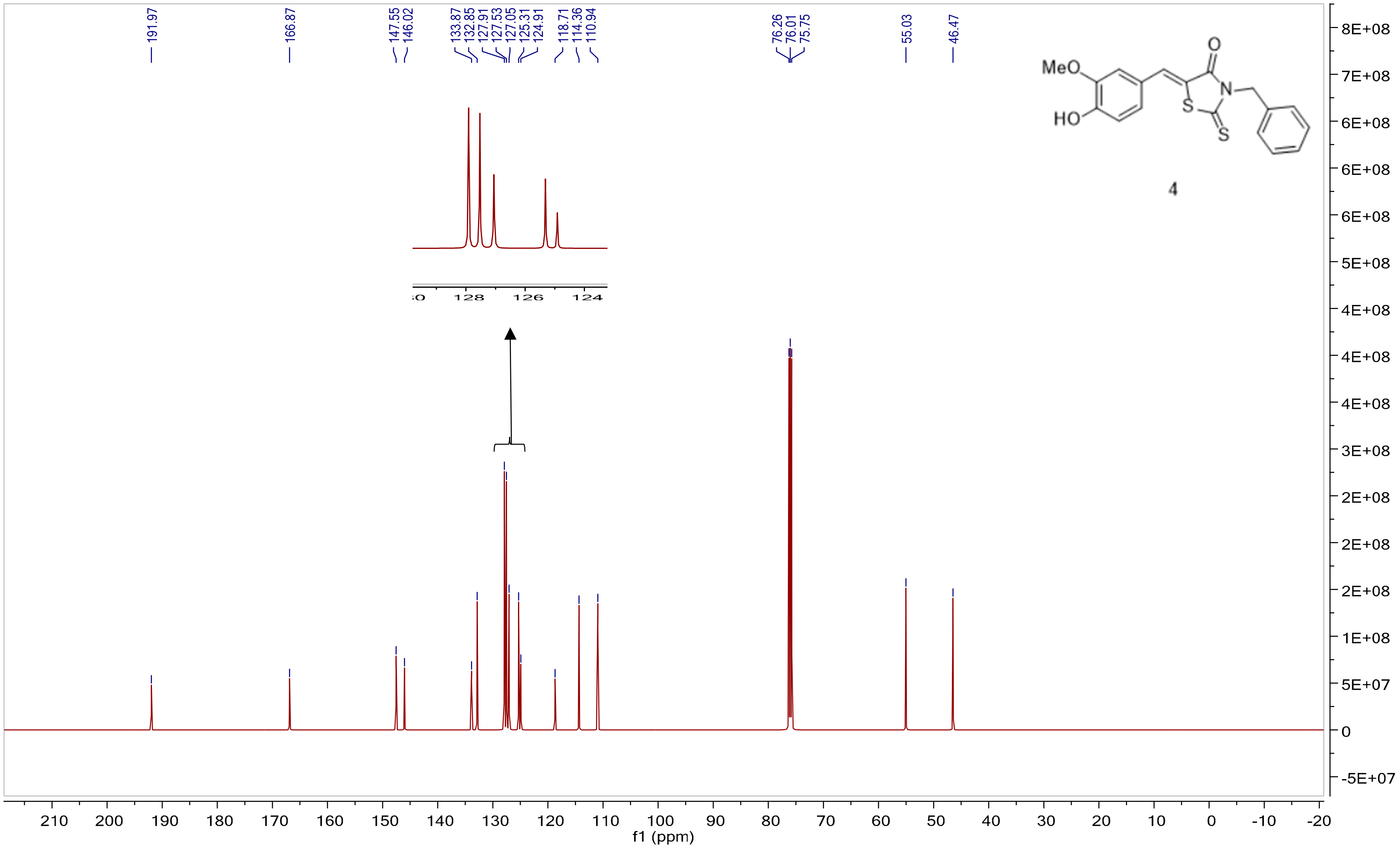

S23. ^{13}C NMR spectrum of analog **4**

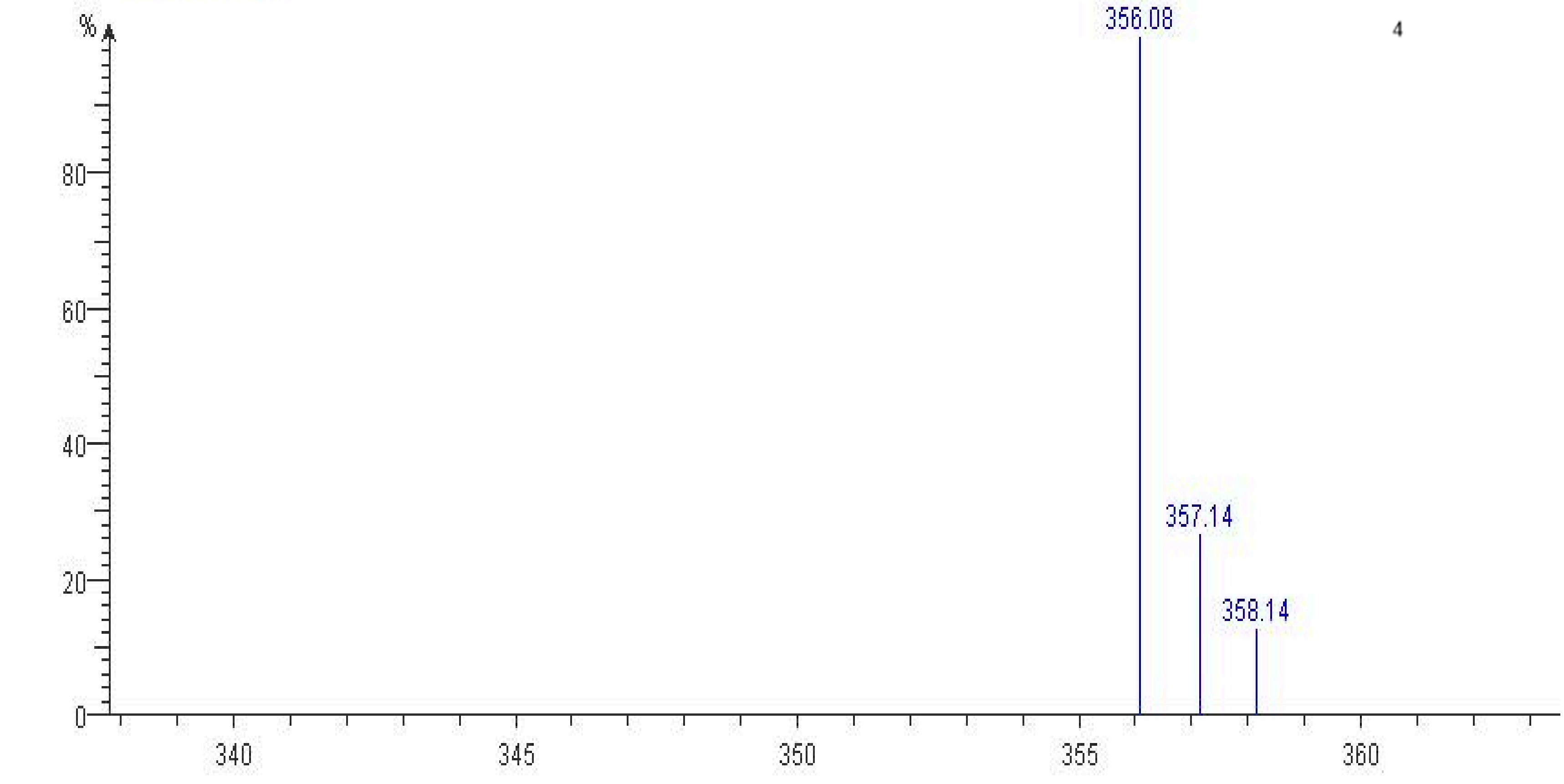

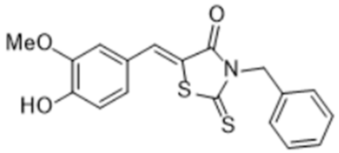

4

S24. LRMS (ESI–) spectrum of analog **4**

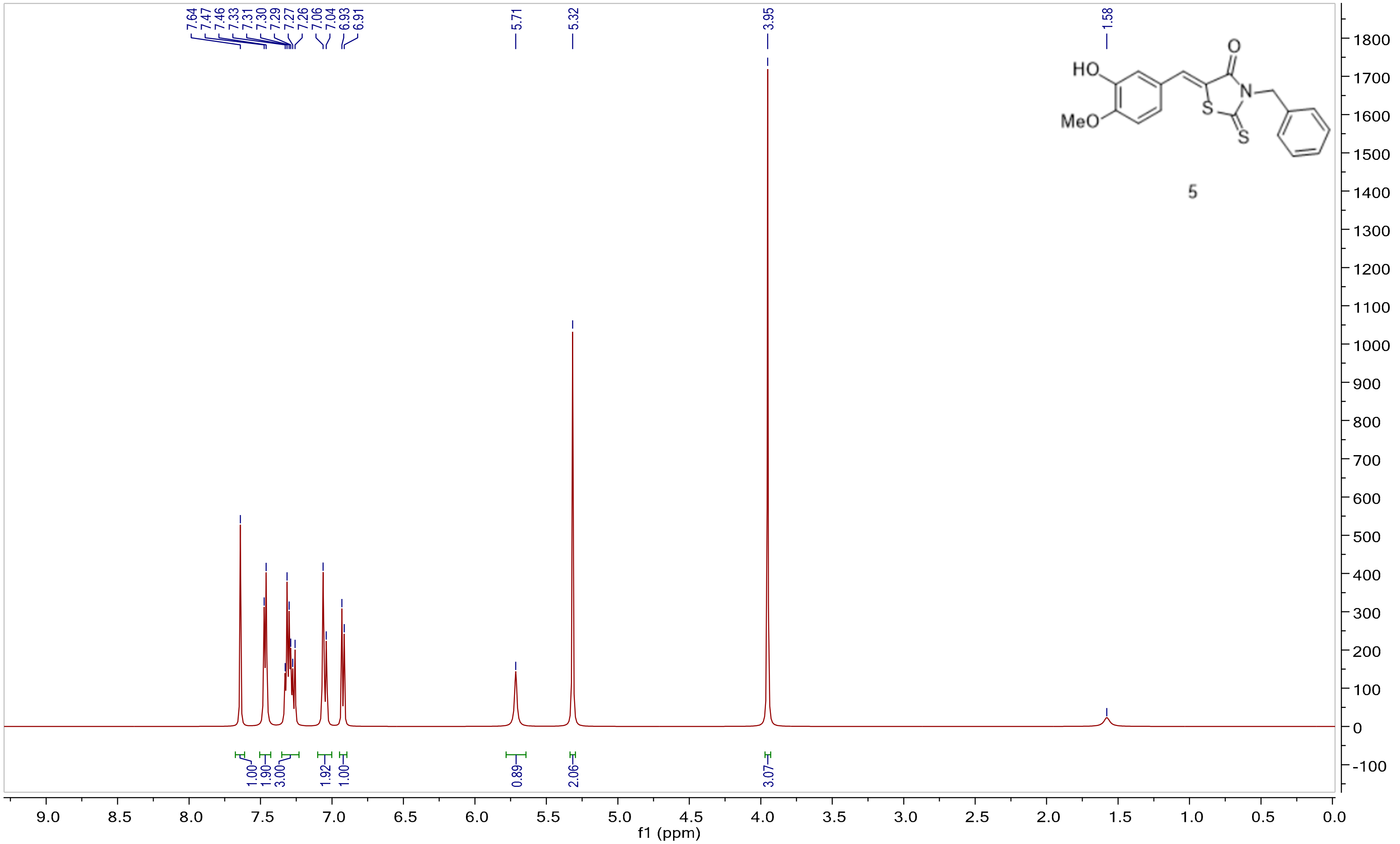

S25. ^{1}H NMR spectrum of analog **5**

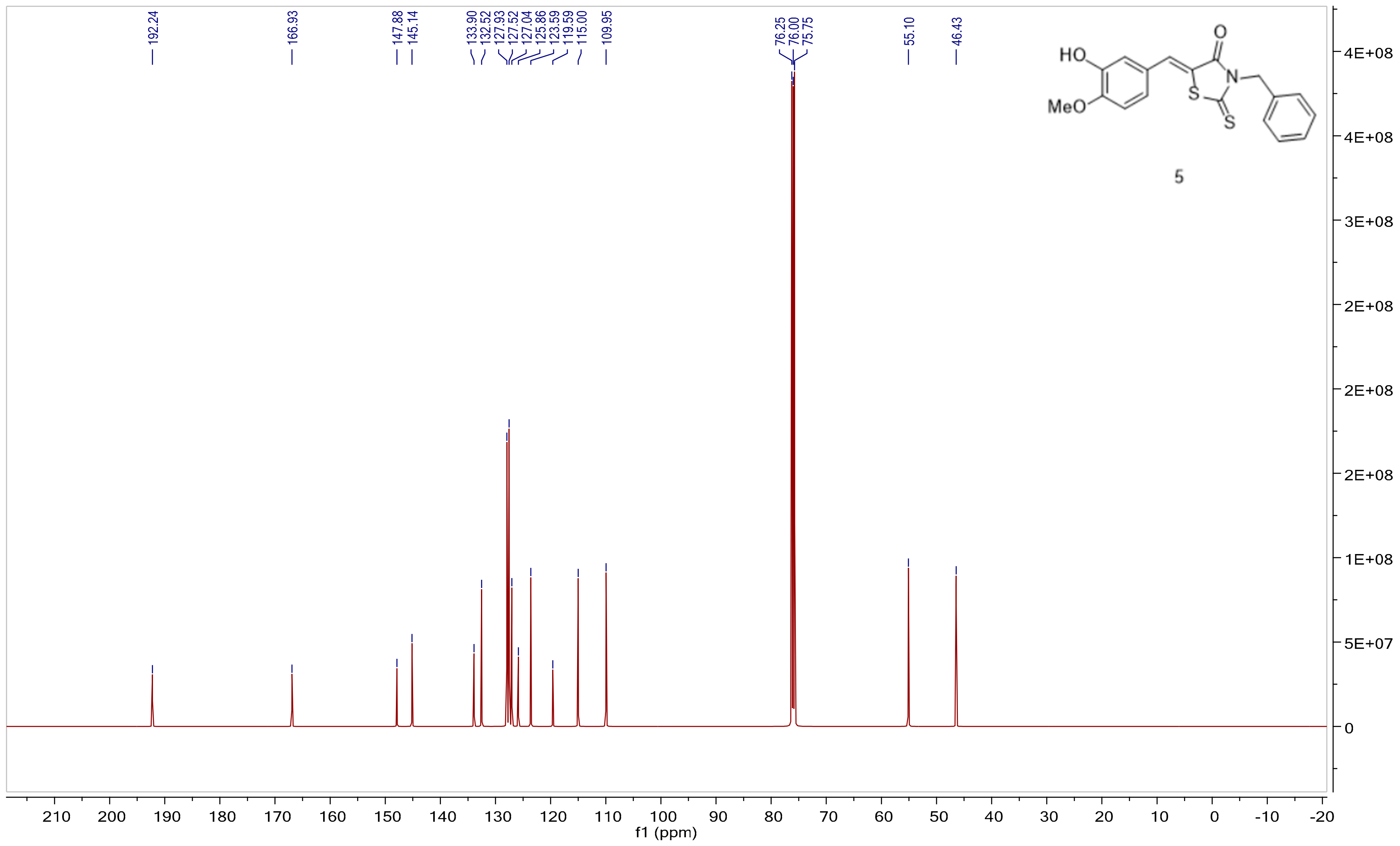

S26. ^{13}C NMR spectrum of analog **5**

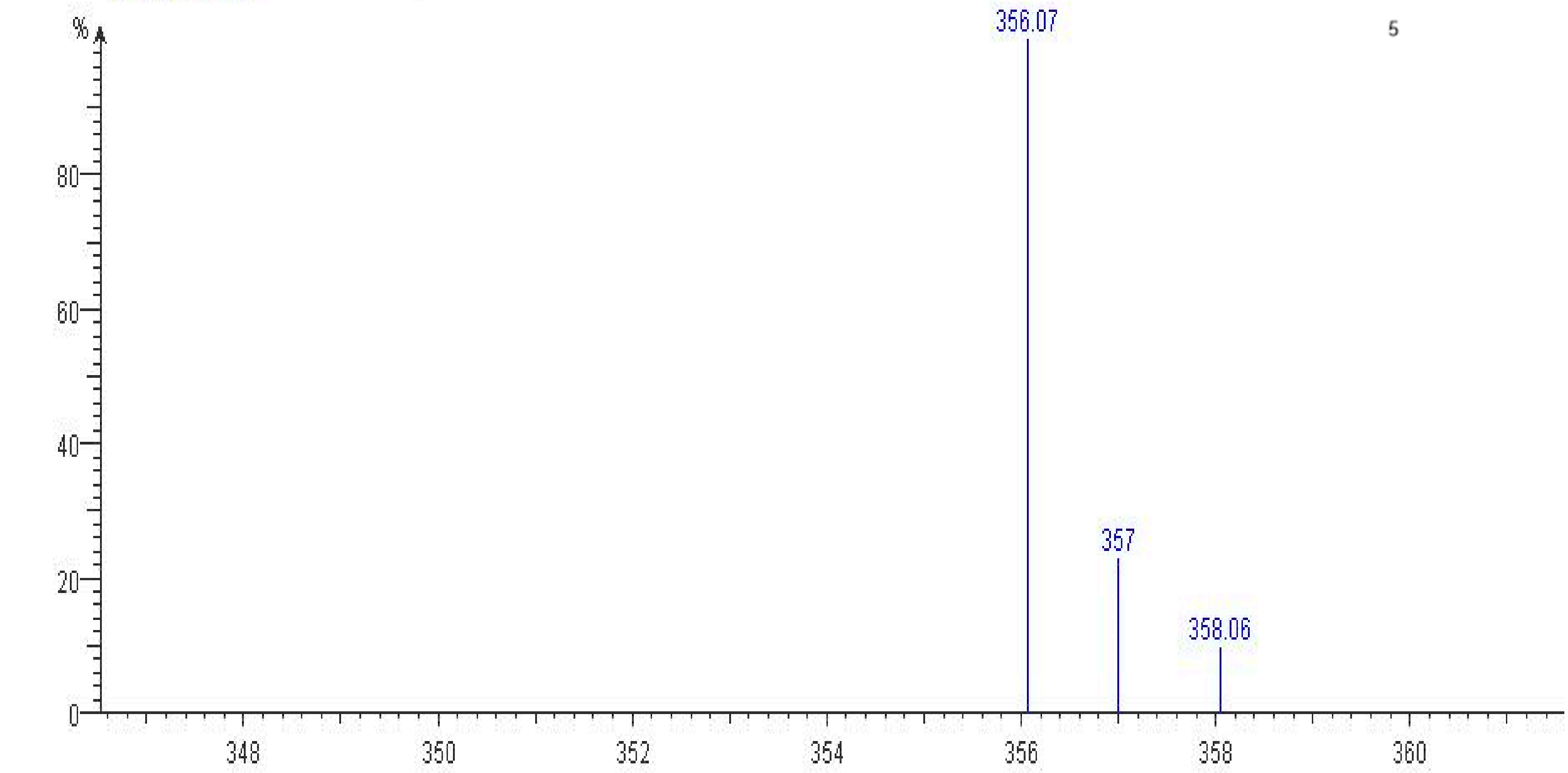

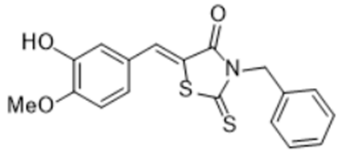

5

S27. LRMS (ESI–) spectrum of analog **5**

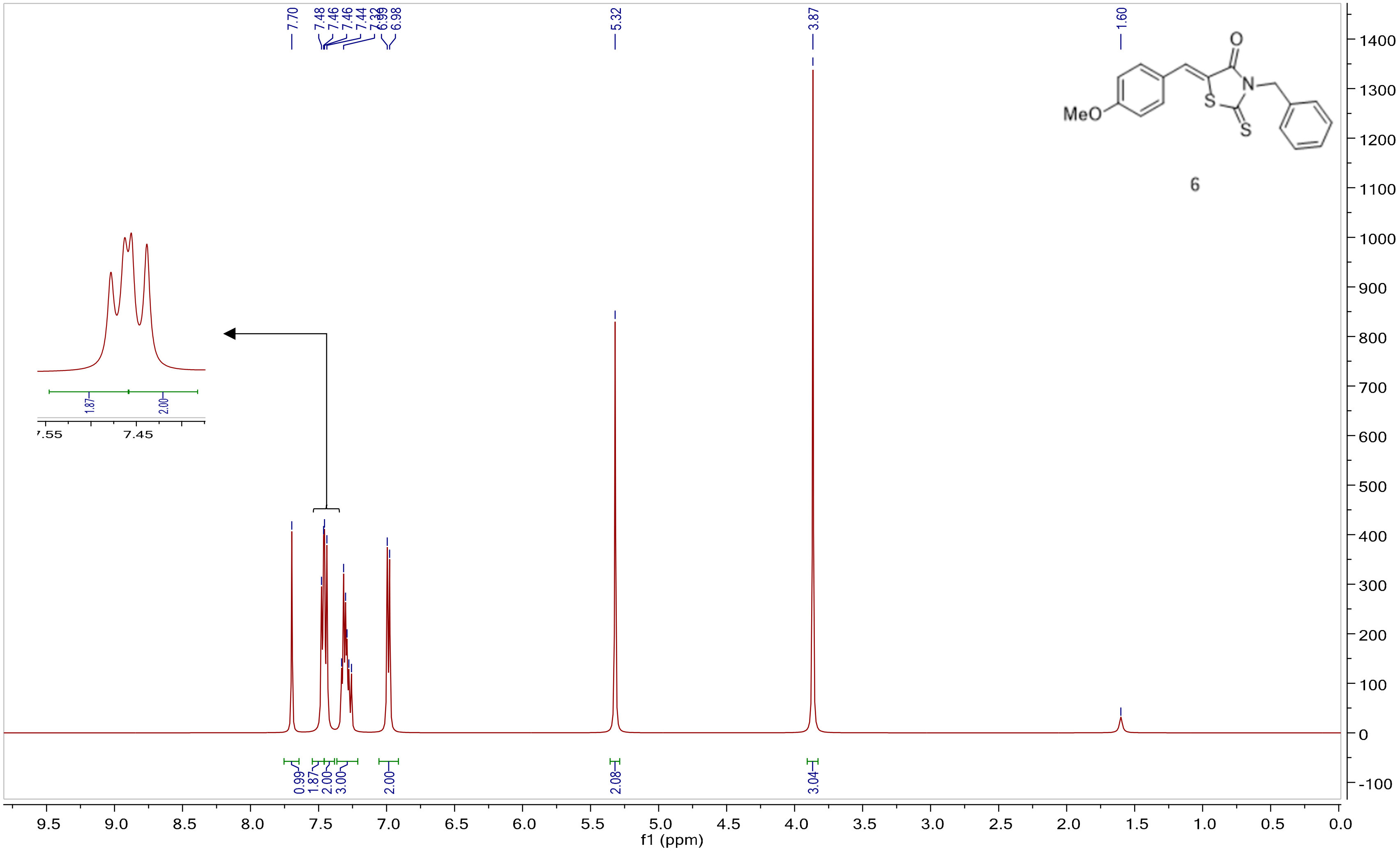

S28. ^{1}H NMR spectrum of analog **6**

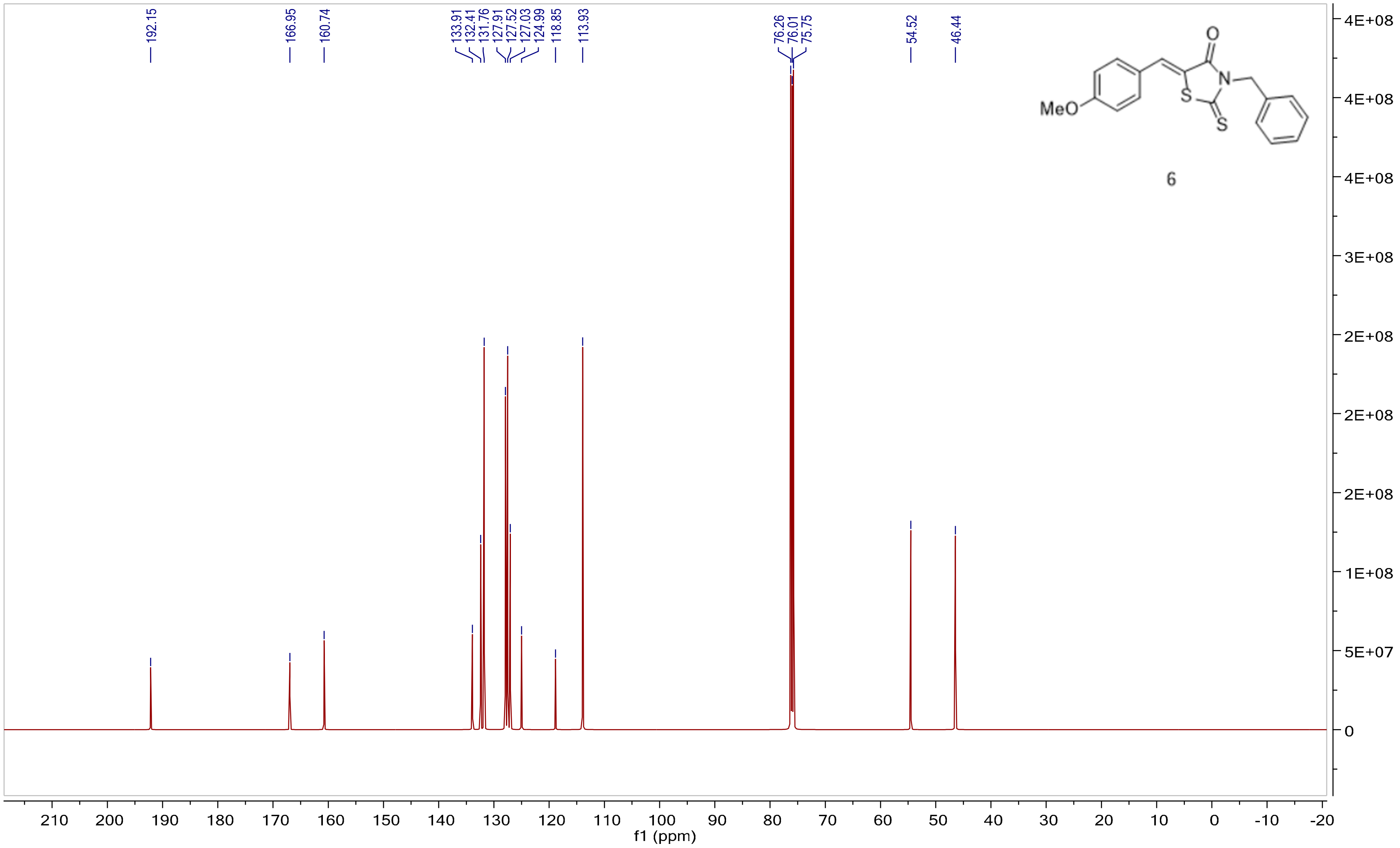

S29. ^{13}C NMR spectrum of analog **6**

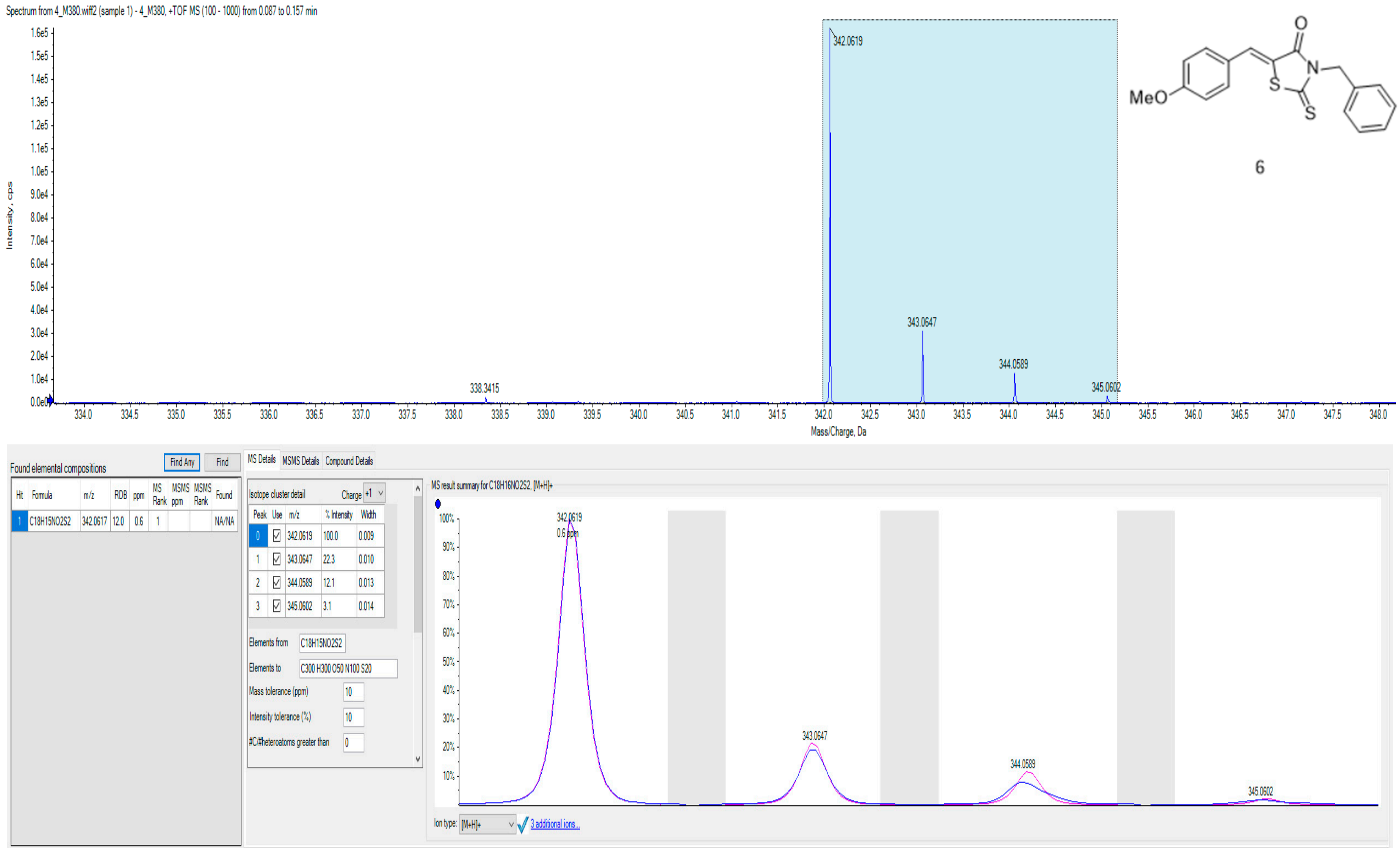

S30-1. HRMS (ESI+) spectrum of analog **4**

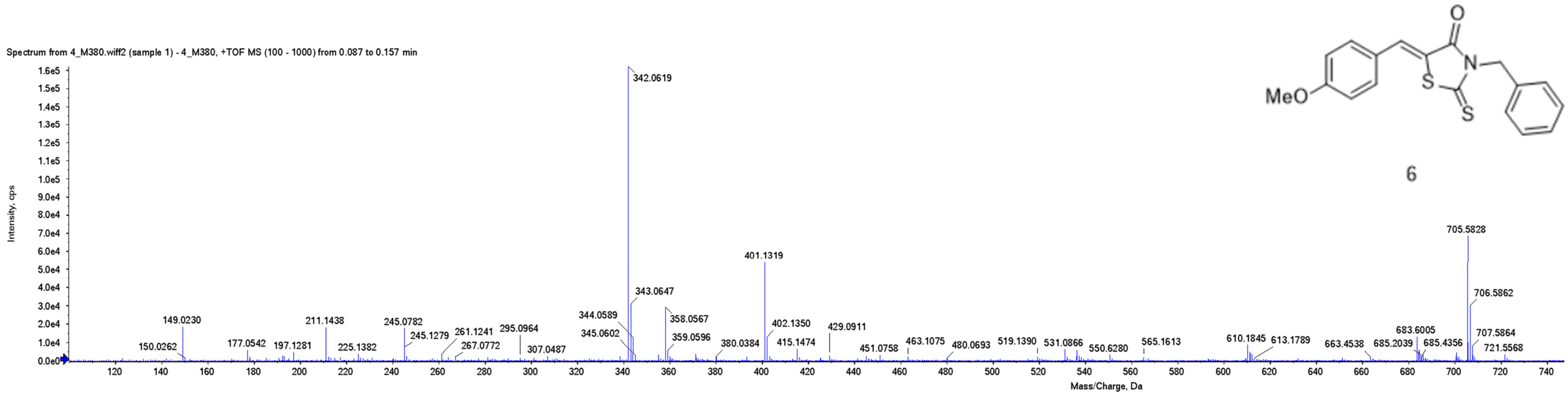

6

S30-2. HRMS (ESI+) spectrum of analog **4**

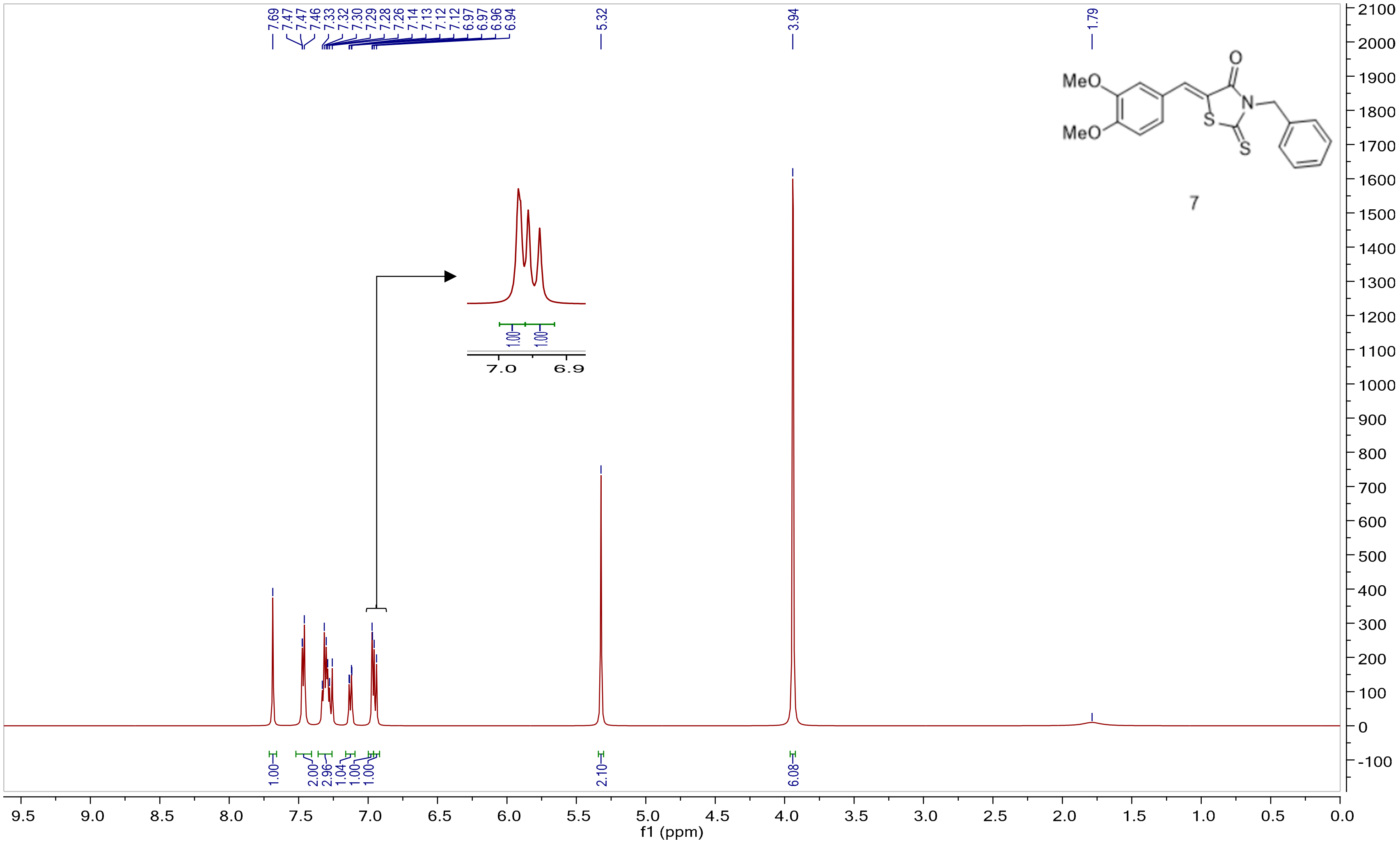

S31. ¹H NMR spectrum of analog **7**

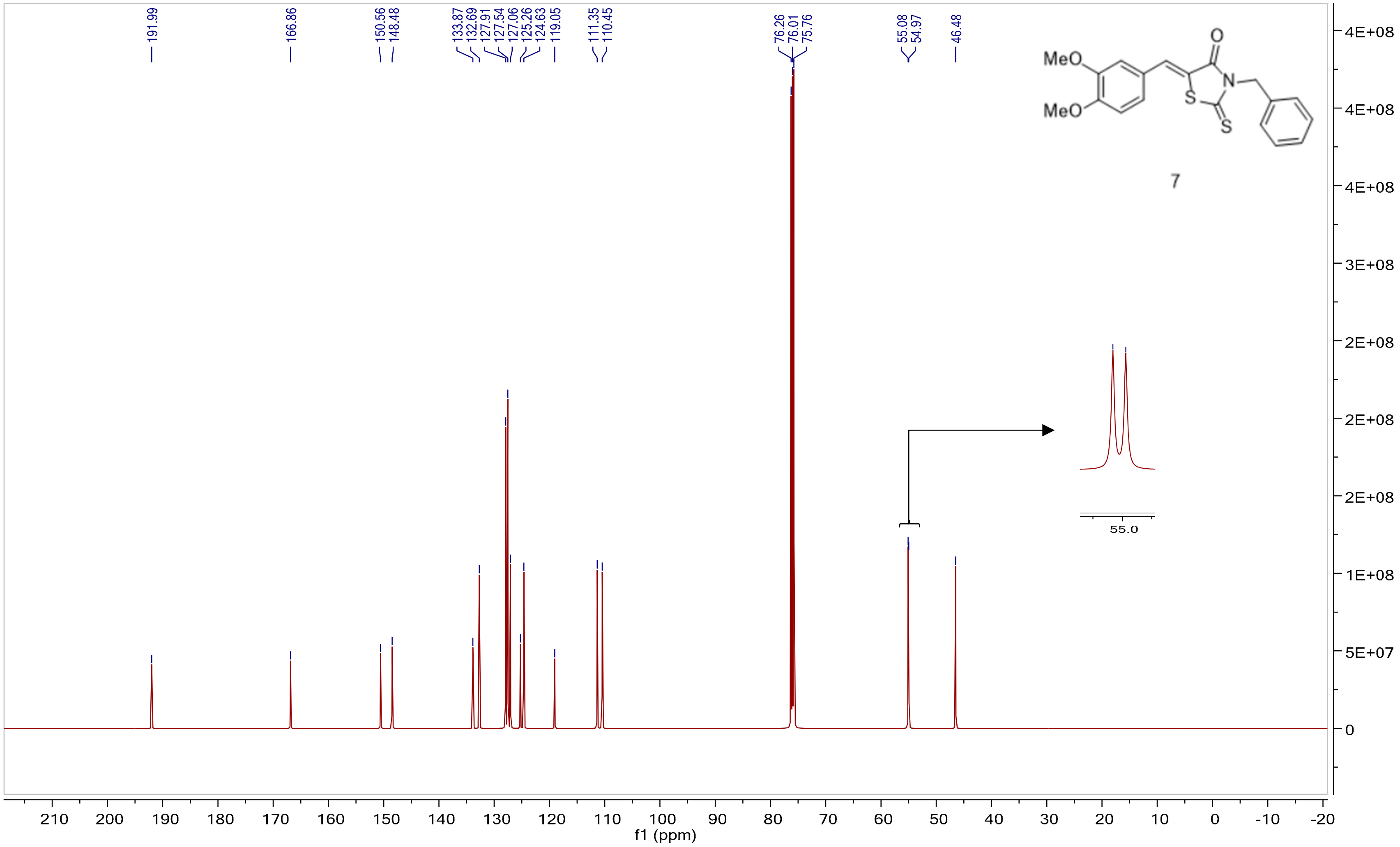

S32. ^{13}C NMR spectrum of analog **7**

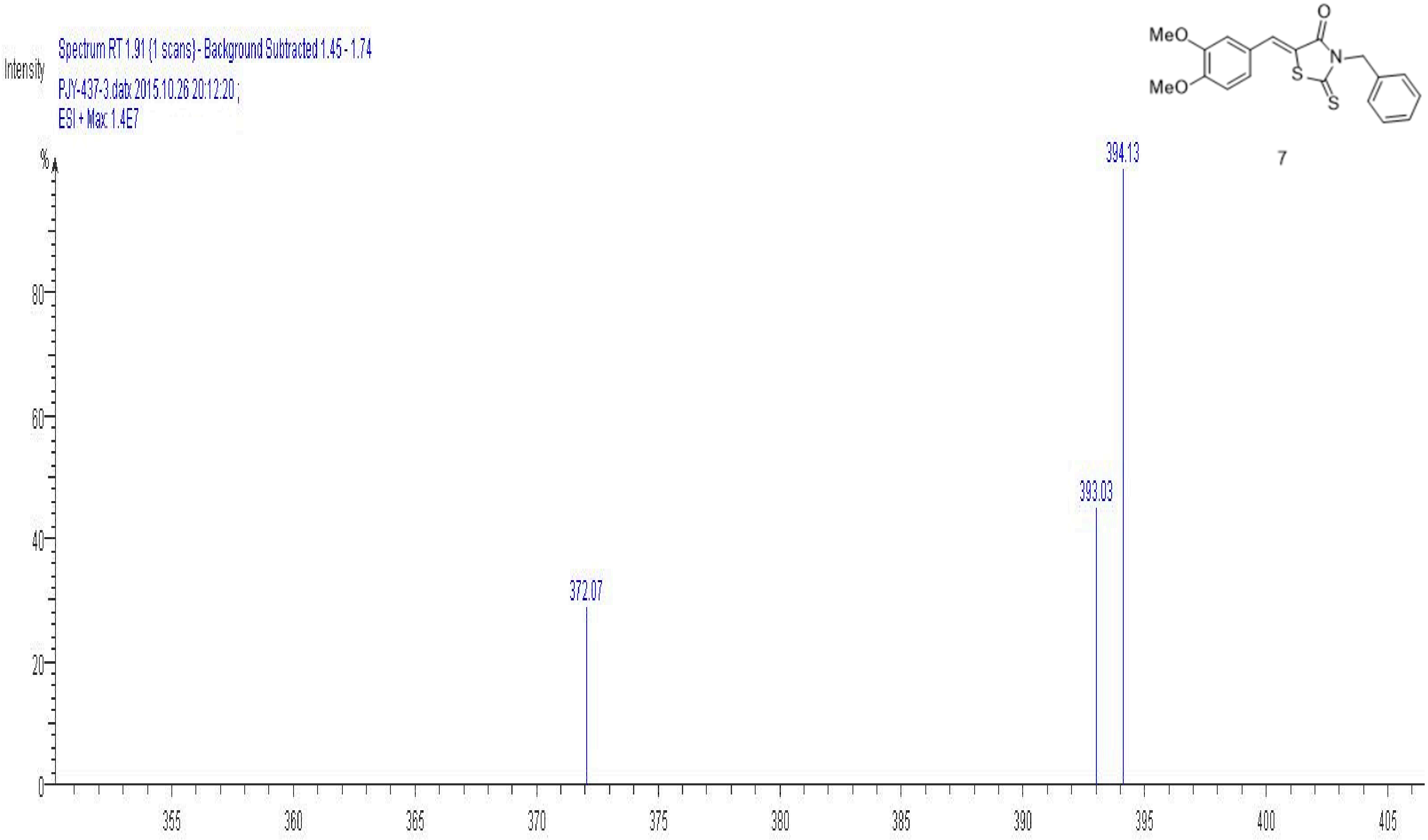

S33. LRMS (ESI+) spectrum of analog **7**

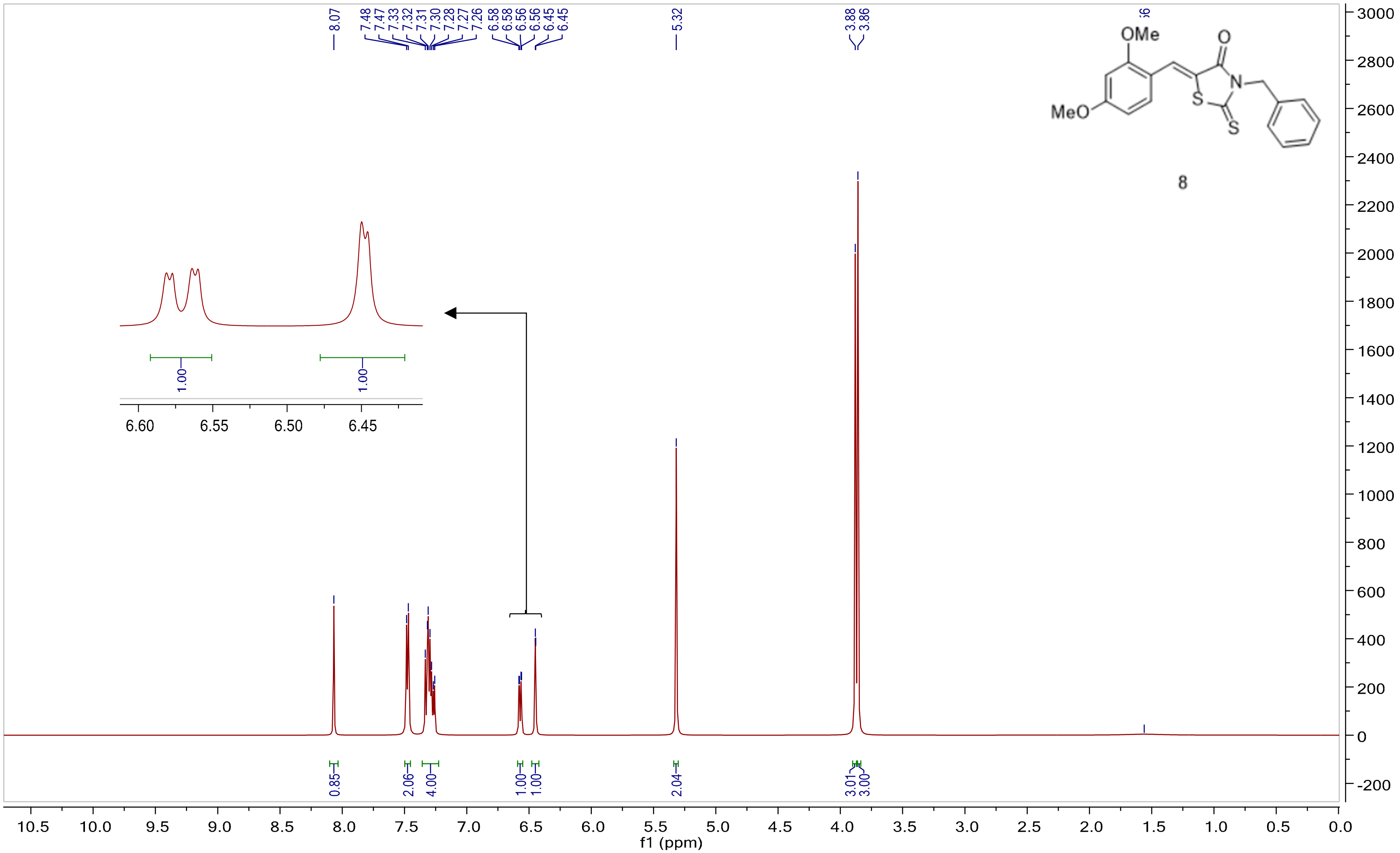

S34. ^{1}H NMR spectrum of analog **8**

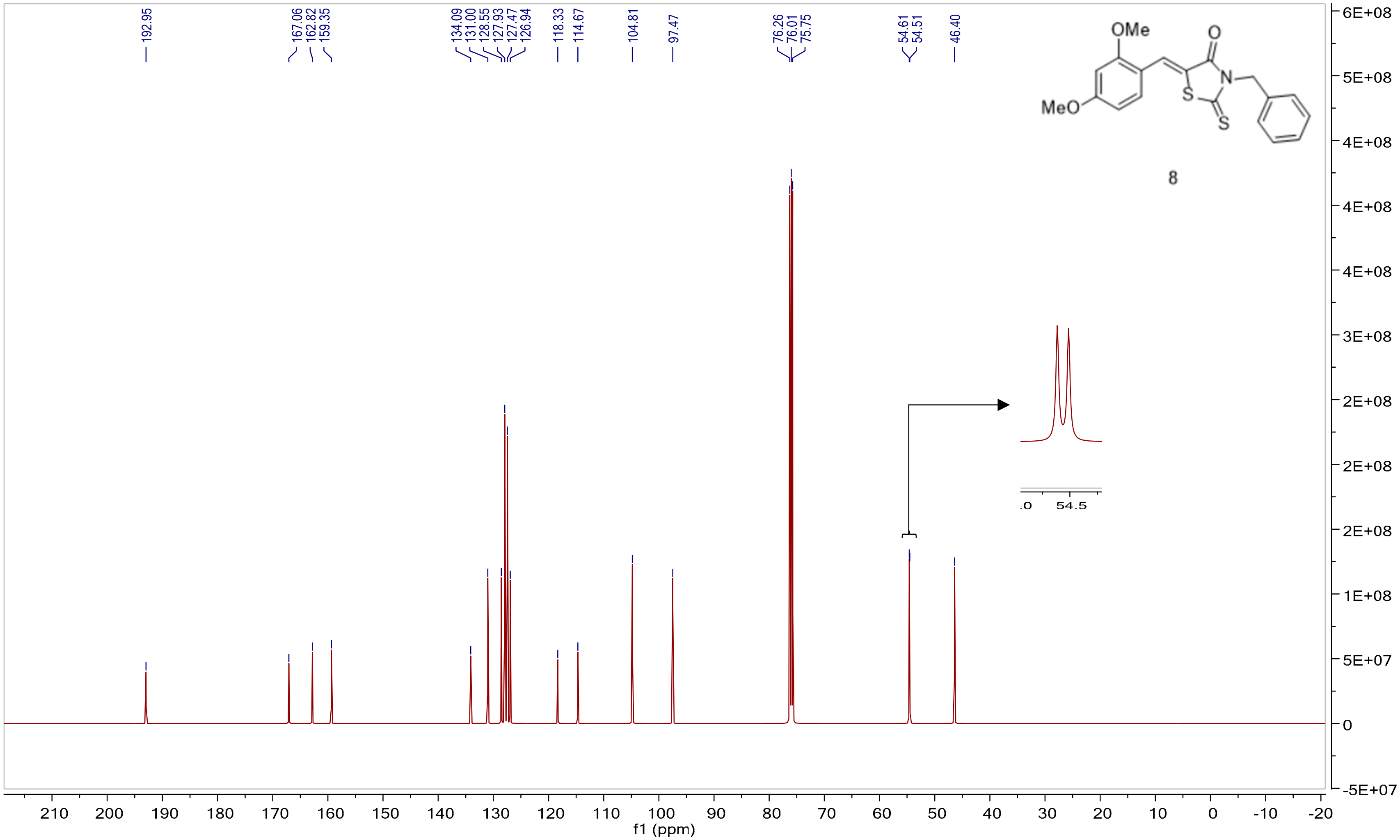

S35. ^{13}C NMR spectrum of analog **8**

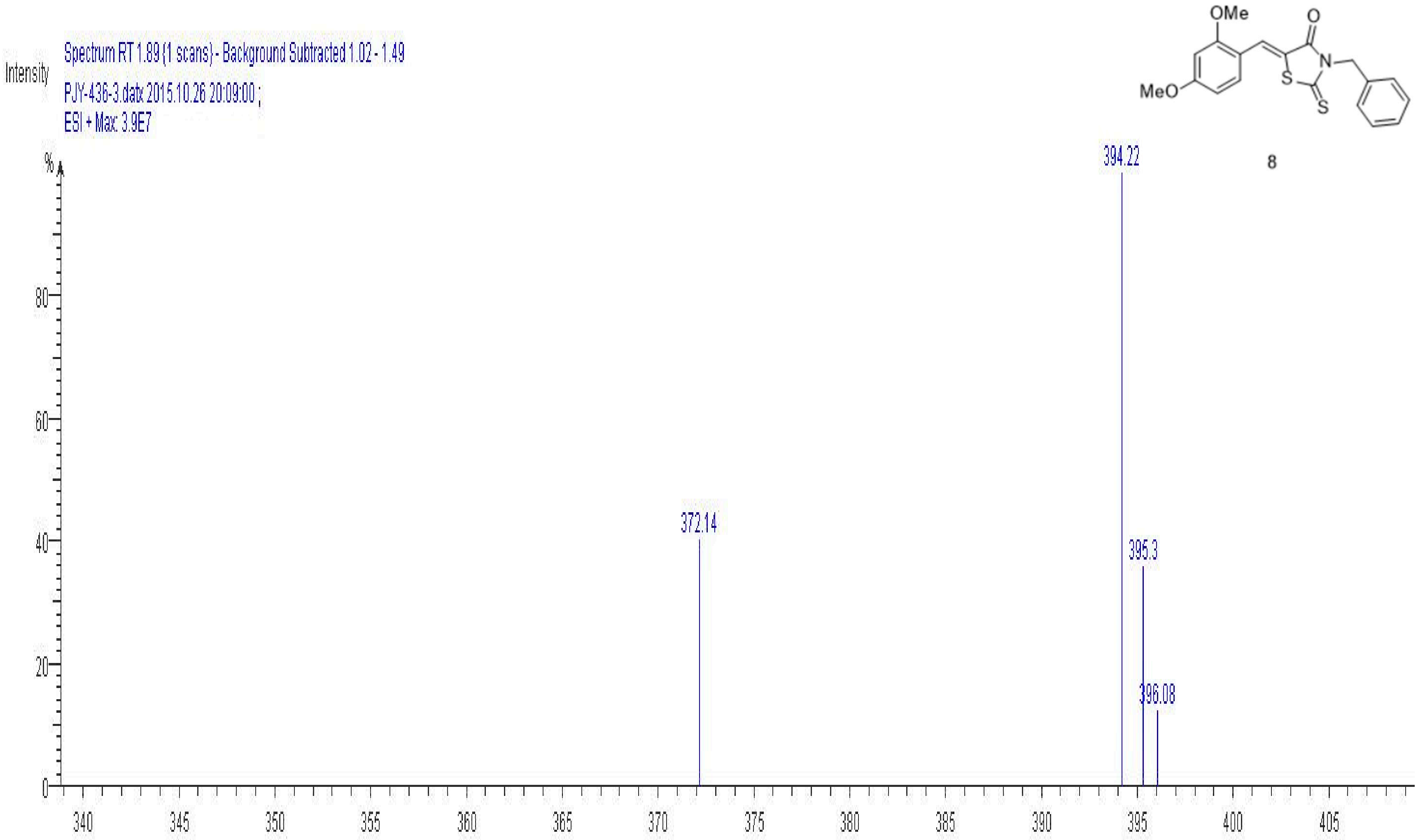

S36. LRMS (ESI+) spectrum of analog **8**

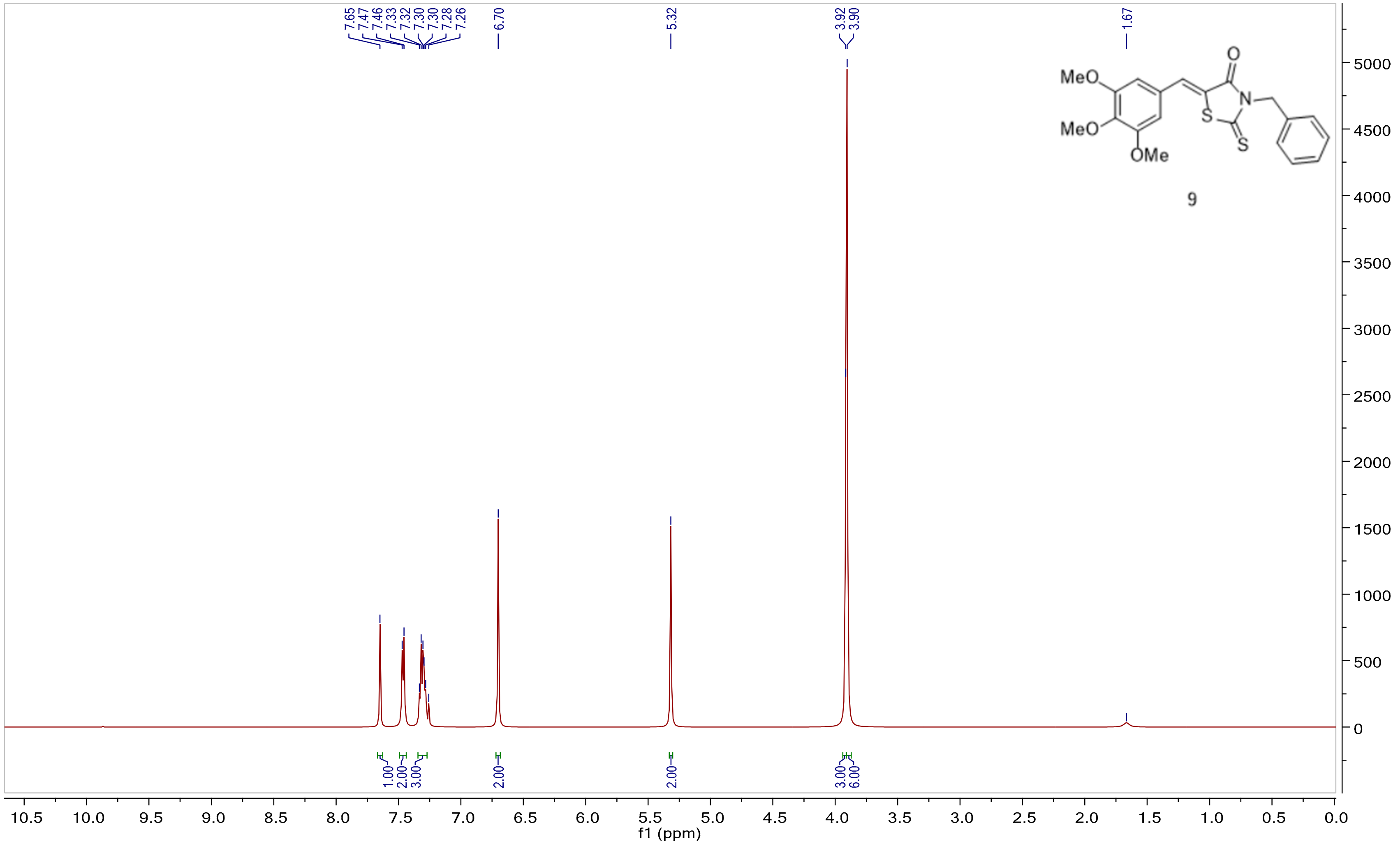

S37. ^{1}H NMR spectrum of analog **9**

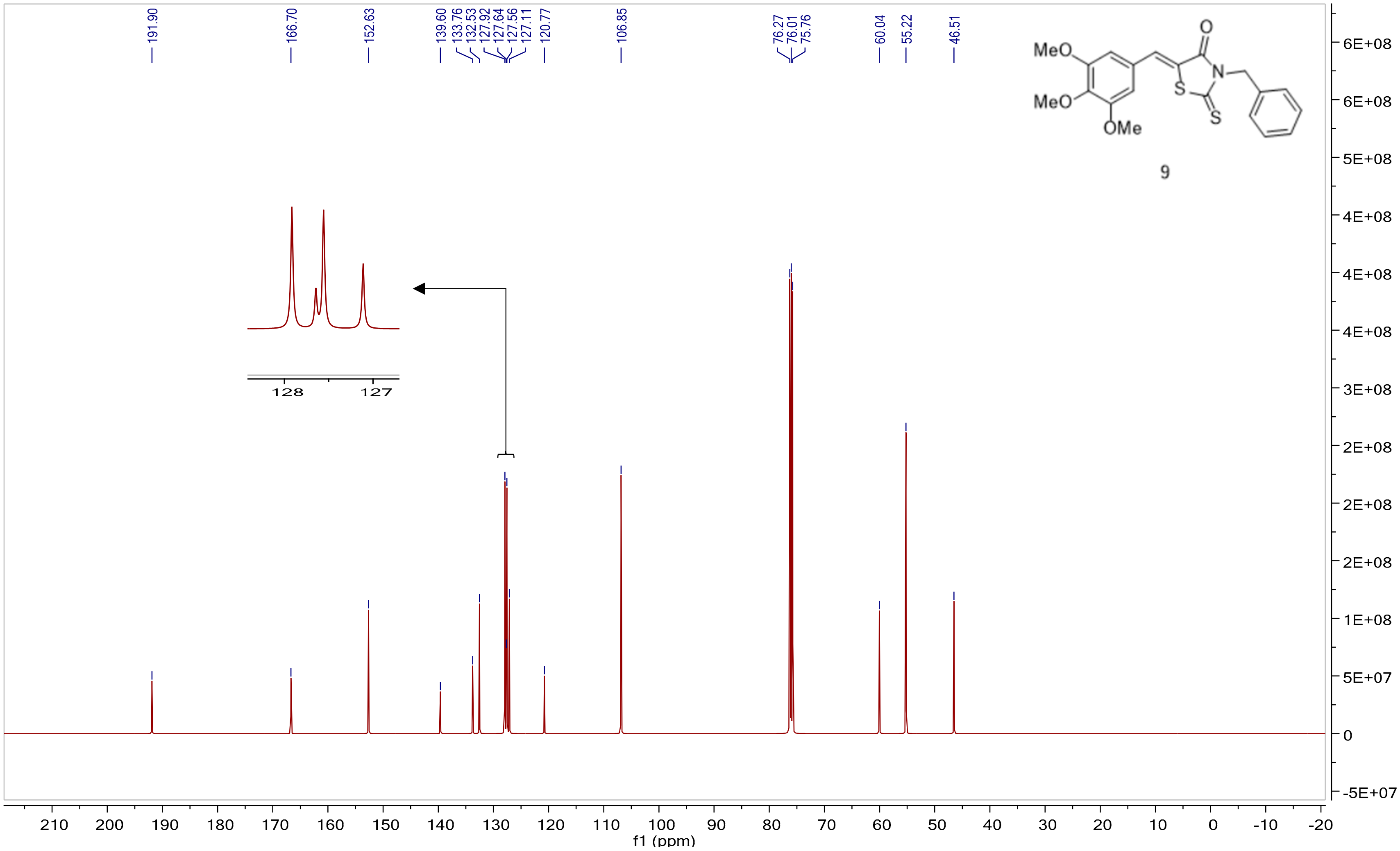

S38. ^{13}C NMR spectrum of analog **9**

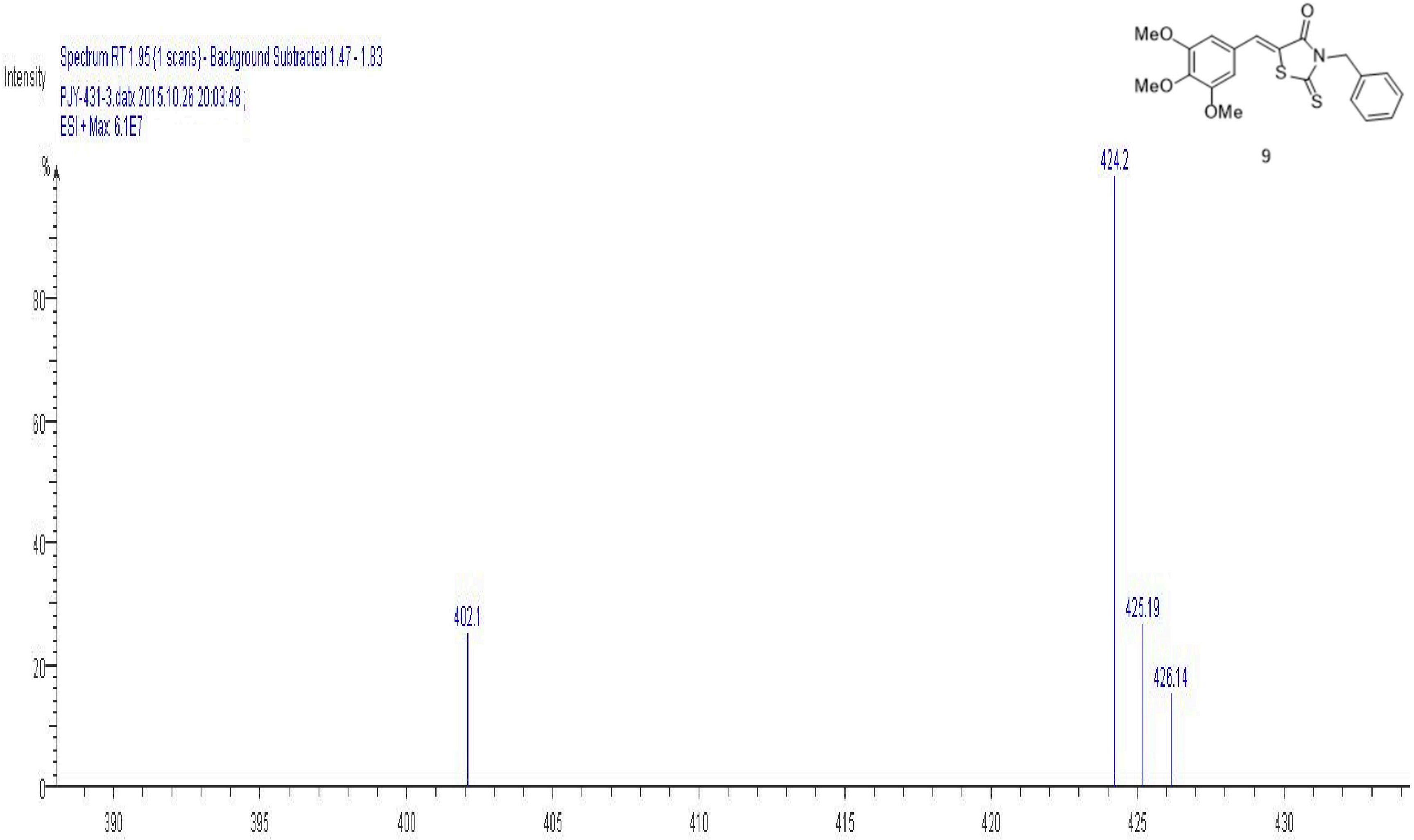

9

S39. LRMS (ESI+) spectrum of analog **9**

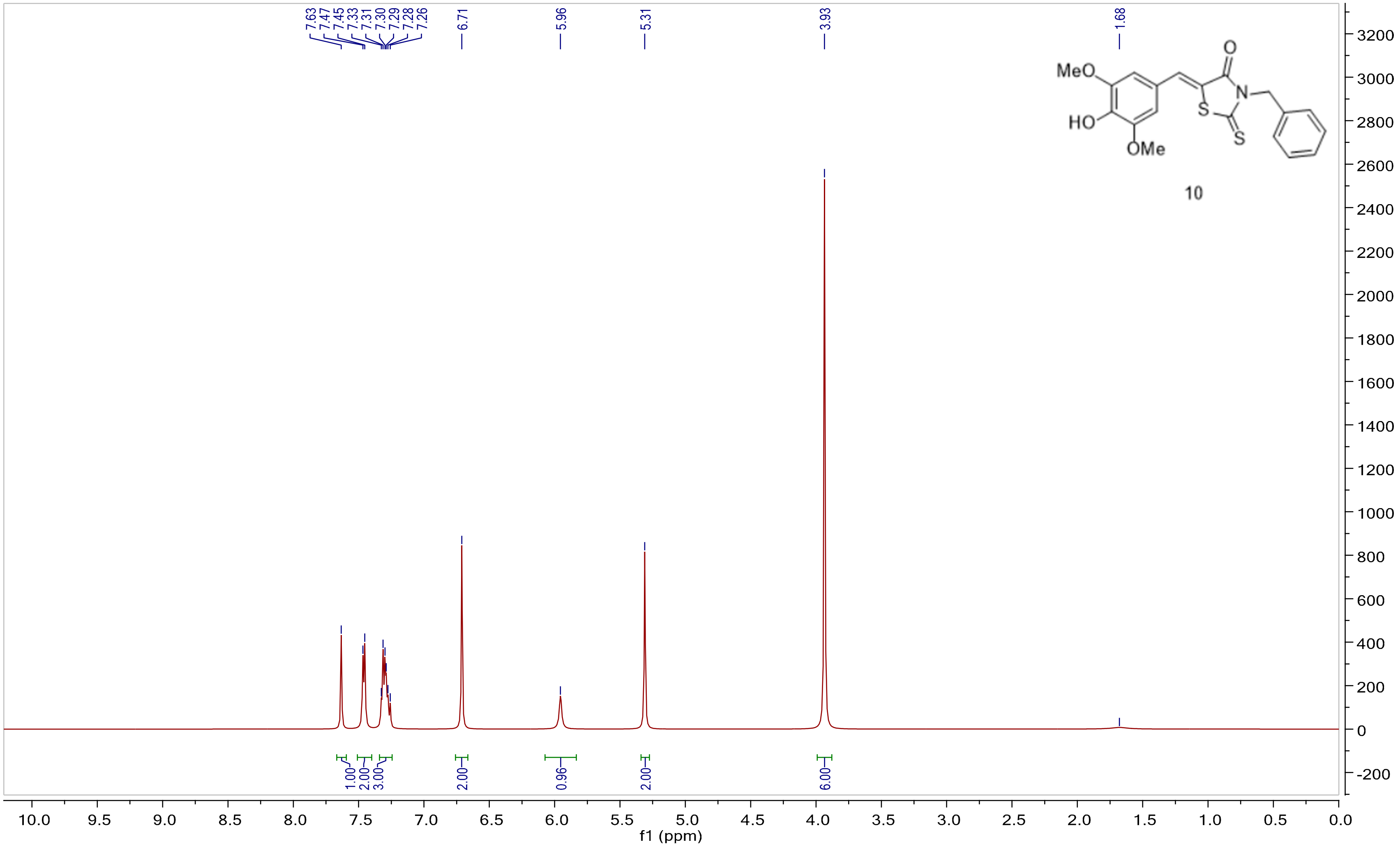

S40. ^{1}H NMR spectrum of analog **10**

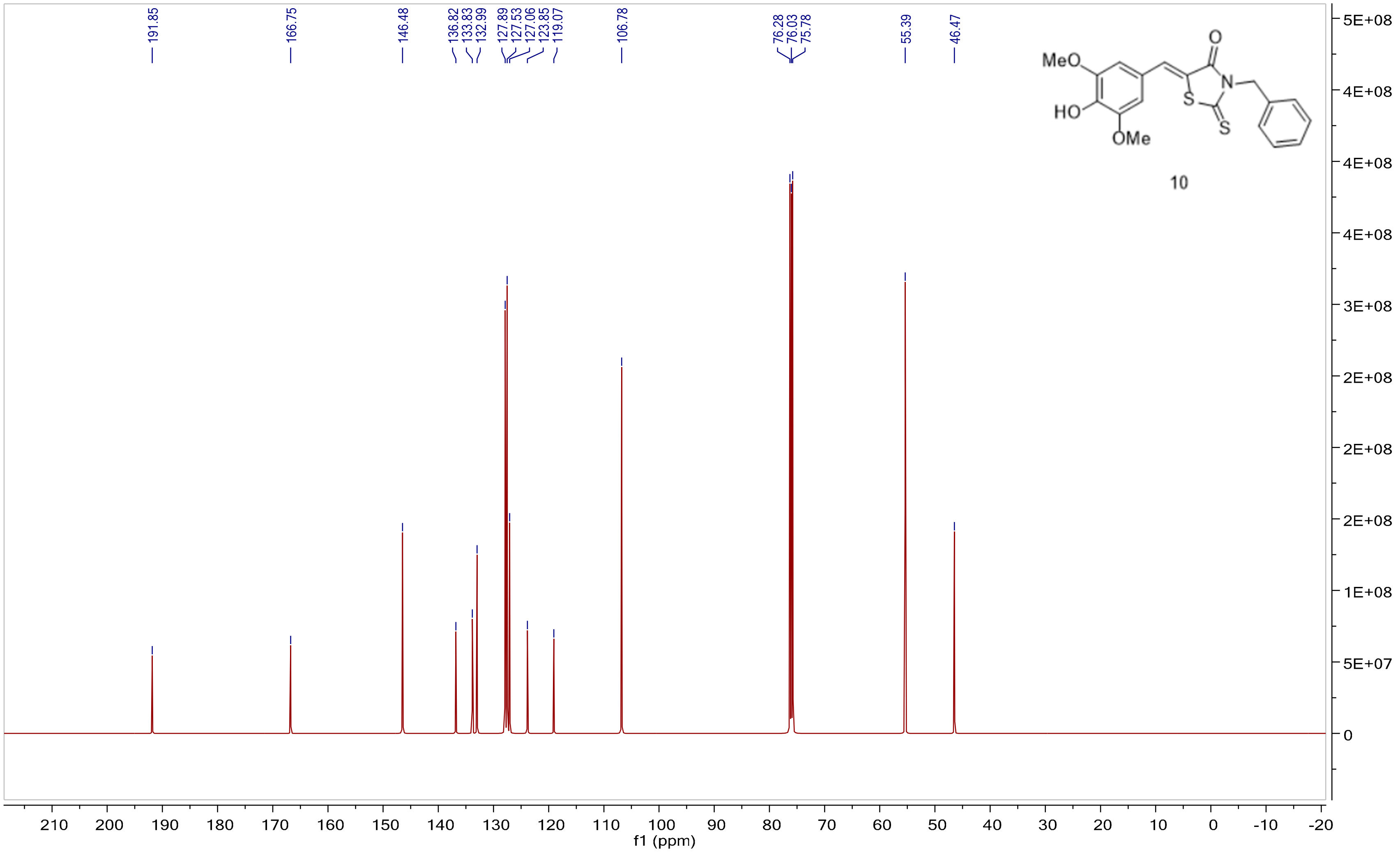

S41. ^{13}C NMR spectrum of analog **10**

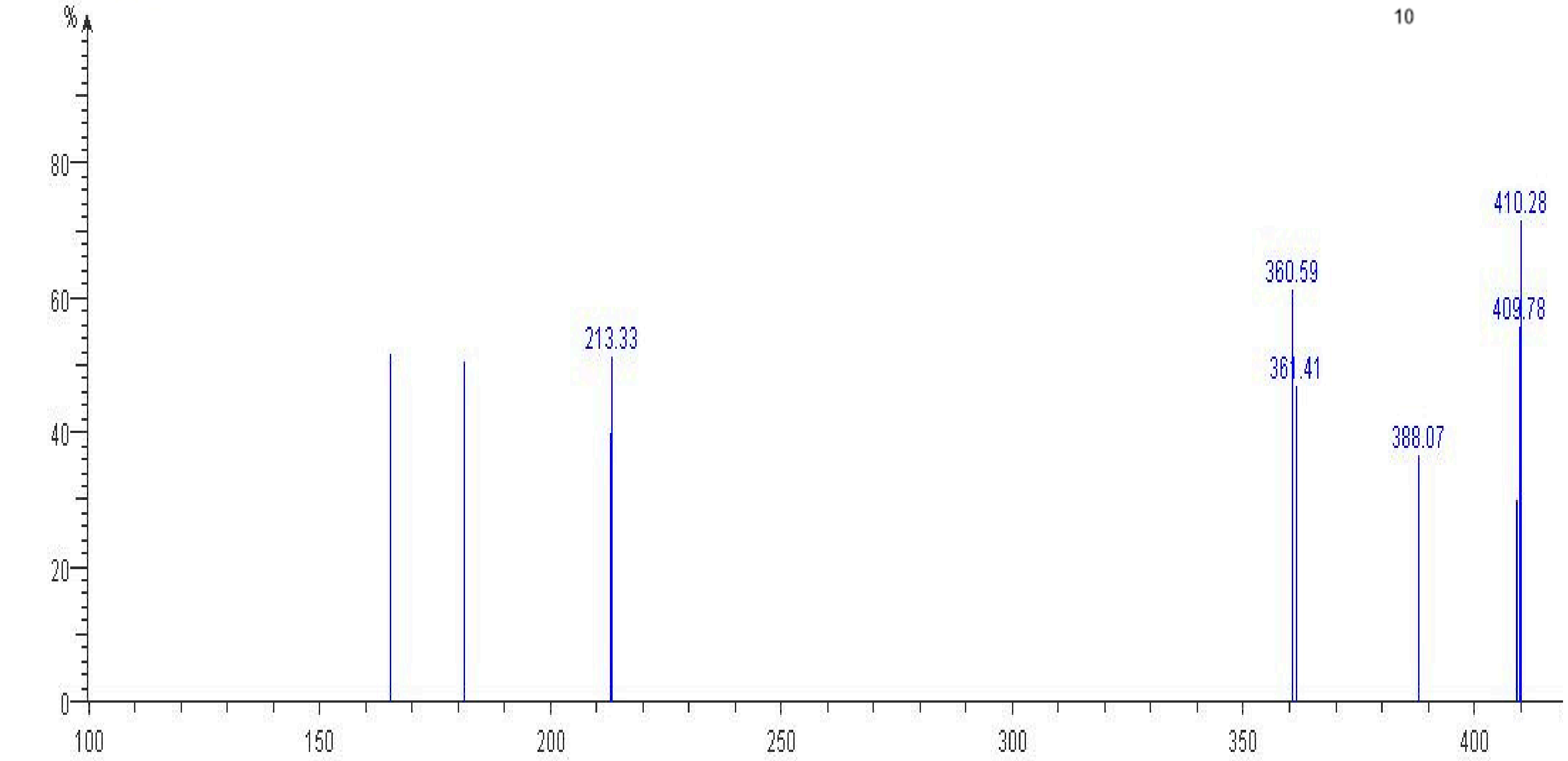

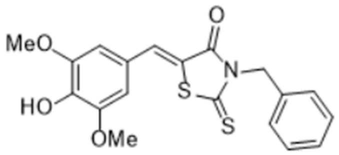

10

S42. LRMS (ESI+) spectrum of analog **10**

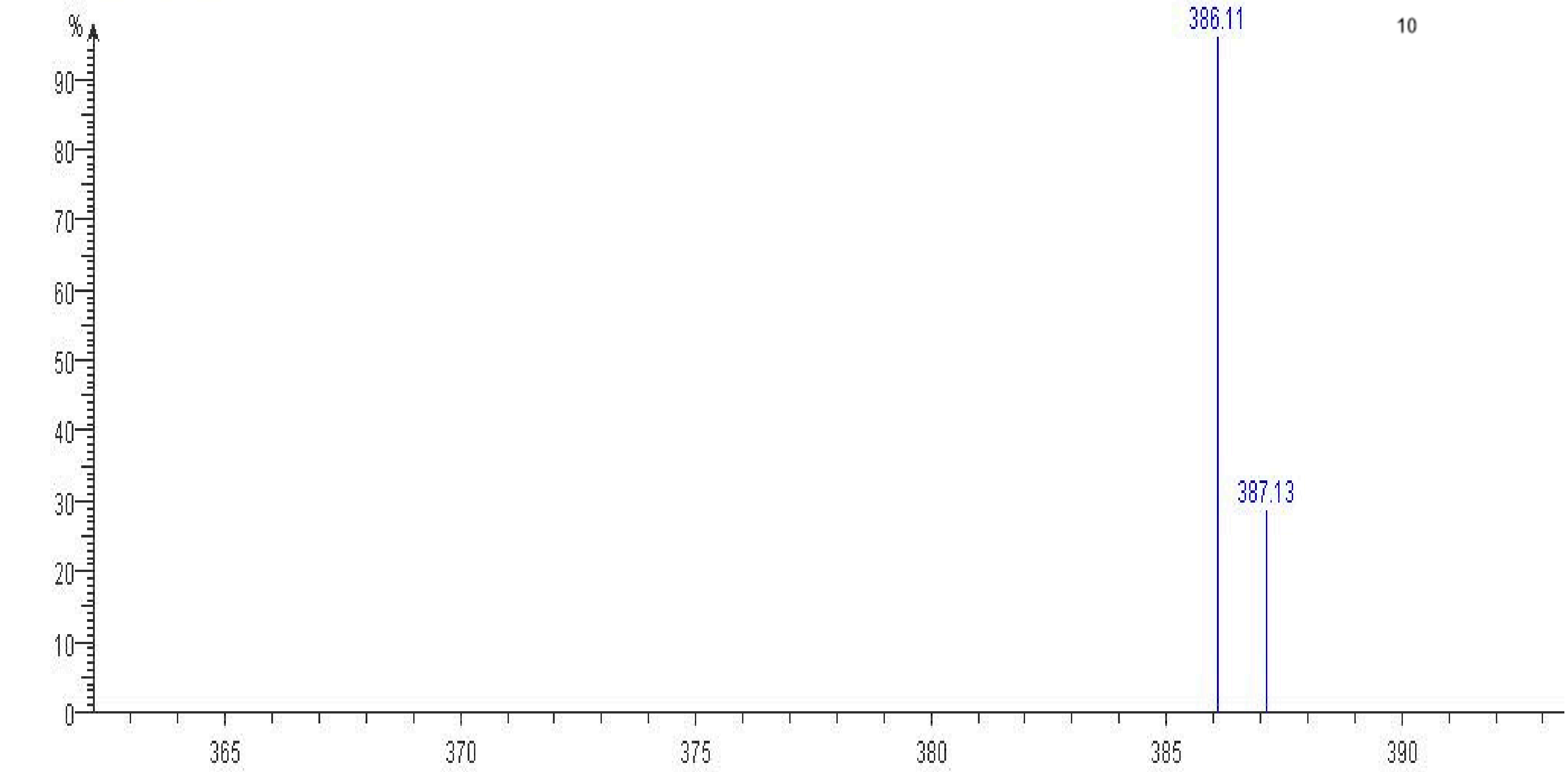

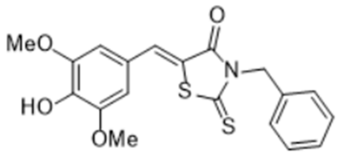

10

S43. LRMS (ESI–) spectrum of analog **10**

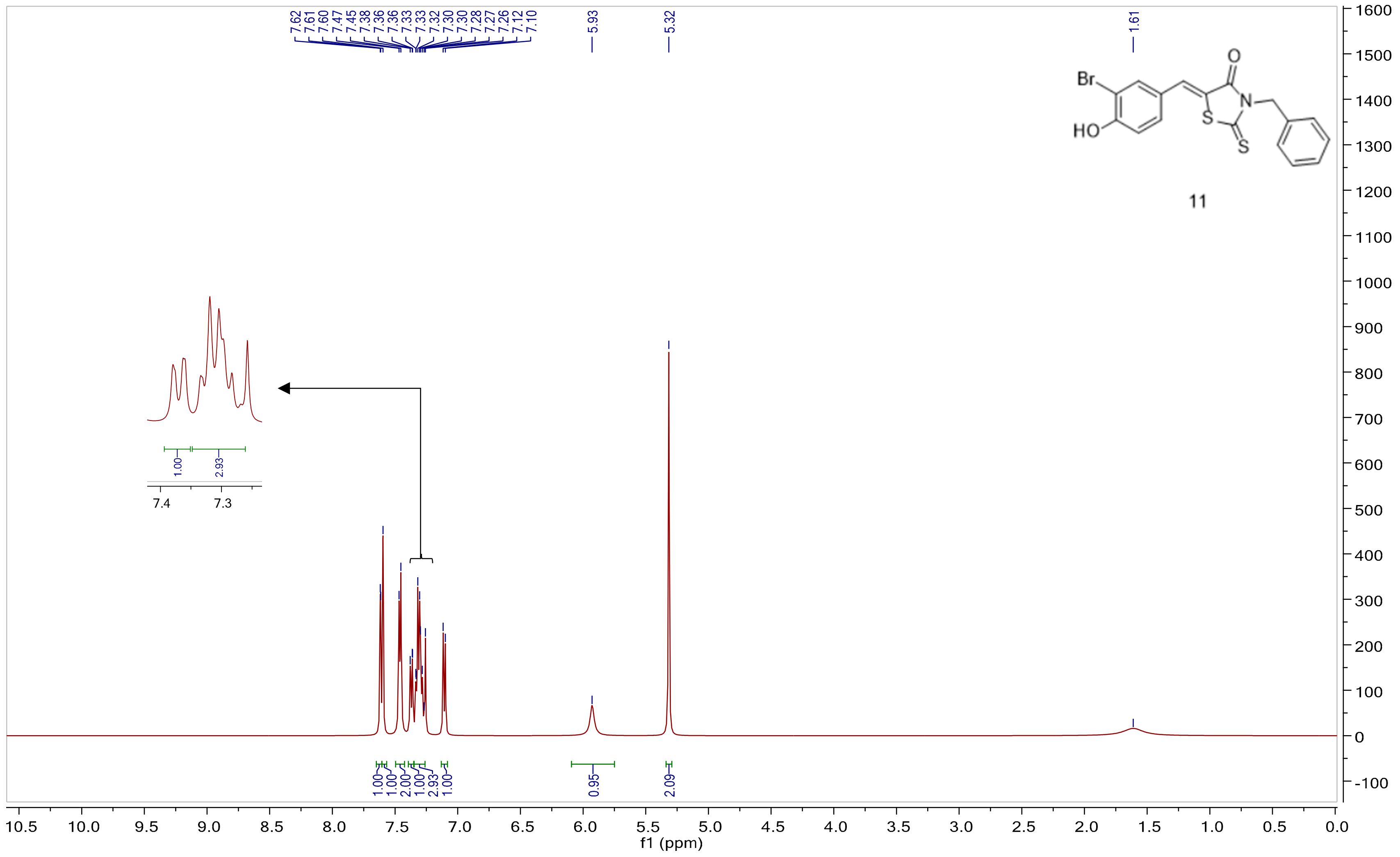

S44. ^{1}H NMR spectrum of analog **11**

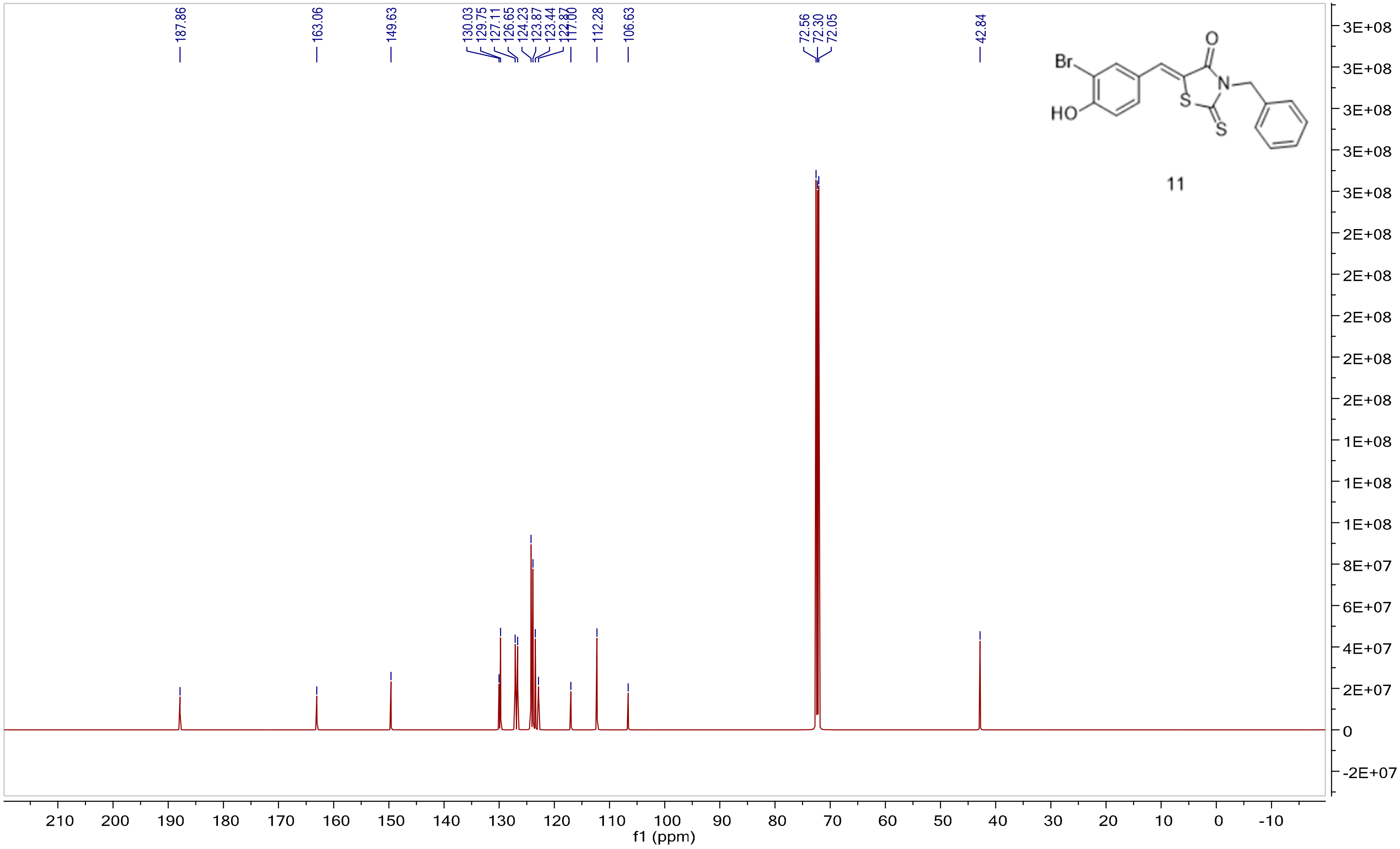

S45. ^{13}C NMR spectrum of analog **11**

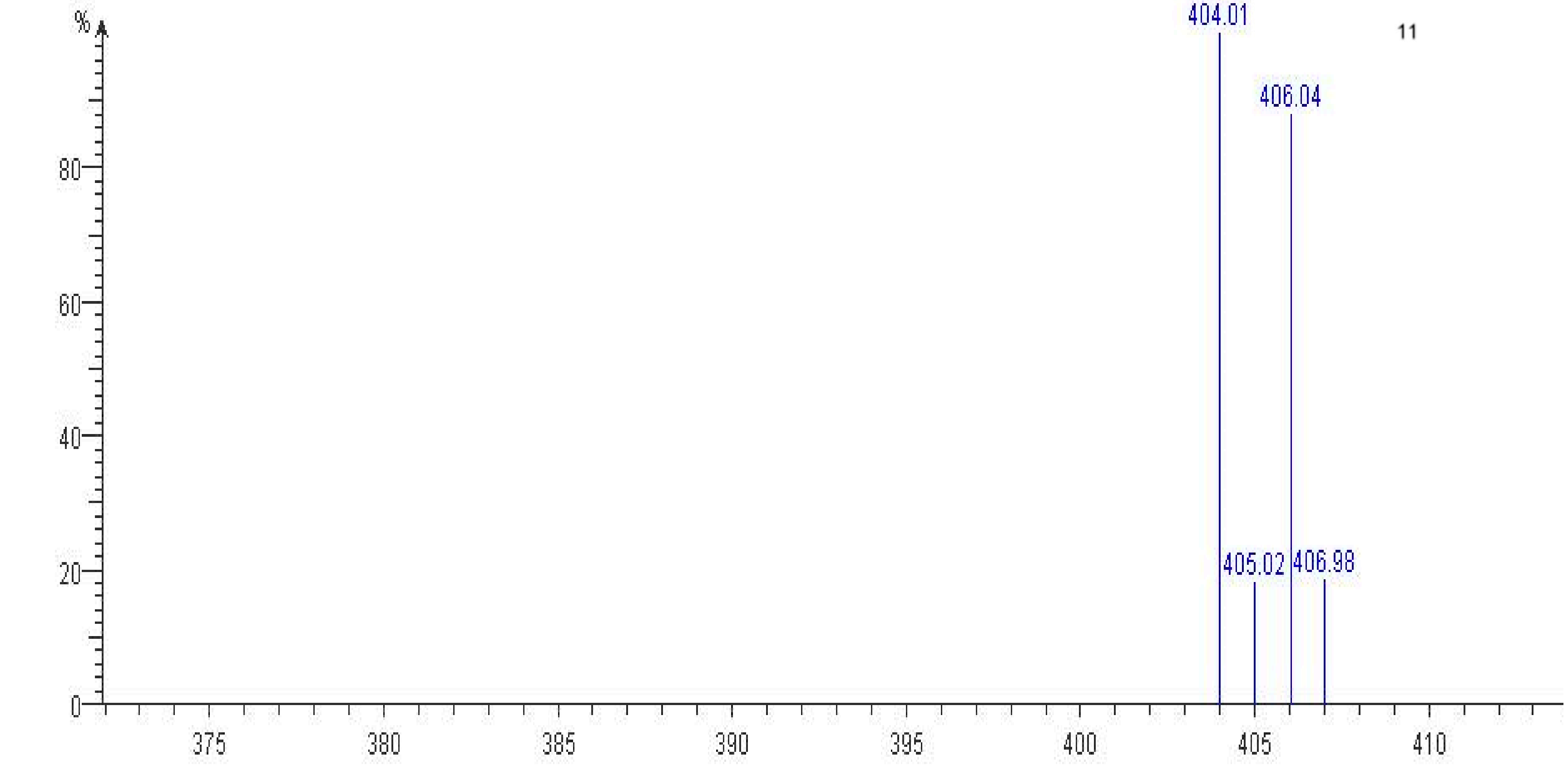

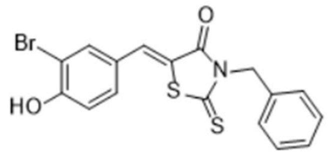

11

S46. LRMS (ESI–) spectrum of analog **11**

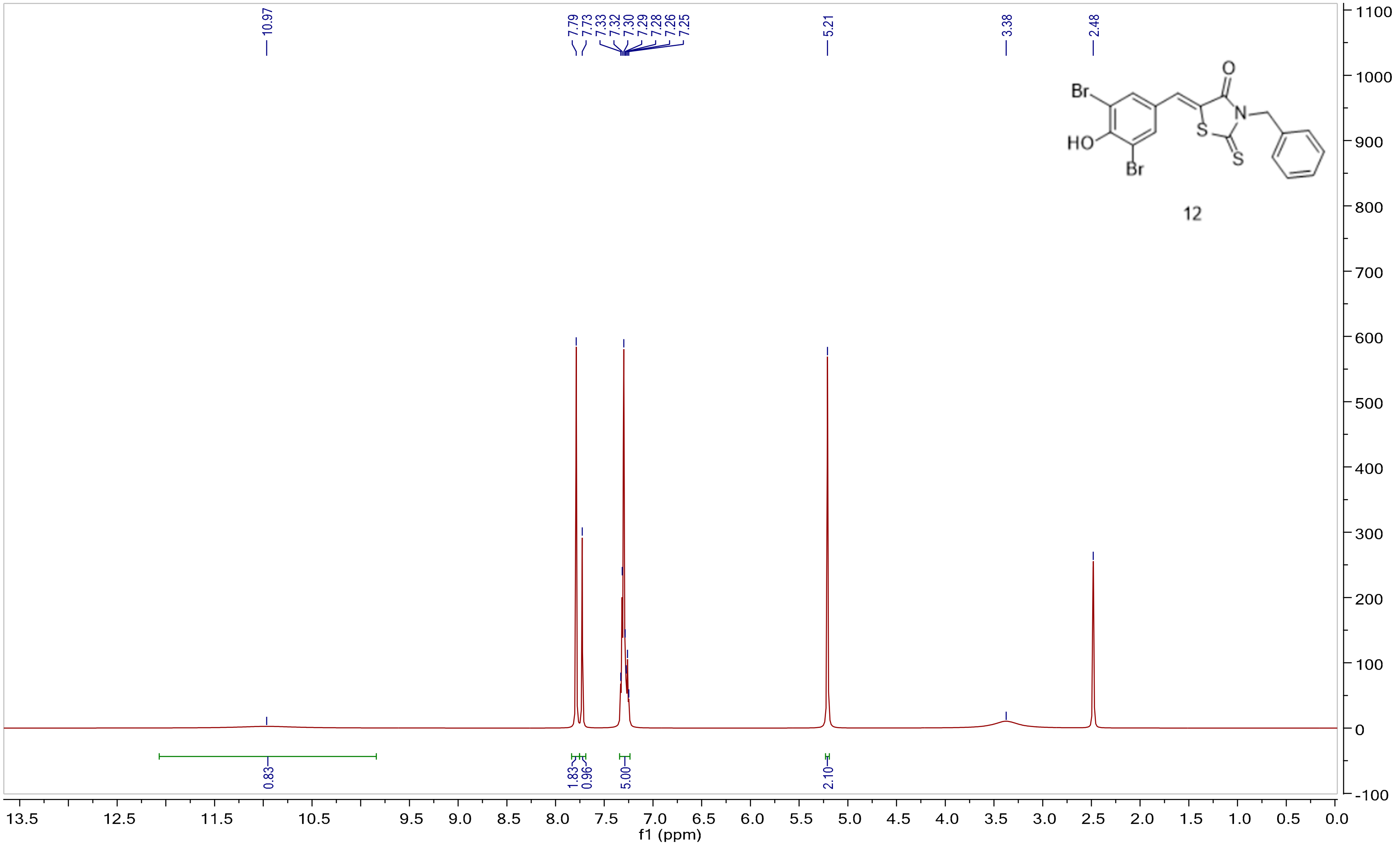

S47. ^{1}H NMR spectrum of analog **12**

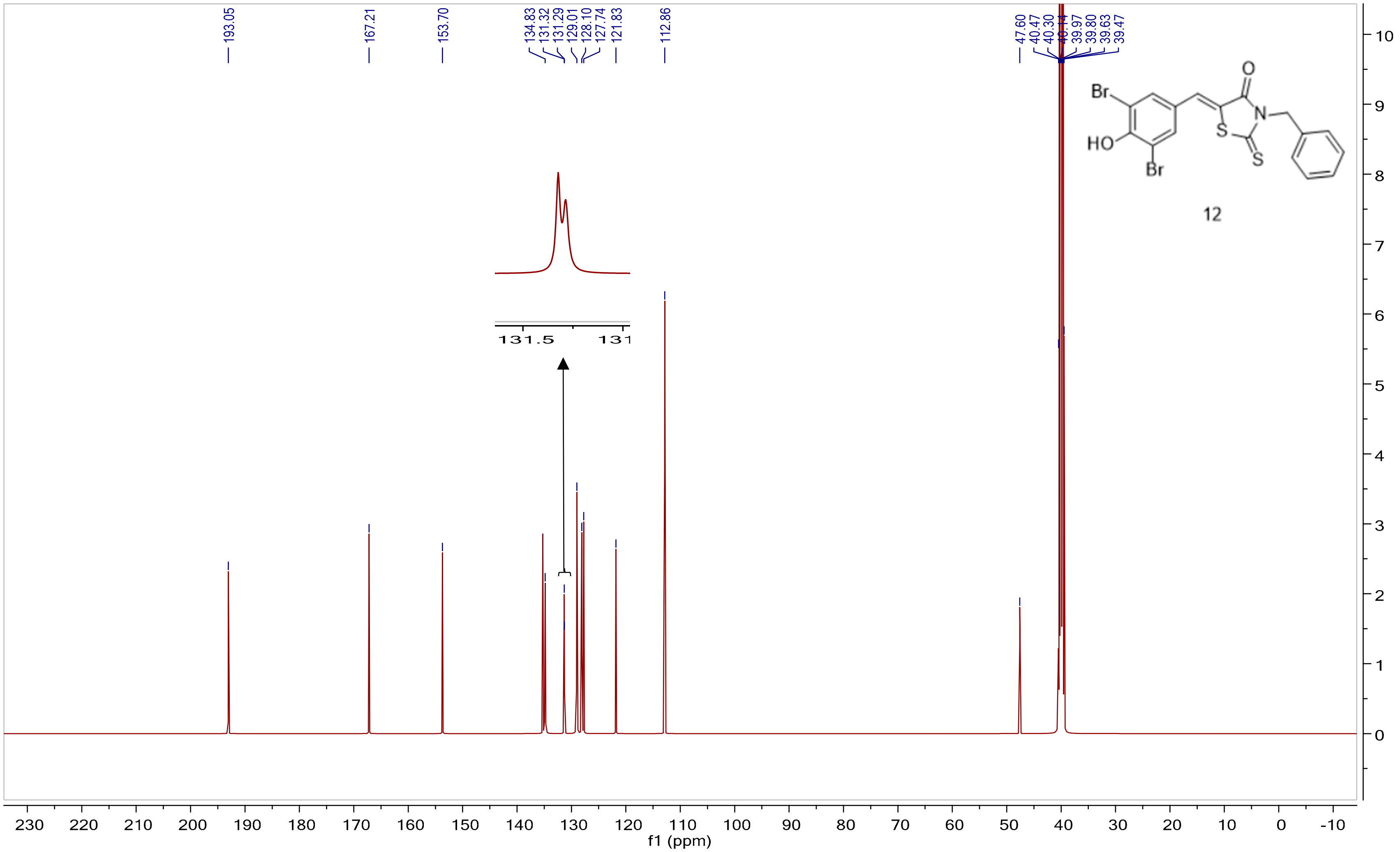

S48. ^{13}C NMR spectrum of analog **12**

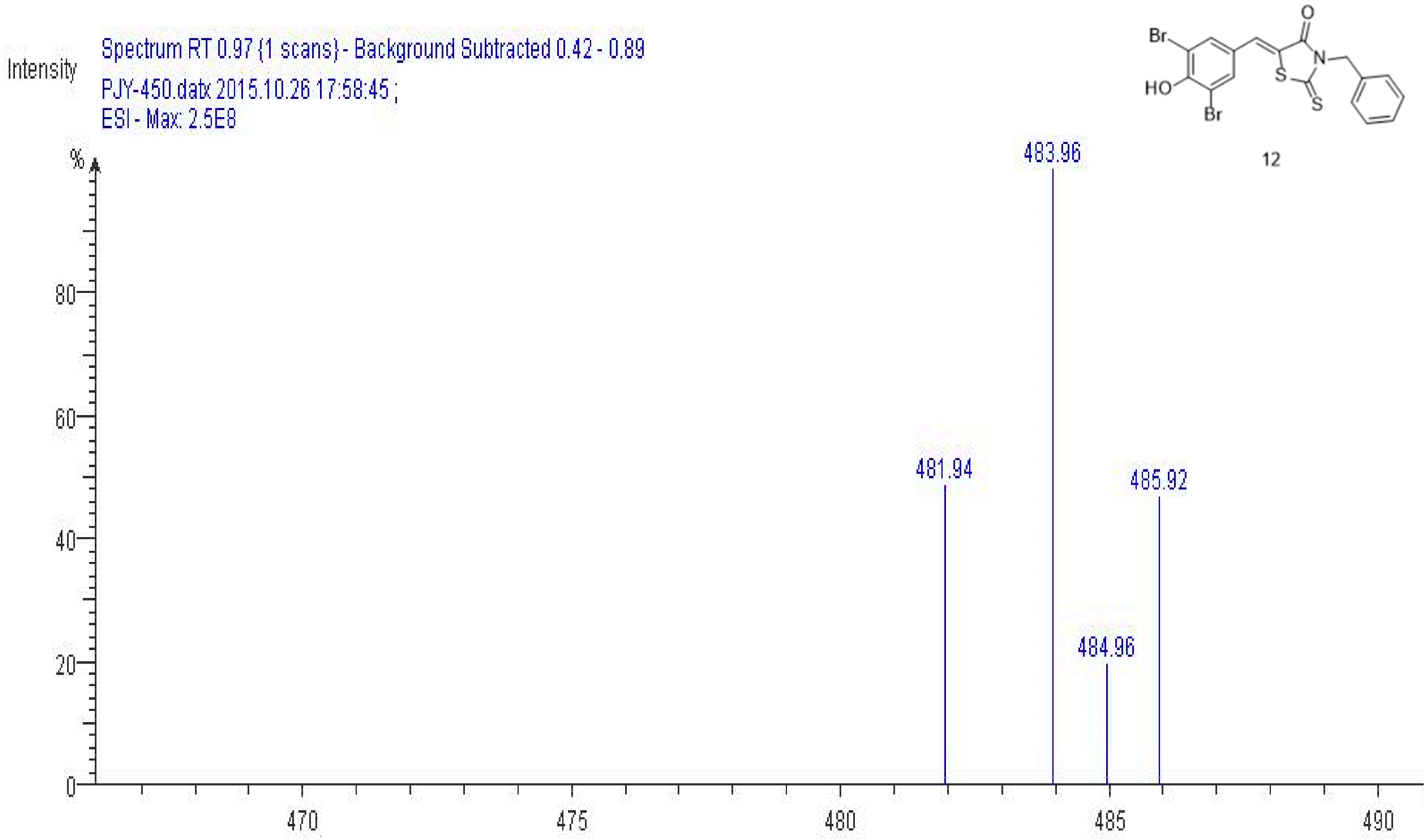

S49. LRMS (ESI–) spectrum of analog **12**
